# An experimental study of turtle shell rattle production and the implications for archaeofaunal assemblages

**DOI:** 10.1371/journal.pone.0201472

**Published:** 2018-08-02

**Authors:** Andrew Gillreath-Brown, Tanya M. Peres

**Affiliations:** 1 Department of Anthropology, Washington State University, Pullman, WA, United States of America; 2 Department of Anthropology, Florida State University, Tallahassee, FL, United States of America; University at Buffalo - The State University of New York, UNITED STATES

## Abstract

Turtle shell rattles are percussion instruments used by Indigenous peoples of the Americas in ceremonial contexts to keep rhythm. Archaeological investigations in the southeastern United States produced several complete and partial Eastern box turtle (*Terrapene carolina*) shell rattles from mortuary contexts dating from the Archaic (ca. 8000–1000 BC) through Mississippian periods (ca. AD 800–1500). Fragmentary turtle remains, some identified as Eastern box turtle, are frequently recovered from non-mortuary contexts. Traditionally, these fragmentary remains are attributed to food waste. Given the archaeological and ethnographic evidence for turtle shell rattles, we need to consider how fragmentary remains might fit into the chaîne opératoire of rattle production. This paper presents the results of an experimental study designed to identify one such chaîne opératoire of rattle production. During this experiment, the data on taphonomic processes such as manufacturing marks, use-wear, and breakage patterns, were recorded. We then tested the taphonomic findings from the experimental study and an object trait list we compiled from known rattle specimens and documentary sources with archaeological turtle remains recovered from non-mortuary contexts at two Mississippian period (ca. AD 1000–1450) sites in Middle Tennessee. Historic indigenous groups are known to have, and still do into the present-day, make and use turtle shell rattles in the region. Ultimately, we determined that “food refuse” should not be the default interpretation of fragmentary box turtle remains, and instead the taphonomic history and contextual associations must be considered in full. The experimental process of crafting turtle shell rattles enhances our understanding of an ancient musical instrument and the success rate of identifying musical artifacts and distinguishing between other modified turtle remains in the archaeological record. This study expands our knowledge of ancient music in North America and prompts re-analysis of curated turtle remains in museums for rattle-related modifications.

## Introduction

Music is a universal among human cultures across time and space and is thought to promote community building and cohesion [1–2]. While humans and human ancestors may have had the physical capabilities for music/musicality for a million years or more [3], the earliest identified musical instruments are bone and ivory flutes that date to approximately 36,000 years ago from Hohle Fels Cave in southern Germany [3–4]. Researchers argue that these flutes or pipes were sophisticated and likely part of a tradition that predates their origins and the arrival of anatomically modern humans in Europe [3]. Morley [3] notes that knowledge of the earliest musical instruments is limited by the lack of preservation of organic remains and the focus on the Paleolithic record of Europe over Africa or Australia.

We focus our attention on the limited understanding of the use of musical instruments by Indigenous groups in Native North America, specifically turtle shell rattles in the Eastern Woodlands. We know from ethnohistoric and ethnographic sources that Indigenous groups in the Americas craft(ed) and play(ed) musical instruments in various forms during community, celebratory, and ceremonial events. Percussive instruments used to keep rhythmic beat during singing or dancing are described in the ethnohistoric and ethnographic literature of the Americas. Drums and rattles fall into this category of instruments, and the latter are the focus of this paper. Across North America, rattles fashioned from turtle shells are a shared cultural object among many different Indigenous groups and are known from the archaeological and ethnohistoric records [e.g., 5–13]. More recently, turtle shells have sometimes been replaced with modern materials such as tin cans, wood, or copper, though the function is the same [14–17]. The ethnographic and ethnohistoric records detail the manufacture and use of turtle shell rattles in the present. Archaeological identifications of turtle rattles are scarce in the literature [9]. This is likely due to the manufacture of these instruments from perishable organic materials such as wood and bone, which precludes their preservation in the archaeological record. Additionally, archaeologists have tended to focus their analyses on durable material culture items such as lithics and ceramics to answer questions of social complexity, while animal remains are relegated to the subsistence category. This results in a lack of discussion or consideration of turtle shell rattles as a research topic or interpretative possibility.

Turtles played an important role in Indigenous cosmology, such as the belief that the world was formed upon the back of the Great Turtle [18–21]. This traditional beliefis held by several North American groups such as the Cherokee, Shawnee, Iroquois, Muscogee (Creek), and other tribes of the Eastern Woodlands, including the Algonkian. The Eastern box turtle (*Terrapene carolina*) is believed to be able to travel between the Above and Beneath Worlds, which is exemplified by their ability to move on land and in water. Turtles, such as Eastern box turtle, are the mediators between the two worlds, and for some groups are mediators between vegetarian and carnivorous animals [22–23]. For the Delaware of Oklahoma, the “Origin Myth also includes a man/woman opposition. Since the Turtle represents life and thereby consciousness, it also mediates between man and woman who also share consciousness between them” [24] (Speck 1937:24), also see [22]. Further, the False Face Society of the Seneca Iroquois “rub their turtle rattles on pine tree trunks, believing that thereby they become imbued with both the earth-power and the sky-power, … a recognition of the connection between the turtle and the world-tree that grows upon the primal turtle's back” [25] (Parker 1912:611), also see [22]. Consequently, turtle shell rattles are steeped in meaning of life and mediation, and are integral instruments in dances, ceremonies, and rituals.

**Fig 1:**
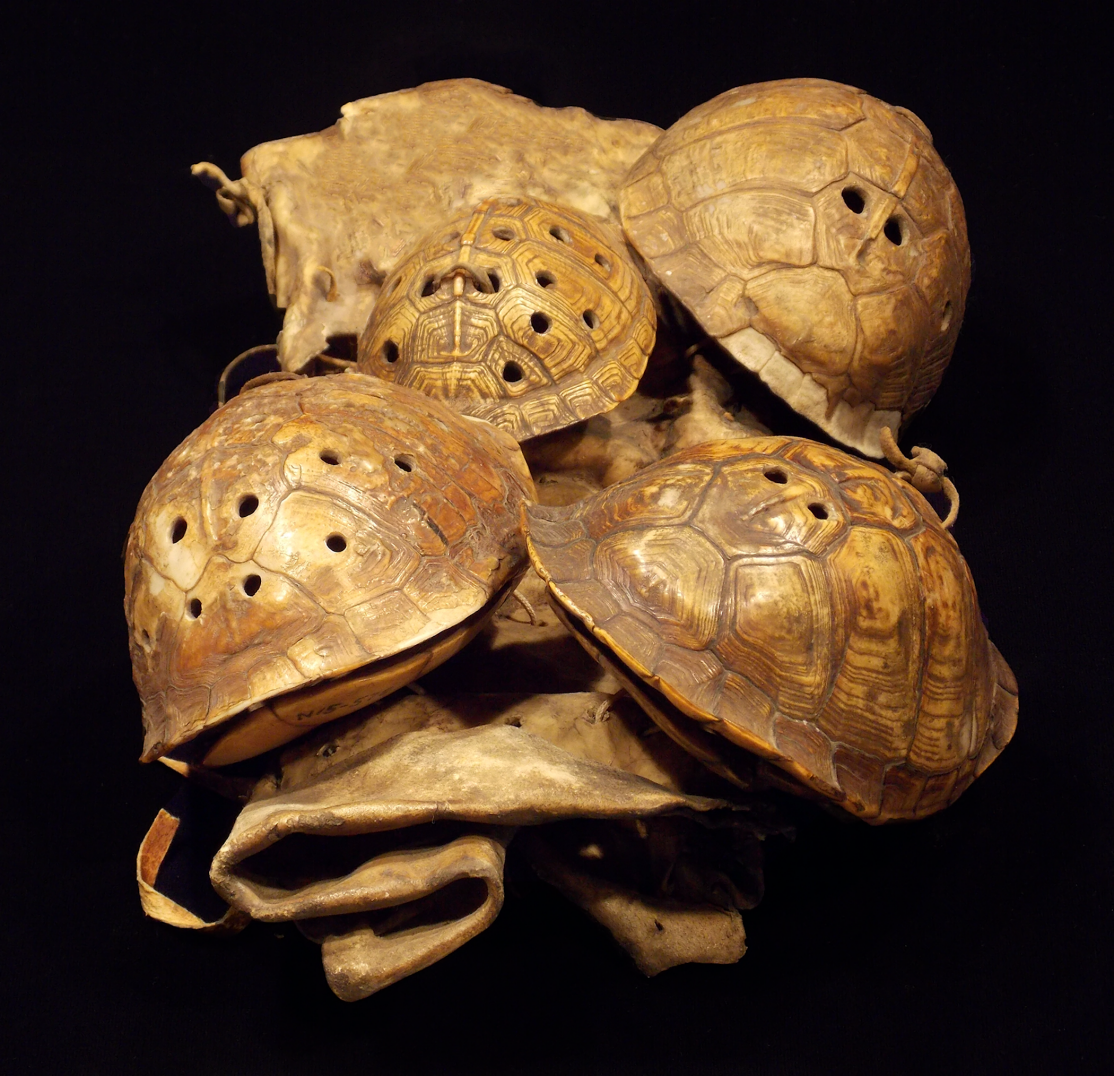
Example of historic turtle shell legging/shackle rattle. Four Eastern box turtle rattles tied to leather legging with leather string with stone flakes inside of the shells. Courtesy McClung Museum of Natural History and Culture, University of Tennessee, Knoxville, Tennessee (Catalog number 2011.27.26.1). Photo by Andrew Gillreath-Brown.

Traditionally, the women of the Muscogee (Creek) and Tsoyaha (Yuchi) tribes wear turtle shell leg rattles to keep the beat, which usually consist of eighth notes, along with drums (Fig 1) [26]. In the northern part of the Eastern Woodlands, O-non-dowa-gah (Seneca) musicians used turtle shell rattles in sacred ceremonies such as the great Feather Dance and False Faces [15]. Further, the O-non-dowa-gah used a variety of techniques to produce different rattle noises, such as the roll, snap, and crash [15] (Conklin and Sturtevant 1953:268–9). For example, “During tobacco invocations and during rites the [False] Faces may roll and snap their rattles or produce a scrape-roll” [15] (Conklin and Sturtevant 1953:269). For the great Feather Dance, two musicians hit rattles on a wooden bench with a duple crash. They also begin to sing in the first section, then dancing begins by others in the third song. The rhythms also become slightly more complex with iambic noise-level and duple crash.

**Fig 2:**
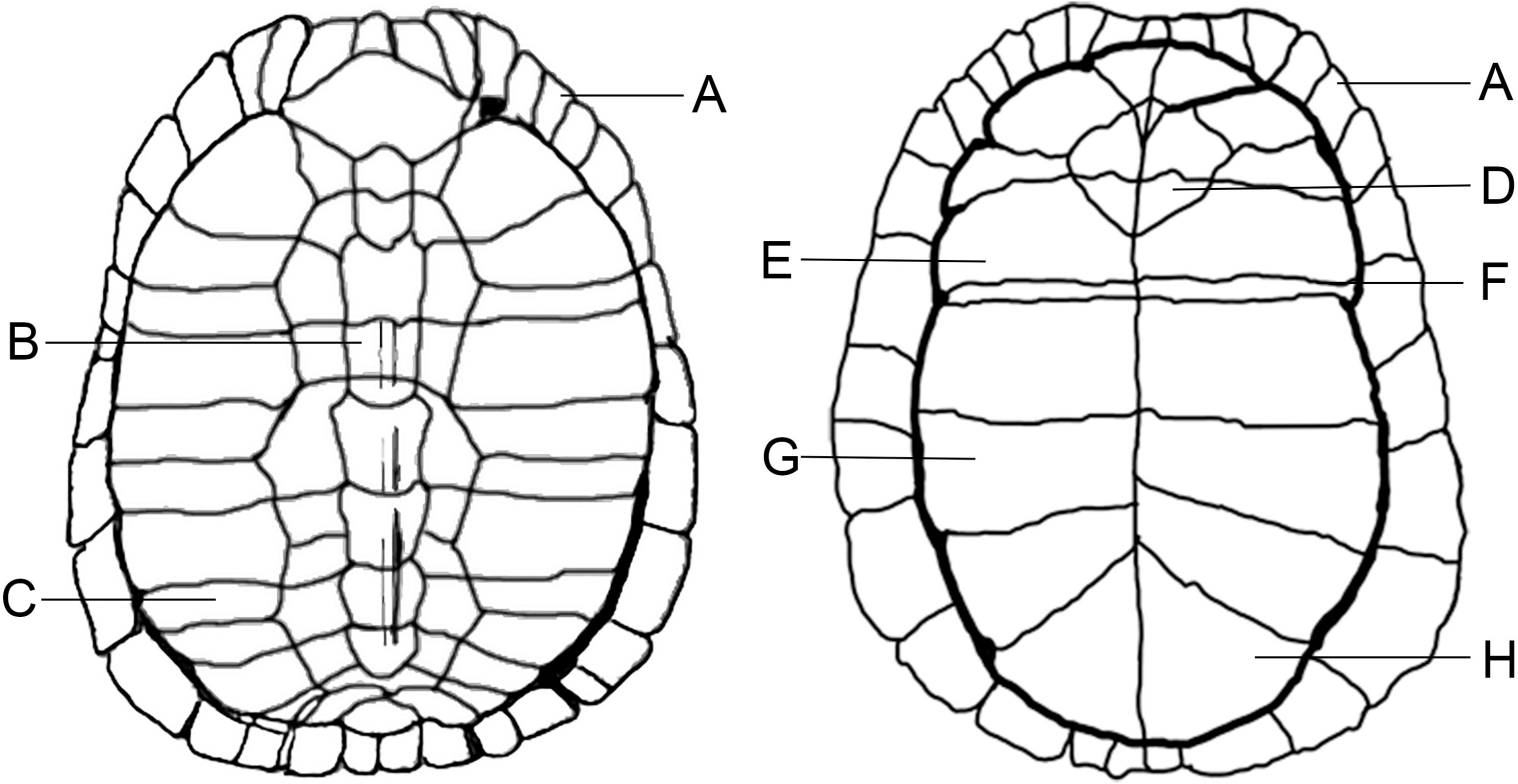
**Illustration showing basic elements of a turtle carapace (left) and plastron (right).** (A) Marginals, (B) Neurals, (C) Costals, (D) Entoplastron, (E) Hyoplastron, (F) Hinge, (G) Hypoplastron, and (H) Xiphiplastron.

Turtle shell rattles remain an important musical and ceremonial item for the modern-day Cherokee, Shawnee, Muscogee (Creek), Chickasaw Nation, and Seminoles. Rattle forms may vary culturally and geographically, thus we focus on the “body rattle” style as it is the most common type recovered from archaeological sites in the southeastern United States [9]. Two types of body rattles, legging/shackle and single shell, are known from the ethnographic, ethnohistoric, and archaeological records. The legging or shackle design consists of the turtle carapace (top shell) and plastron (bottom shell) tied together, attached to a piece of hide or fabric (Fig 1), also see [9]. The resulting “shackle” is comprised of multiple shell rattles (up to ten in some cases) then bound onto the dancer’s legs [9, 26]. To securely tie the top and bottom shells together, the craft person, typically a female, would drill five to seven holes inside the perimeter of the marginals on the top and the plastron on the bottom (Fig 2). The craft person would insert small objects, such as river pebbles (S16 and S17 Figs), molariform teeth from freshwater drum (*Aplodinotus grunniens*), or plant seeds, to make the distinctive rattle sound. The two pieces of shells were then closed and attached to the hide or fabric, using the same drilled holes. The second type, single shell design, is crafted similarly to the turtle shell leggings, except the turtle shells are not attached to hide. Instead, individual shells are tied to the arms or legs [8, 26]. Native Americans may have preferentially selected box turtles due to the structure of their shells.

Turtles have hard bony shells consisting of a bottom shell (plastron) and a top shell (carapace) (Fig 2) [27]. However, soft-shell turtles (Trionychidae) have fewer bones and a cartilaginous plastron. Turtle shell consists of several plate-like bones, which fit together along suture lines (the seam between the plates) (Fig 2). Epidermal scutes—made of a superficial layer of keratin—are staggered over the bony plates, providing additional protection and strength to the shell (S8 Fig). The sutures tend to be the weaker points of a shell. As turtles age, the bony plates thicken and fuse. The Eastern box turtle spends most of its time on land. It also has a hinge or modified suture, on the plastron (Fig 2). This allows the turtle to completely enclose itself within the shell. This hinge may have been one of the reasons Indigenous peoples preferred box turtles for making rattles. It enabled the shell to be more tightly sealed for a rattle over other types of turtle shells without using additional materials (e.g., leather).

While the body rattles are used primarily by women, it is unclear who may have captured the turtles. Unfortunately, the process leading up to rattle construction is generally not recorded in the ethnographic record. However, since it would be preferable to keep box turtle shells in one piece (especially the hinge), it is unlikely that the turtle shell would have been broken apart or boiled to extract the meat, although limbs could easily be trimmed off. The symbolic meaning of box turtles for rattles may have kept the turtle from being consumed, although a non-rattle turtle could be consumed. Cooking or processing would cause the shell to be in at least three pieces (one carapace and two plastron pieces). The consumption of box turtles could lead to sickness, given that box turtles consume mushrooms that are poisonous to humans [8, 28–29]. The boiling process could also damage or weaken the shell, particularly along the sutures. Some modern Muscogee (Creek) people use dead box turtles and place them in large red ant piles, which allows for ants to eat away the meat inside of the shell, while keeping the carapace and plastron, including the hinge, intact [8]. The turtle shell stays as one entity without having to bind the pieces back together. However, keeping the shell as one piece may have been less likely for northeastern snapping turtle (*Chelydra serpentina*) rattles [15]. The meat may have been saved for cooking, while the shell was also used to construct a rattle.

### Turtle shell rattles in the archaeological record

**Fig 3:**
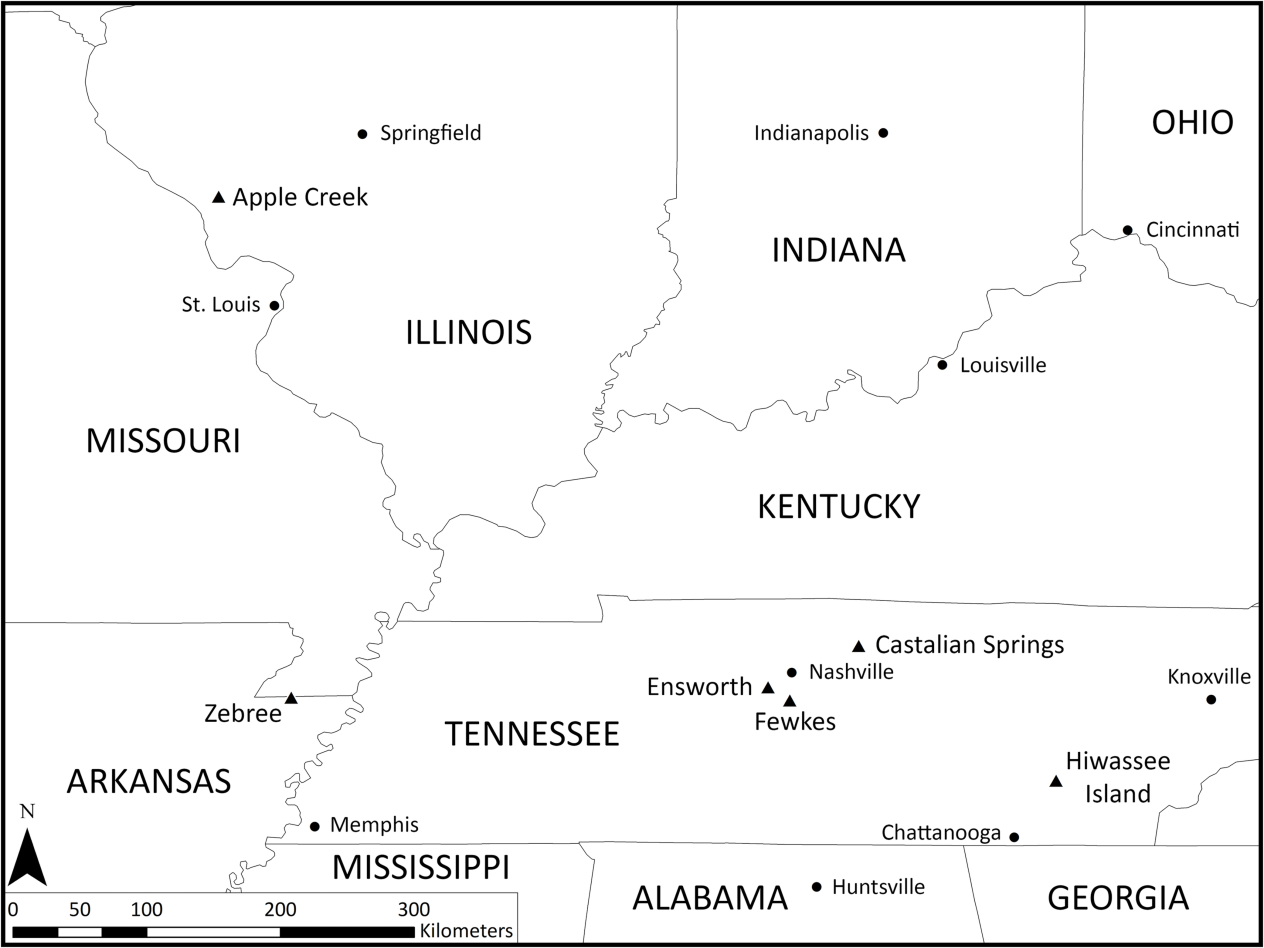
Archaeological sites mentioned in the text. Circles indicate modern cities and triangles indicate archaeological sites.

In the southeastern United States, turtle shell rattles have been recovered primarily from mortuary contexts dating to the Archaic (8000–1000 BC), Woodland (1000 BC–AD 800), and late prehistoric Mississippian periods (AD 800–1500) (e.g., [8, 30–35]). Body rattles were recovered from various contexts at several archaeological sites in the eastern United States (Fig 3), including mortuary contexts at Ensworth in Middle Tennessee [31, 36] and Hiwassee Island in East Tennessee [32]; Apple Creek in Illinois, where the context was not reported [34]; and on a house floor at Zebree in Arkansas [33, 37]. In Tennessee, body rattles are most often identified from the lower leg area of human burials. Notably, most of these individuals are female.

**Fig 4:**
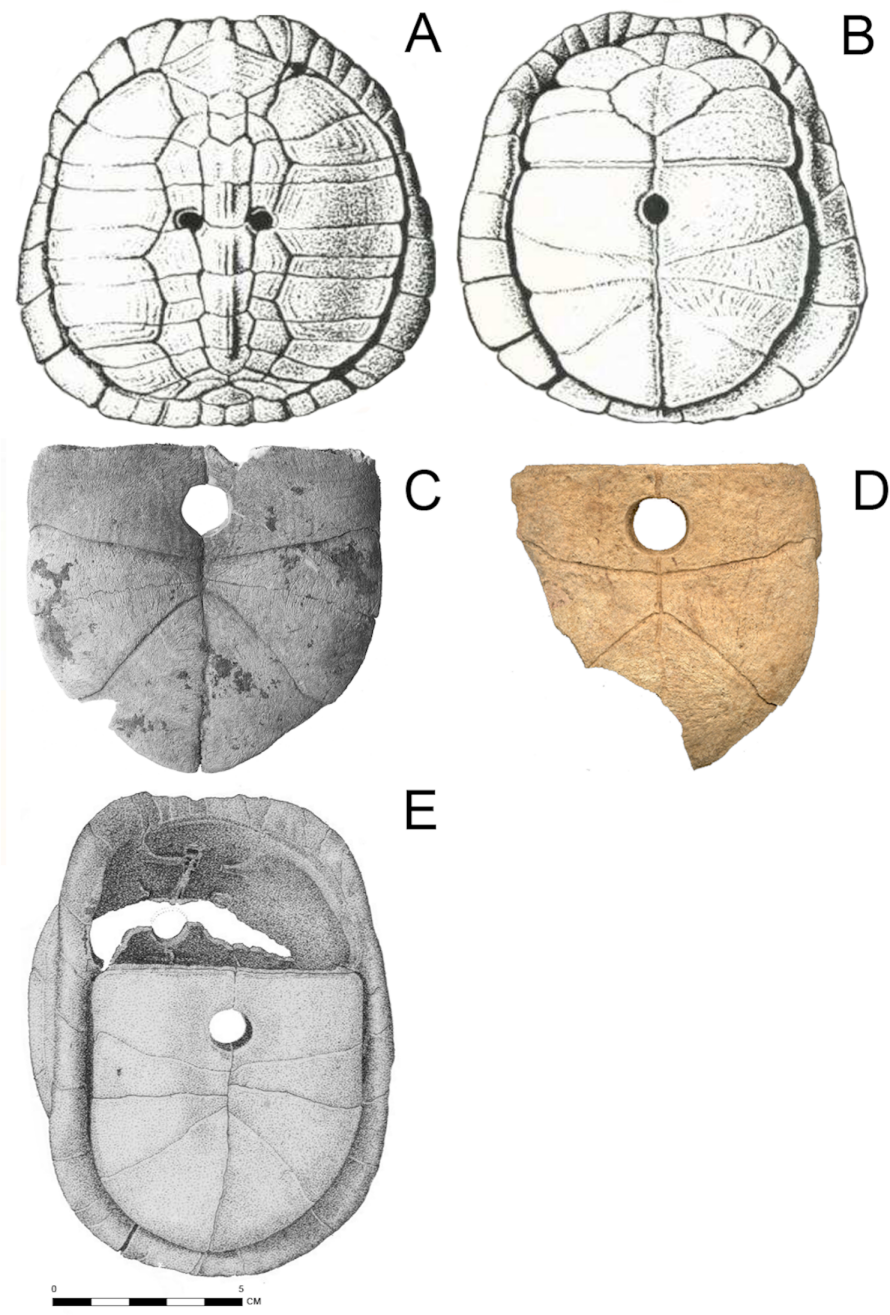
Four archaeological turtle shell rattle specimens used as design templates for the experimental rattle and Drill Holes. (A-B) Hiwassee Island [32] (Lewis and Kneberg 1970:126), (C) Apple Creek [34] (Parmalee et al. 1972:29), (D) Ensworth [31], and (E) Zebree [37–38] sites.

If an individual was buried with more than one turtle shell rattle per leg, we may assume these were lashed together on a single piece of hide or fabric in the shackle fashion. For example, Lewis and Kneberg [32] noted ten turtle shell rattles in a pile around a female’s lumbar region buried at Hiwassee Island in East Tennessee, suggesting that these were meant to be worn as shackles or leggings. At the Ensworth site, several turtle fragments were recovered from a single burial [31]. The identifiable portion of the Ensworth rattle consisted of the hypoplastron of the Eastern box turtle, which had one drilled hole (Fig 4D, see Fig 2 for turtle elements). Other highly polished turtle carapace fragments were recovered from the burial, which may have been part of the same rattle. The Zebree site is in Mississippi County, Arkansas (Fig 3). A turtle shell rattle was found on a middle Mississippian (ca. AD 1200–1400) house floor (Fig 4E) [33, 37–38]. Pebbles made a trail on the floor in between the carapace and plastron. The Apple Creek site is in Greene County, Illinois and was mostly occupied during the Middle to Late Woodland period (ca. AD 150–750) [34] (Fig 3). Eighteen turtle shell fragments had drilled holes. However, the context of the rattle fragments is not reported.

Complete, partial, and fragmentary turtle bones of various species are often recovered during archaeological excavations. Further, many turtle remains are excavated from middens, trash pits, and domestic deposits. However, outside of mortuary contexts, it is rare for archaeologists to identify fragmentary turtle specimens as pieces of turtle shell rattles in the archaeological record. Equifinality is a major contributor to this issue. Generally, turtle shell rattles are recorded when it is easy or obvious to do so, based on context (typically, but not always, found with human burials), representation (intact or mostly intact carapace and plastron), modifications (drill holes for bindings), and associated rattle objects. The difficulty comes when archaeologists are presented with fragmentary turtle remains from non-mortuary contexts. Turtle specimens recovered from trash pits, floor deposits, and other domestic deposits may be the result of food waste, musical instrument crafting or breakage, or some other type of artifact [9]. However, turtle remains recovered from these contexts often include limb bones, vertebral, and cranial elements of multiple taxa. These elements may also exhibit evidence of burning, either as a result of food cooking or trash disposal. It is not often that turtle specimens recovered from these contexts are interpreted as anything other than food waste.

In this paper, we argue that the identification of turtle shell rattle fragments in archaeological contexts can be achieved through multiple lines of evidence, including experimental archaeology, ethnographic and ethnohistoric documents, and modern turtle shell rattles. Experimental archaeology can provide a way for understanding the crafting process of turtle shell rattles, and the resulting patterns and characteristics of modifications and breakage (S1 Fig, S1 Video). From ethnographic, ethnohistoric, archaeological, and modern examples of turtle shell rattles, we formulated a plan to create an experimental turtle shell rattle. Finally, we use an object trait list for rattles [9] and the rattle-crafting characteristics from the experimental process to identify any rattle-related modifications on archaeologically recovered turtle remains from two Mississippian period sites in Middle Tennessee, Fewkes (40WM1) and Castalian Springs (40SU14).

## Experimental archaeology: Crafting of turtle shell rattles

**Fig 5:**
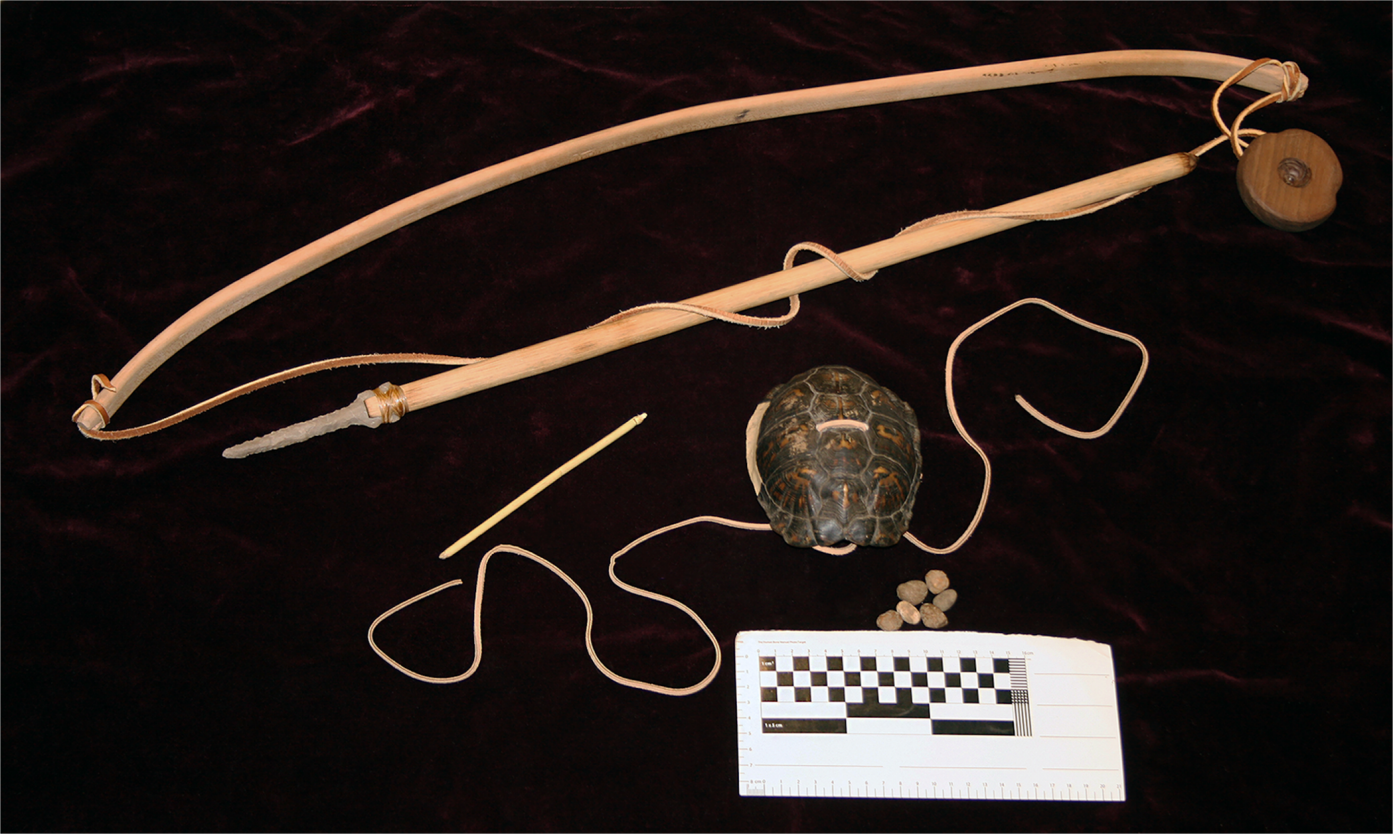
Tools and samples used in experimental study. Items include bow and chert drill, the drilled carapace (Drill Holes 5 and 6), river pebbles, and river cane.

Experimental archaeology is the process of recreating ancient artifacts or structures to identify possible methods of production, use, and wear of past technologies [39–40]. Reynolds [41] (1999:158–62) defined five types of experimental archaeology including construct, processes and function experiments, simulation, eventuality trial, and technological innovation [42]. For example, experimental archaeology could reveal how to construct a particular artifact type. Experimental archaeology can also suffer from equifinality. Although a researcher may find one valid path to creating an artifact, it is possible that there are many other tools and/or techniques that could be used to get to the same end result. Previous experimental studies of ancient life in the southeastern United States have focused on topics such as architecture [43], distinguishing between cultural or natural presence of small animal remains in pits [44], tattooing [45], pottery [46], and flintknapping [47]. For this project, experimental archaeology was used to understand the turtle shell rattle manufacturing process and identify the archaeological correlates of the process. We expected that drilling and using the shell as a rattle would result in identifiable marks including drill marks, breakage patterns, and the impact of river pebbles on the interior of the shell. Our goals were to re-create and document the crafting process of one turtle shell rattle type, the single shell body rattle, and record potential archaeological correlates (Fig 5). The results of the experimental study were then compared with fragmentary turtle specimens identified from two archaeological sites located in Middle Tennessee, an area in which turtle shell rattles were previously recovered from the archaeological record.

### Experimental study approach and design

Multiple lines of evidence are needed to distinguish turtle shell rattles from other turtle shell artifacts, such as those used for bowls or as debris from food processing and consumption [9]. Gillreath-Brown and Peres [9] consulted the archaeological, ethnographic, and ethnohistoric records and developed an object trait list to assist with identifying fragments of turtle shell rattles from archaeological contexts, particularly in the southeastern United States [8]. The trait list includes five characteristics [9]: (1) archaeological context (e.g., ceremonial, ritual, or burial); (2) turtle species; (3) skeletal element representation (only carapace and plastron represented with no other bones); (4) modifications (e.g., drill holes, polish); and (5) associated rattle objects (e.g., pebbles (S16 and S17 Figs), seeds, freshwater drum molariform teeth). Further, we note the presence of residue staining, which could come from materials such as pine pitch or red ochre. In the southwestern United States, asphaltum was used on rattles to fill in openings or cement the shell together [48–49]. Pine pitch is a glue-like adhesive that is made from raw pine pitch, fine ground charcoal, and plant materials [50–51]. It is also possible that red ochre was used as a coloring agent on the rattle. Experimental rattles were created to understand the rattle object and modification traits.

**Fig 6:**
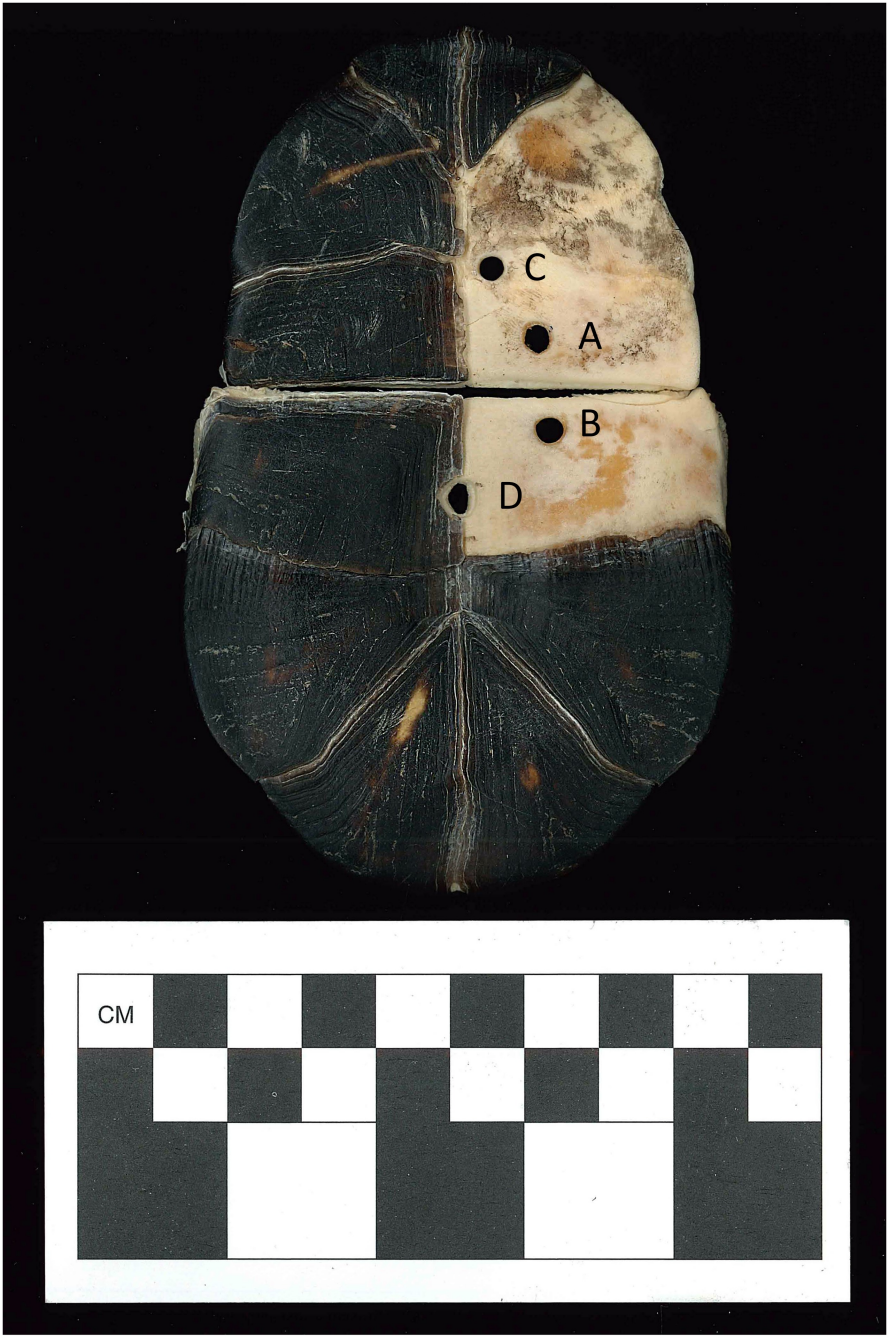
Experimental plastron (Drill Holes 7–10) showing the four drilling locations. (A) Drill Hole 7, (B) Drill Hole 8, (C) Drill Hole 9, and (D) Drill Hole 10.

Turtle shell rattles recovered from the Ensworth [31], Apple Creek [34], Hiwassee Island [32], and Zebree [37–38] archaeological sites (Fig 3) provided the main design for the experimental rattle (Figs 4 and 5). Although these sites represent three different archaeological periods (Archaic, Woodland, and Mississippian), their inclusion in the study is valid as these are all unequivocal rattles. Two of the sites, Hiwassee Island and Zebree, date to the Mississippian period. The Apple Creek rattle, which dates primarily to the Woodland period, is included because it exhibits similar qualities to rattles at the other three sites [34]. The example from Ensworth, which primarily has an Archaic occupation [36], is included because it exhibits the same drill placement on the plastron as the two Mississippian sites (Fig 4). For the experimental plastron (Drill Holes 7–10), three additional holes were drilled to reconnect the hinge area and connect the plastron to the carapace (Fig 6A–6C). The reason for tying the hinge together was a result of not being able to attain a fully-intact box turtle specimen.

#### Drilling instruments

Based on ethnographic, archaeological, and previous experimental studies, two types of drilling implements were tested in this study: river cane and sand (S2 Fig); and bow and chert drill point (Fig 5). Previous experimental studies have shown that river cane can successfully drill stone and other objects [52–57]. A method of using a bow with the river cane was also attempted, which failed to operate effectively. It was not possible to maintain stability of the river cane or to keep the river cane in one place on the shell when using the bow. The last attempt with river cane was to begin a small hole with a chert drill, then use the river cane. This was attempted because it made the river cane more stable. This attempt also resulted in failure to drill the shell, as it was not able to deepen the hole started by the chert drill.

The second method was to use a bow and chert drill bit (Fig 5, S1 Fig, S1 Video). The bow and wooden staff were made from pine, the sinew was artificial, and the drill (thickness: 5.82 mm (at bit), width: 8.76 mm (at bit), length: 91.04 mm) was made from locally-available and abundant Ft. Payne chert. This was the most effective and efficient implement of the two (S1 Fig). However, it was easier to manually start the drill hole with the wooden staff and drill, which provided stability, then add the bow.

#### Rattle objects

River pebbles [32, 58], freshwater drum teeth [32, 59], and hard seeds [14] are all cited in the relevant literature as objects used to create the rattle effect. We chose river pebbles collected from the Stones River, located in Murfreesboro, Tennessee (S16 and S17 Figs). Eight pebbles were placed inside of the rattle. The pebbles are river worn sedimentary rock and range in size from approximately 4 mm to 8 mm.

#### Drilling process

**Fig 7:**
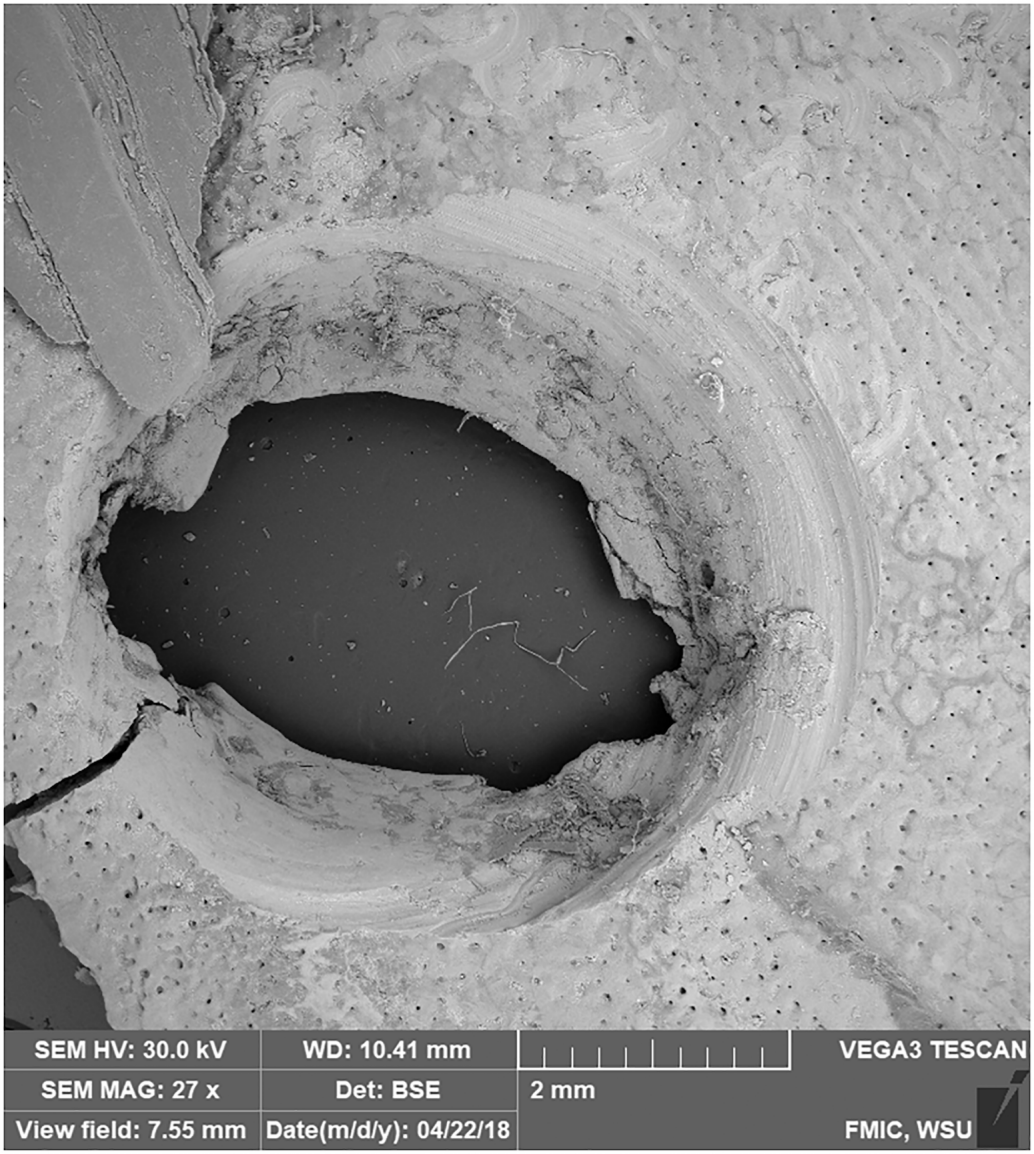
SEM micrograph showing the circular to oval shape on Drill Hole 2. Micro-striations are also visible in and around the hole. Image was captured on a Tescan Vega 3 SEM at 27x magnification. Bar = 2mm.

**Fig 8:**
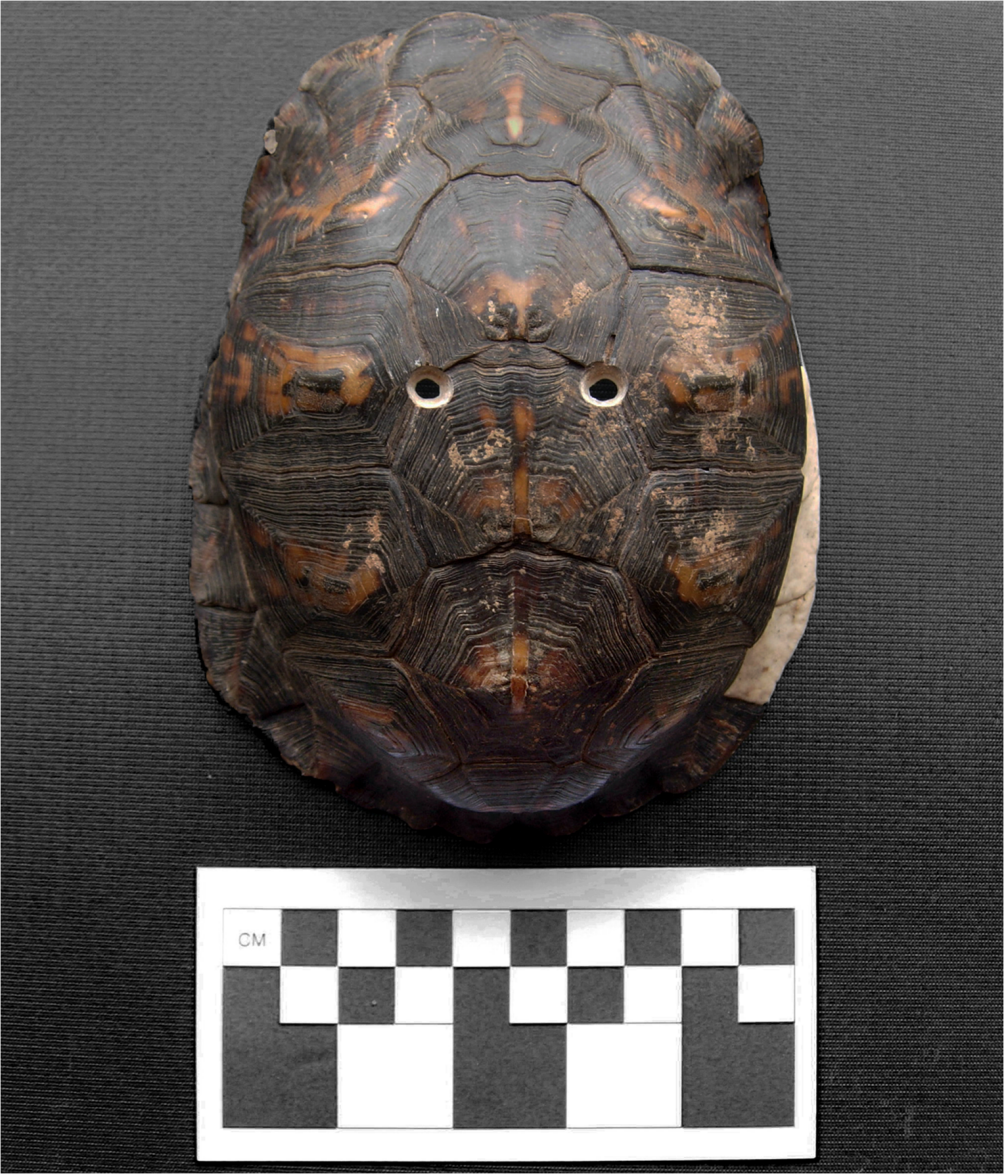
Holes drilled into turtle carapace (Drill Holes 5 and 6). The darker (browner) part of the shell are the scutes and the whiter (or lighter) areas on the shell (on the right) are the marginal bony plates. Also see S1 3D Model.

During the experimental crafting portion of this project, information on drilling techniques and modification were recorded through photography, video, stereomicroscope, and scanning electron microscopy (SEM) (see S1 Text for specimen collection and methods for documentation, S1 Video). No permits were required for the current described study, which complied with all relevant regulations. The modern box turtle specimens used for this study died of natural causes and were collected post-mortem. The drilling process left a characteristic concave-oval shape, when the drill angle was not changed (Figs 6D and 7). When the angle of the chert drill was changed 90 degrees, the oval shape changed to a concave circle (Figs 6 and 8, S1 3D Model). The pressure from the chert drill can cause distinct breakage patterns, such as sharp angled breaks with drill indentations (S12 and S15 Figs). A complete description (or main drill description) of the drilling process of Drill Holes 1–10 is presented in S1 Text. The information gained from the experiment and the trait list [9] was used to assess turtle remains recovered from two Mississippian period sites in Middle Tennessee.

## Experimental Results

### Experimental drill measurements

Final measurements were taken for the ten experimental Drill Holes (Table 1). For the original drill side (or first side that was drilled), measurements were consistent, ranging from 5.0 mm to 7.0 mm for the left-right axis, excluding Drill Hole 9 (4.5 mm). Also, the anteroposterior axis consisted of a range of 5.1 mm to 6.4 mm, excluding Drill Hole 9 (4.6 mm). For the other side, the left-right axis yielded a range of 2.1 mm to 4.5 mm, excluding Drill Hole 4 (5.3 mm). Also, the anteroposterior axis consisted of a range of 3.4 mm to 5.6 mm, excluding Drill Hole 4 (6.0 mm). Measurements could vary depending on the size and type of drill, and length of time that each hole is drilled.

**Table 1:** Measurements of drill openings of the experimental drill holes. The left-right axis refers to the horizontal when the turtle shells are oriented with the head of the shell at the top of the image, such as Figs 2 and 8. The anteroposterior is along the vertical axis.

		Primary Drill Side		Secondary Drill Side	
Drill Hole	Element	Left-Right (mm)	Anteroposterior (mm)	Left-Right (mm)	Anteroposterior (mm)
**1**	Marginal	N/A	6.09	N/A	N/A
**2**	Costal	6.08	6.37	3.78	4.23
**3**	Costal	4.97	N/A	2.06	N/A
**4**	Carapace	5.73	6.17	5.31	5.99
**5**	Carapace	6.70	6.02	3.75	3.35
**6**	Carapace	6.99	6.34	4.36	3.42
**7**	Hyoplastron	5.12	5.14	4.00	4.30
**8**	Hypoplastron	5.93	5.87	4.20	4.09
**9**	Hyoplastron	4.49	4.63	3.94	3.54
**10**	Hypoplastron	5.35	5.23	3.47	4.58

### Drill hole wear patterns

SEM was used to document the wear patterns in the drill holes from the bow and chert drill (Fig 7). The chert drill creates stratigraphy moving from the top to the bottom of the drill hole. At the top, the striations tend to be closer together and deeper (S34 and S35 Figs). Towards the middle and bottom, the striations move farther apart and become shallower (S36 and S37 Figs). Additionally, striations or scraping around the drill hole may be evidence of unintentional modification (S38 Fig). During the experimental process, this happened for Drill Hole 2 when the drilling process began and the drill bit slipped from its starting location (Fig 7).

### Breakage patterns

An increased amount of drilling increased the probability of the turtle shell breaking, as seen with Drill Hole 4. The shell began to break or split apart close to the sutures for the juvenile turtle carapace. The outcome could potentially be different for a fully-fused adult turtle carapace, or one that was not already cracked. Therefore, it is reasonable to assume that adult specimens were more likely chosen as raw material for rattles. Applying pressure from the drill can cause breakage, which may result in very sharp-angled fragments (S14 and S24 Figs). The pressure from the break can also result in indentations left by the drill (S12 and S15 Figs). Sharp angles with the presence of drill indentations could be evidence for turtle shell rattles. The presence of sharp-angled breaks on its own does not necessarily merit interpretation of a fragment as a rattle, as other lines of evidence are needed, such as meeting criteria in the object trait list [9]. Other modified objects (such as bowls, cups, medicine bags) or different construction methods may cause similar sharp-angled breaks. In summary, the chert drilling method for rattles can be identified by the combination of both the presence of sharp breaks and drill indentations.

### Rattle objects

After shaking the rattle approximately 350 times, or for four minutes, no modification was present on the carapace interior (S33 Fig). Ethnographic studies have documented dances lasting for hours, some overnight (12+ hours) [60], which would supersede 350 shakes. Thus, further testing of the rattle for longer periods would increase the chance that use-wear on the interior would appear from the rattle objects (in this case, pebbles) abrading against the surface [61–62].

## Applying the experimental study and trait list to archaeofaunal collections

The experimental study provided additional insights into the trait list [9], particularly for modifications and when turtle shells fragment or break during the rattle-making process. Fragmented turtle shells could have characteristics that suggest rattle manufacture, such as sharp-angled breaks with drill indentations or striations around a drill hole. The experimental drilling process also showed the typical characteristics of the drill hole—the concave oval or circle shape with some micro-striations inside the drill hole—for rattles. Therefore, the experimental information provided additional characteristics to anticipate on archaeological turtle remains. Additionally, we use the drilling process to evaluate other types of holes or perforations in turtle shell in the archaeological record, as these would likely be interpreted as different artifact types. Here we apply the experimental data and trait list to archaeological turtle remains recovered from two Mississippian period sites, Fewkes (40WM1) and Castalian Springs (40SU14), located in Middle Tennessee.

The Fewkes and Castalian Springs sites were relatively contemporaneous sites situated at opposite ends of the Nashville Basin, during the Mississippian period and date to approximately AD 1250–1450 [63] and AD 1150–1300, respectively [64–66] (Fig 3). The Fewkes site is located in Williamson County, Tennessee, situated along the Little Harpeth River (Fig 3) [67–70]. The faunal assemblage recovered from Fewkes was mainly from contexts associated with the village area, though several features connected to ritual activities at the site yielded animal remains [63, 71]. The faunal assemblage from the Fewkes site (40WM1) is housed at the Tennessee Department of Transportation, Nashville, Tennessee. Castalian Springs is located close to a mineral spring and tributary of the Cumberland River, and covers approximately 8 ha [64, 72] (Fig 3). The village contained at least four mounds, the largest of which was the platform mound that was once 200 feet in length and 11 feet in height [70]. The faunal assemblage from the Castalian Springs site (40SU14) is curated by the Tennessee Division of Archaeology, Nashville, Tennessee.

The majority of the identified turtle remains from both sites are made up of Eastern box turtle carapace and plastron specimens (75% MNI at Fewkes and 45.5% MNI at Castalian Springs) (Table 2) [63, 73]. We follow the most recent taxonomic assignment of chelonian taxa [74]. A total of 167 fragmentary turtle remains from Fewkes and 32 fragmentary turtle remains from Castalian Springs are included in the present study. Most of the turtle specimens noted as having some sort of modification are identified as Eastern box turtle. None of these remains are from human burials and their fragmentary nature makes them difficult to definitively assign function, hence the current study. Modifications to turtle bones occur at both sites, and as detailed below some modified turtle remains exhibit two to three of the five turtle shell rattle object traits (Tables 3–5) [9]. Some modified elements were identified as taxa other than box turtle, including pond slider (*Trachemys scripta*), softshell turtles (Trionychidae), and water or box turtles (Emydidae). While they are not the preferred species (i.e., Eastern box turtle) for use in the construction of rattles, they do conform to the expectations for skeletal representation (Trait 3) and modification characteristics (Trait 4) of turtle shell rattles.

**Table 2:** Summary of turtle remains from Fewkes and Castalian Springs.

**Fewkes**^**a**^						
	**Turtle Taxa**	**Common Name**	**NISP**	**Weight**	**MNI**	**%MNI**
	Testudines	turtles	877	294.11	0	0
	Kinosternidae	mud and musk turtle family	62	18.1	1	5
	Emydidae	water and box turtle family	66	43.8	0	0
	*Terrapene carolina*	Eastern box turtle	559	529.24	15	75
	*Pseudemys concinna*^*b*^	river cooter	1	1.94	1	5
	*Chrysemys picta*	painted turtle	6	5.5	1	5
	*Trachemys scripta*	pond slider	1	1.03	1	5
	*Pseudemys/Trachemys/Chrysemys* spp.	cooter, slider, and painted turtles	10	16.01	0	0
	*Apalone* sp.	softshell turtle	2	0.72	1	5
**Total turtles identified at Fewkes**			**1584**	**910.45**	**20**	**100**
**Castalian Springs**^**c**^						
	**Turtle Taxa**	**Common Name**	**NISP**	**Weight**	**MNI**	**%MNI**
	Testudines	turtles	142	49.55	0	0
	*Chelydra* sp.	snapping turtle	3	7.34	1	9.1
	Kinosternidae	mud and musk turtle family	10	2.76	2	18.2
	*Sternotherus odoratus*	musk turtle	6	4.58	1	9.1
	Emydidae	water and box turtle family	27	26.42	0	0.0
	*Chrysemys/Graptemys* sp.	painted and map turtles	1	1	1	9.1
	*Trachemys scripta*	pond slider	3	13.9	1	9.1
	*Terrapene carolina*	Eastern box turtle	315	250.4	5	45.5
**Total turtles identified at Castalian Springs**			**507**	**355.95**	**11**	**100**

^a^Fewkes turtle data are from [63].

^b^In the curated database, this species was called *Pseudemys floridana*.

^c^Castalian Springs turtle data are from [73].

**Table 3:** Fewkes associated rattle modifications (features only).

	Feature 55		Feature 184	Feature 549	Feature 702	Feature 817	Feature 818
	Emydidae	*T*. *carolina*	Testudines	Testudines	*T*. *carolina*	*T*. *carolina*	Trionychidae
**Partial Drill (exterior)**	0	1	0	1	0	0	0
**Red Ochre (interior)**	1	0	0	0	0	0	0
**Complete Drill**	0	0	0	0	1	1	0
**Perforated**	0	0	0	0	0	0	1
**Worked**	0	0	0	1	0	0	1
**Snapped**	0	0	1	1	0	0	0
**Total**	1	1	1	3	1	1	2

**Table 4:** Fewkes associated rattle modifications (including features).

	Emydidae	*T*. *carolina*	Testudines	Trionychidae
**Partial Drill (exterior)**	0	1	1	0
**Red Ochre (interior)**	1	2	4	0
**Red Ochre (exterior)**	0	5	1	0
**Complete Drill**	0	1	1	0
**Perforated**	0	1	1	1
**Worked**	0	6	1	1
**Snapped**	0	0	2	0
**Ground**	0	6	0	0
**Polish**	0	1	0	0
**Indentation (interior)**	0	4	8	1
**Indentation (exterior)**	0	3	4	0
**Total**	1	30	23	3

**Table 5:** Castalian Springs associated rattle modifications.

	*T*. *carolina*	*T*. *scripta*
**Complete Drill**	0	1
**Snapped**	1	0
**Ground**	2	0
**Polish**	8	0
**Total**	11	1

### Fewkes

**Fig 9:**
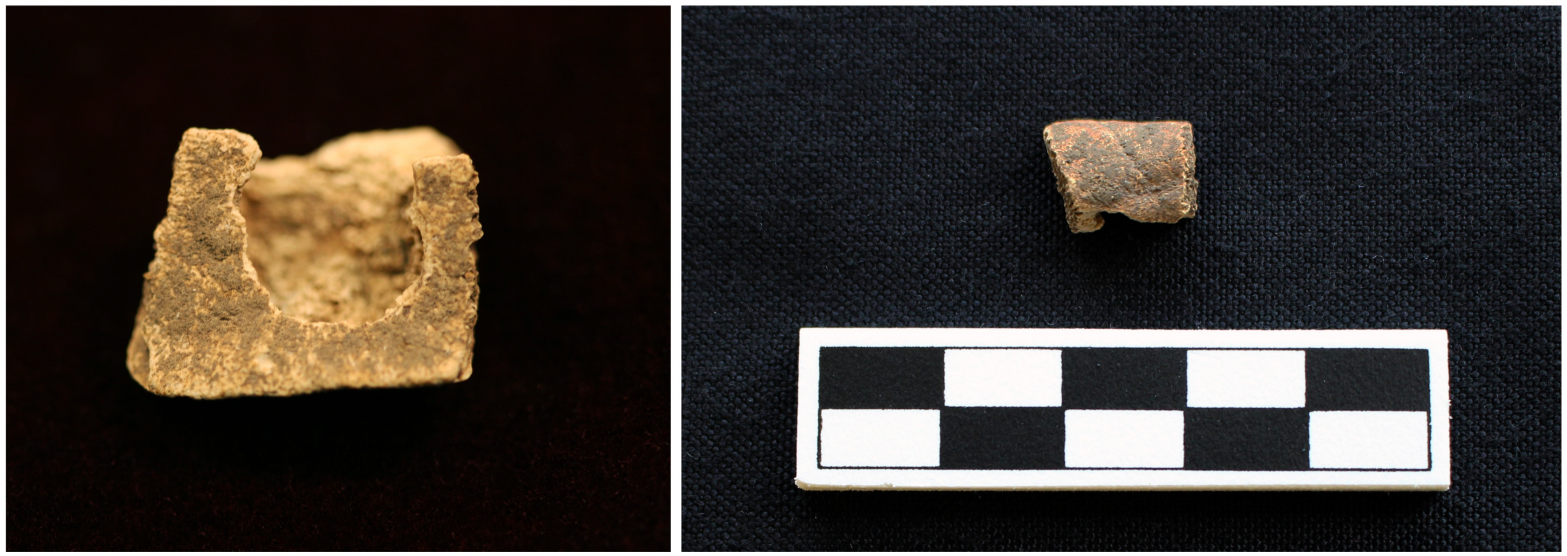
Partially drilled box turtle marginal recovered from Feature 55 at Fewkes. The exterior portion of the shell is shown on the left and the interior is shown on the right. The arrow shows a potential second drilled hole. Image on right is courtesy of the Tennessee Department of Transportation, Nashville.

The Fewkes site yielded modified turtle remains, mostly adult specimens, from six cultural features. The largest of these, Feature 55, is located on the edge of the palisade wall that surrounded the community. The original excavations identified it as a possible borrow pit that was filled with “domestic refuse” [63], dating to ca. AD 1150 [63, 71]. The feature was probably associated with refuse and byproducts of food processing from Structure 21—a domestic wall trench structure, given the number of low-quality deer bones and evidence of marrow extraction [63]. One Eastern box turtle marginal appears to be partially drilled on the exterior, which has a circular shape (Table 3, Fig 9). Around the top right of the hole, a small notch (indicated by the arrow in Fig 9) with rounding and smoothing is present above, which could suggest that two holes were made into the marginal. The close proximity to the suture line could have caused the shell to fragment. While this could be suggestive of a rattle, sharp breaks or micro-striations within the hole are not present. Other box turtle elements, a femur and ischium, were also found in this feature. This specimen does not meet the carapace and plastron only trait (Trait 3). While the turtle remains in this feature meet two of the traits, turtle species (Trait 2) and modification (Trait 4) (Table 6), the experimental data and other remains traits suggest that this is not a fragment from a turtle shell rattle. Further, the location of the hole does not fit the typical placement of other drilled carapace holes, which are on the costal near the neurals (Figs 2 and 4).

**Table 6:** Presence/Absence of the five turtle shell rattle characteristics (object trait list) that occur at Fewkes and Castalian Springs, with residue staining added as a sixth trait [9].

Site	Feature/Context	Trait 1: Ceremonial, Ritual, Burial Association	Trait 2: Turtle Species Preference	Trait 3: Carapace and Plastron Representation	Trait 4: Modifications	Trait 5: Rattle Objects	Trait 6: Residue Staining
**Fewkes**	55		X		X^a^		
	184						
	549			X			X
	702		X	X			
	817		X	X	X^a^		
	818			X	X^b^		
	Test Unit 145		X	X	Possibly		
	Lot 233/235			X	Possibly		
**Castalian Springs**	4			X	X^a^		
	9		X		X^c^		
	23		X		X^c^		

^a^The modification on the turtle fragment refers to drilling.

^b^The modification on the turtle fragment refers to perforation.

^c^The modification on the turtle fragment refers to polishing.

**Fig 10:**
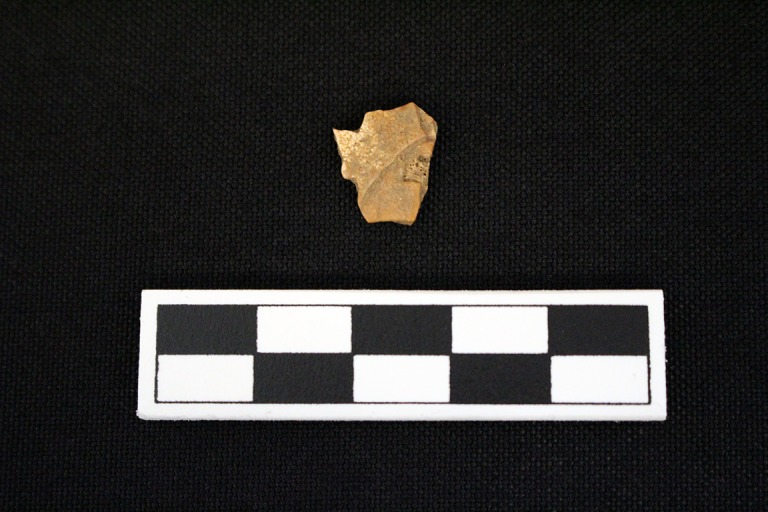
Turtle shell exhibiting sharp angles from Feature 549 at Fewkes. The fragment also has some smoothing on the sharply broken sides. Images courtesy of the Tennessee Department of Transportation, Nashville.

Feature 184 from Fewkes is identified as the matrix stratigraphically above two burials and Feature 185. Feature 185 is a hearth and possible feasting deposit, positioned on top of an adult male burial [63]. Features 184 and 185 date to approximately AD 1250–1450 [63, 71]. One costal fragment belonging to the order of turtles (Testudines) from Feature 184 shows evidence of green bone breakage (Table 3). It exhibits a v-shape that is congruent with breakage patterns seen in the experimental data (S39 Fig); however, the specimen does not have a drill indentation. The feature contains various turtle limb elements, such as a femur and humerus. Pharyngeal grinders of freshwater drum were also recovered from this feature, although no molariform teeth are reported. This fragment does not meet any of the expected trait list items (Table 6). While it is unlikely to be a rattle fragment, it cannot be completely ruled out, given its possible association with a burial area (Trait 1), that it could be box turtle (Trait 2), and the possible presence of rattle objects (Trait 5). Third, Feature 549 is potentially a hearth that is associated with Structure 12, another domestic wall-trench building. One turtle (Testudines) plastron fragment exhibits a sharp angle without a drill indentation (Table 3, Fig 10). The fragment also has some smoothing and polish that appears to be post-break. This fragment exhibits one trait: only carapace and plastron elements (Trait 3) represented in Feature 549 (Table 6). Thus, the fragment is unlikely to be the remains of a turtle shell rattle. One other small turtle (Testudines) shell fragment recovered from Feature 549 exhibited red ochre on the exterior (S40 Fig). This is intriguing and suggestive of ceremonial use, but as it does not meet any of the trait list items, it is not considered a rattle fragment.

**Fig 11:**
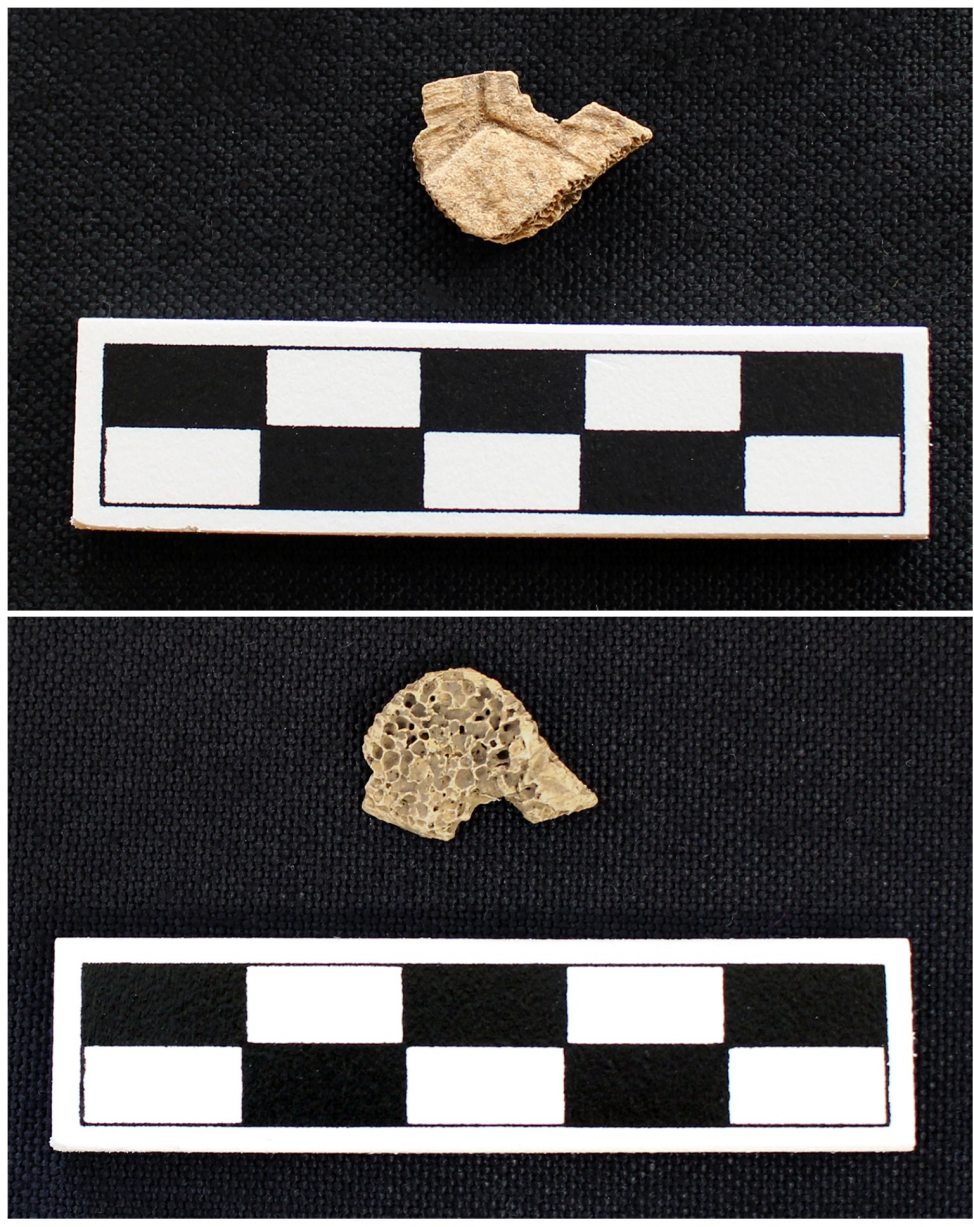
Turtle shell fragment showing possible evidence of drilling from Feature 702 at Fewkes. The hole does not reflect chert drilling characteristics. Images courtesy of the Tennessee Department of Transportation, Nashville.

While the function of Features 702 and 818 are not clear from the available information, they each yielded a modified turtle specimen. The Eastern box turtle fragment recovered from Feature 702 has a semi-circle shape showing possible evidence that it had been drilled (Traits 2, 3, and 4) (Table 3, Fig 11). In comparison to the experimental data, the sloping inward pattern from a chert drill is absent and the hole is quite irregular. However, the hole may have been made by another type of perforator tool. A freshwater drum pharyngeal fragment was present in the feature (possible Trait 5). Only carapace and plastron fragments were present in the feature (Trait 3) (Table 6). While the evidence does not unequivocally indicate that this is a fragment of a turtle shell rattle, it is suggestive.

Feature 818 yielded a softshell turtle costal fragment that has a puncture hole within a larger concavity (Table 3, S41 Fig). The indentation does not exhibit the characteristics of using a chert drill. The walls of the concavity have a narrow oval-shape that is not similar to the experimental data, at least with a full drilling motion. Further, deep vertical striations are present in the side of the concavity that likely suggests a puncturing method. This fragment has two traits: carapace and plastron representation-only (Trait 3) and modification (Trait 4) (Table 6). Considering the low number of identified traits and the identification as a softshell turtle, this modified fragment is unlikely to be part of a rattle.

**Fig 12:**
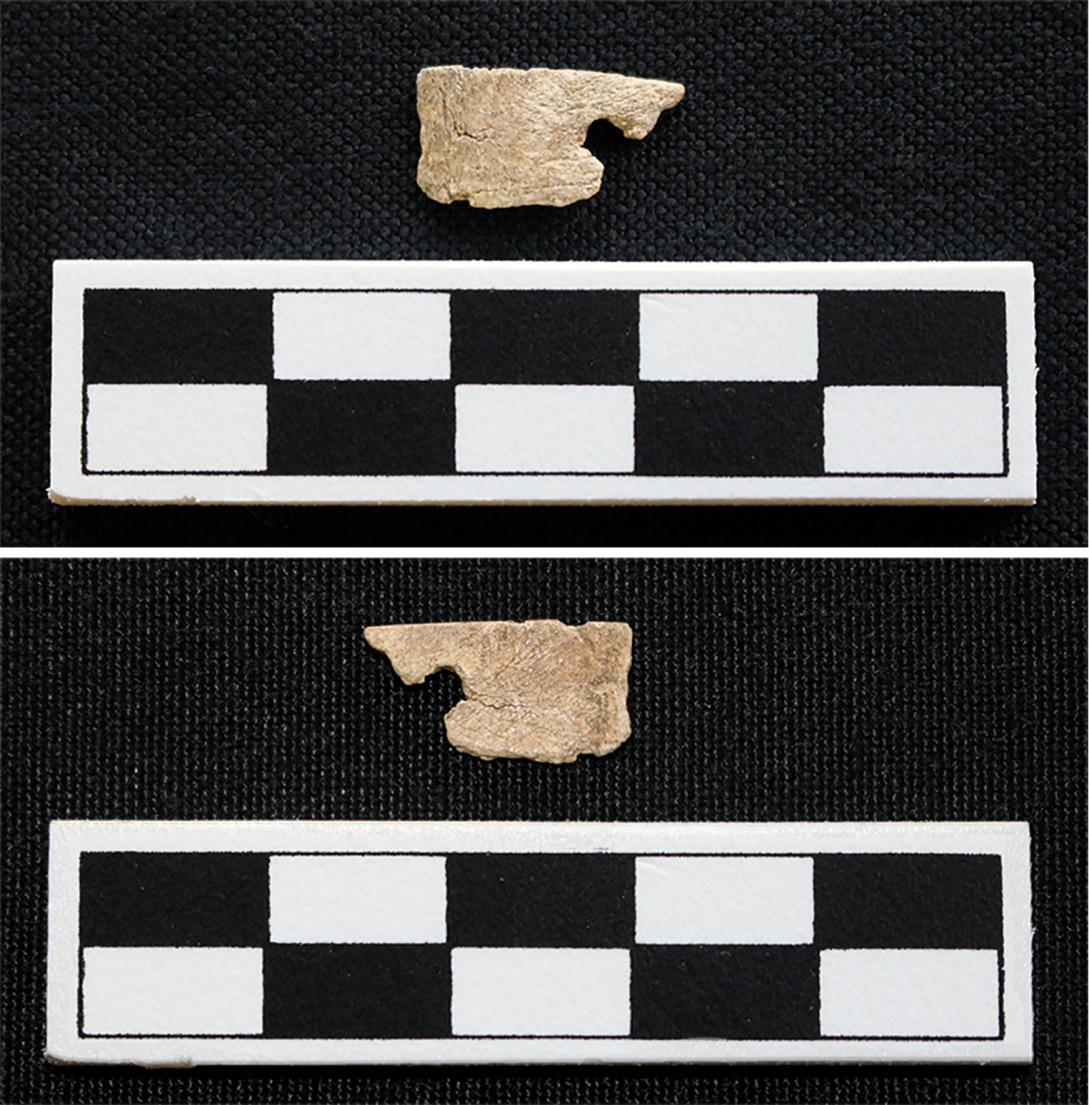
Drilled Eastern box turtle fragment recovered from Feature 817 at Fewkes. The hole is on a costal fragment, likely towards the neurals, and exhibits a mostly circular shape. Images courtesy of the Tennessee Department of Transportation, Nashville.

Feature 817 is a large, circular, shallow, concave-shaped pit [63]. Feature 817 yielded one Eastern box turtle carapace fragment, which has a concave shape (Fig 12). The placement of the hole on a costal is more in line with archaeological examples of turtle shell rattles (Fig 4A and 4E). There are also some possible striations around the hole; however, since the specimen has evidence of weathering, it is difficult to fully assess for striations. The characteristics of the hole suggest that it was drilled. This fragment also has three of the traits: species preference (Trait 2), carapace and plastron fragments were the only elements present in the feature (Trait 3), and modification (Trait 4) (Table 6). This fragment is the best evidence of a turtle shell rattle fragment from Fewkes.

**Fig 13:**
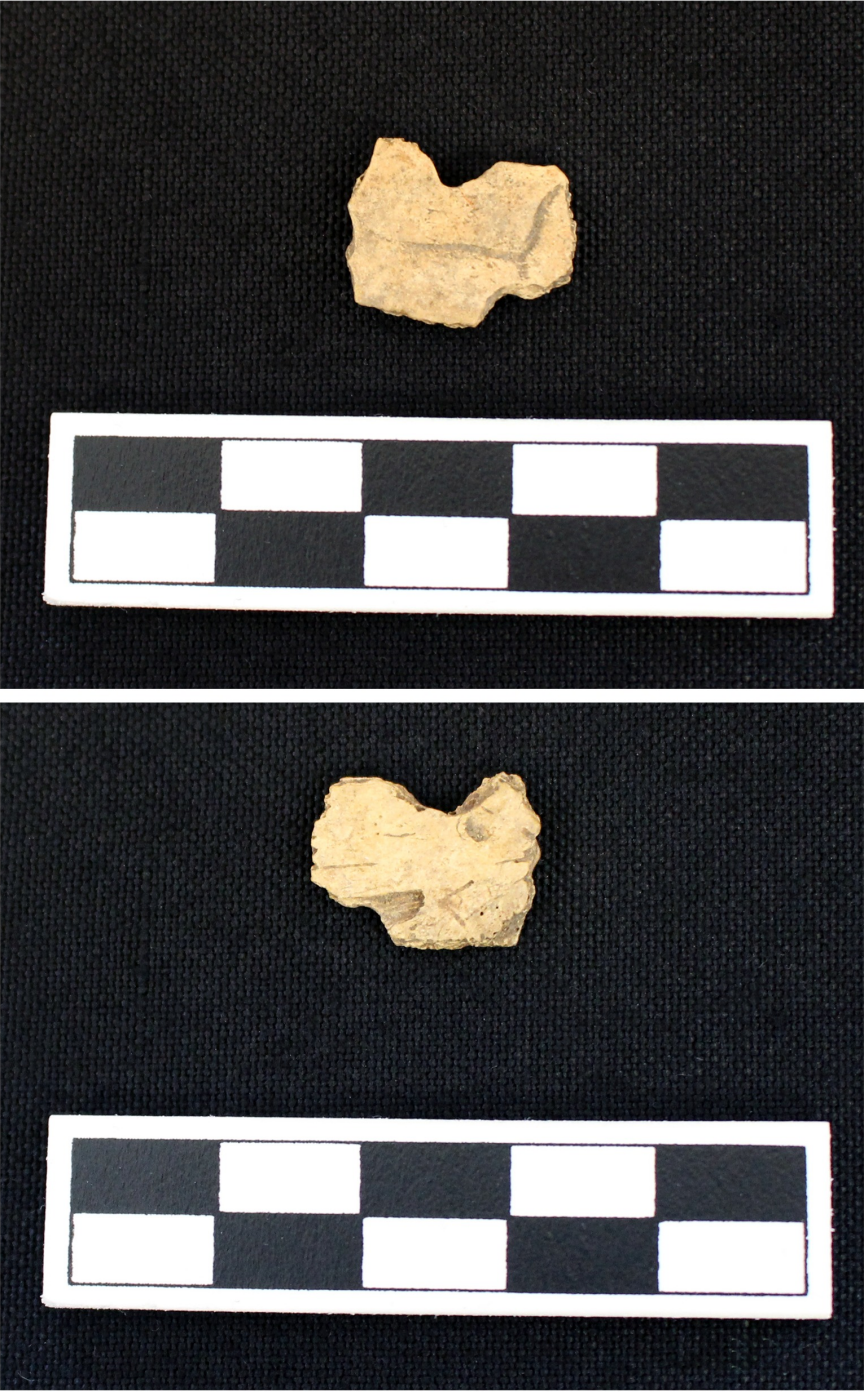
Rounded indentations in Eastern box turtle fragment recovered from Test Unit 145 at Fewkes. Images courtesy of the Tennessee Department of Transportation, Nashville.

**Fig 14:**
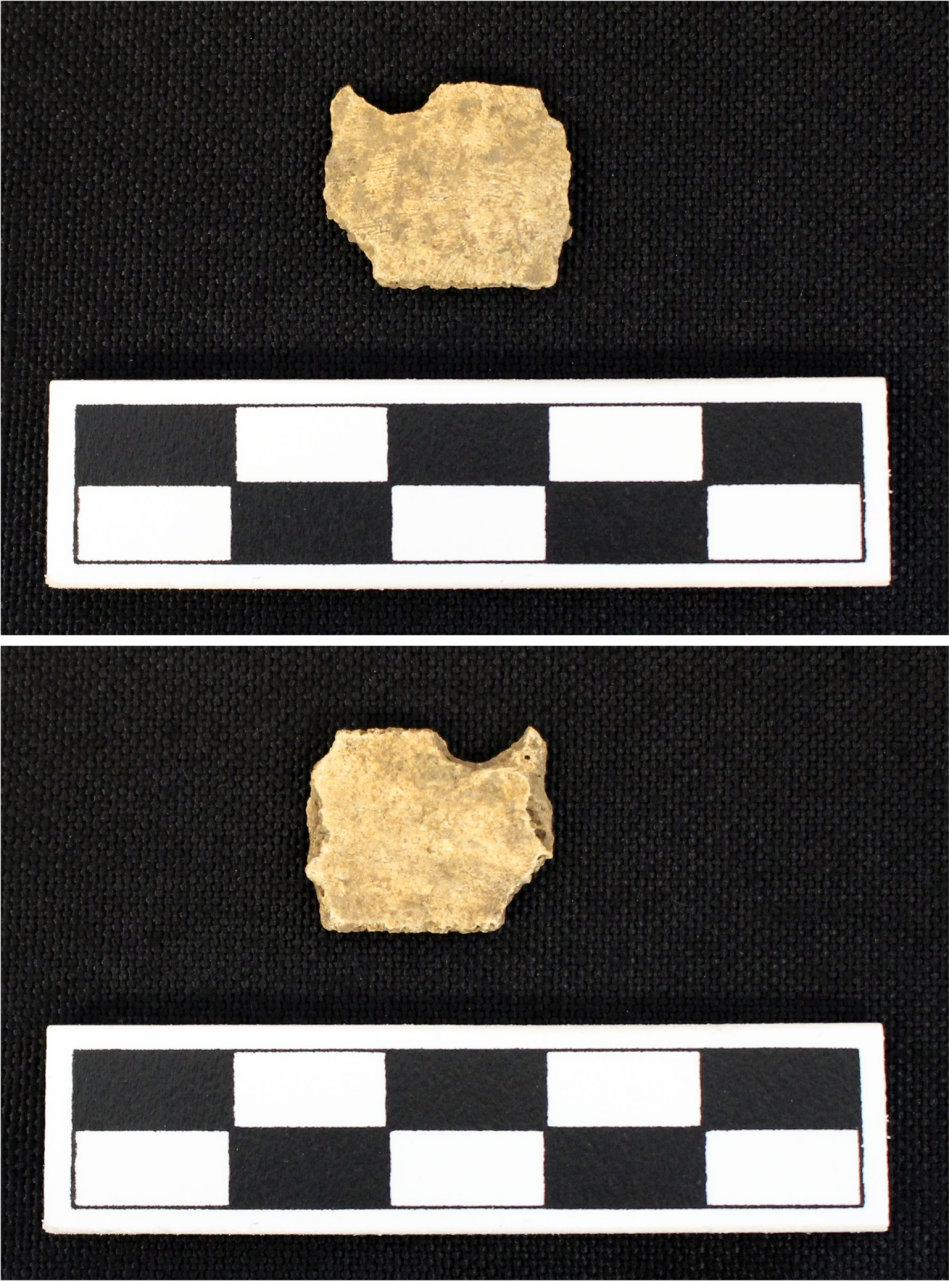
Hole in turtle fragment that shows signs of possible modification recovered from Lot 233/235 at Fewkes. The interior smoothing of the partial hole suggests possible modification. Images courtesy of the Tennessee Department of Transportation, Nashville.

Finally, two turtle specimens from general excavation contexts, Test Unit 145 and Lot 233/235 (Block A, Level 13/3), contain semi-circular holes. The turtle specimen recovered from Test Unit 145 is identified as Eastern box turtle. This specimen has an irregular hole and does not exhibit micro-striations within the hole or sharp-breaks around the edges. The evidence is not clear whether this fragment was modified. However, if it was modified, then it was likely punctured, given the rough texture and irregular shape (Fig 13). This fragment exhibited two traits: species preference (Trait 2) and skeletal element representation (Trait 3) (Table 6). The turtle specimen from Lot 233/235 is identified as belonging to the order of turtles (Testudines). It contains a hole and a mostly rough interior texture that does not indicate intentional micro-striations (Fig 14). Preservation does not allow for a clear assessment of the micro-striations. The partial hole is mostly smooth, but the straighter edge also suggests that the shell was broken around the hole. Thus, it is difficult to fully determine if the costal fragment was drilled. This fragment only has one trait—skeletal element representation (Trait 3) (Table 6).

### Castalian Springs

**Fig 15:**
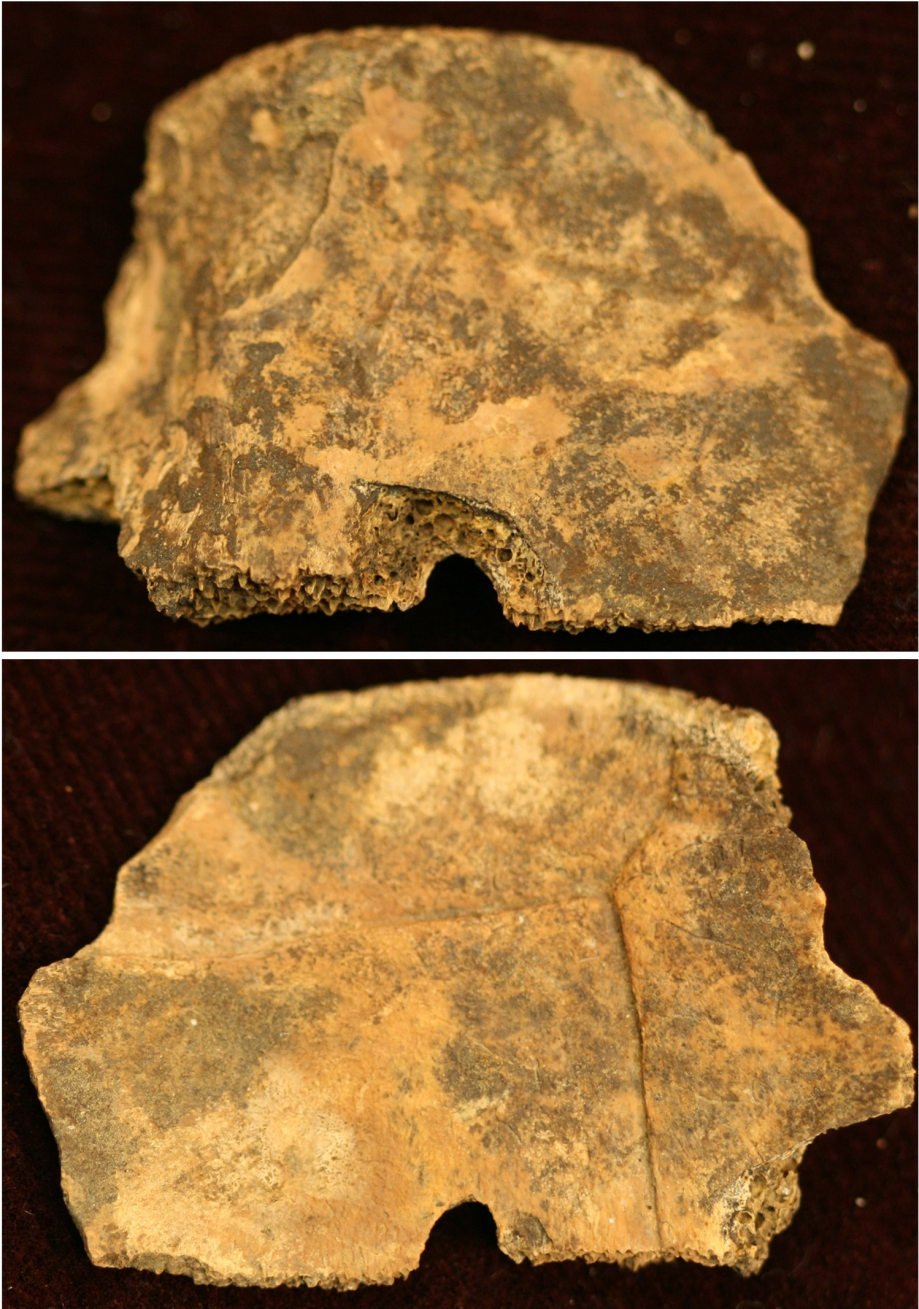
Drilled pond slider turtle recovered from Feature 4 at Castalian Springs. The interior side of the shell is shown at the top and the exterior of the shell is shown on the bottom.

Castalian Springs yielded modified turtle remains from three cultural features. Feature 4 is located on the south side of the plaza area, and is described as a large pit that was rapidly filled with midden. This feature yielded seven modified turtle shell fragments, identified as pond slider (n = 1) and Eastern box turtle (n = 6) [64] (Beahm 2013:64). A pond slider fragment yielded a drilling indentation that exhibits the sloping inward motion consistent with the use of a chert drill bit (Table 5, Fig 15). It had just broken through the exterior surface, when drilling was halted. The other half of the circle is missing; however, it is unclear whether this was during the drilling process or later. The break is old because older bone is typically darker and the pores of the bone are obscured. In fresh breaks, the pores of the bone are very distinct. This fragment has also been subjected to weathering, so it is not possible to evaluate the micro-striation characteristic. This specimen has two traits: skeletal element representation (Trait 3) and modification (Trait 4) (Table 6). While this fragment could be the remains of a rattle, the evidence, particularly the trait list, suggests that it is not, although it cannot be completely ruled out. In addition, six Eastern box turtle fragments exhibit polish of which five of them are polished on the interior and exterior (Trait 4) (S42 Fig).

Other turtle remains include those two features (9 and 23) that are part of Wall Trench Structure 1. Feature 9 is the large central posthole of Structure 1, which is an elite-affiliated structure. This feature yielded one modified (ground and polished) Eastern box turtle specimen (Traits 1, 2, 3, and 4). Only carapace and plastron fragments were recovered from Structure 1. Feature 23, which was associated with the second interior-most west wall trench of Structure 1 [64], yielded one modified (polished) Eastern box turtle shell fragment (Traits 1, 2, 3, and 4). Finally, William Edward Myer who excavated the burial mound at the site in the early 1890s and 1916–1917 does not mention turtle rattles in his account of burial-associated objects [75]. While the evidence for the crafting and use of turtle shell rattles is not unequivocal, it is possible turtle shell rattles were used at Castalian Springs. Additionally, the function of the Feature 4 modified fragment remains intriguing and suggests other taxa may have been used for rattle manufacture.

### Archaeofaunal discussion

The experimental phase of this project showed that crafting modern adult turtle shells into rattles can result in specific breakage patterns. Using the breakage pattern data and the object trait list, we assessed the Fewkes and Castalian Springs turtle specimens for evidence of turtle shell rattle manufacture. The lack of Traits 1 (context) and 5 (rattle objects), decreases the probability that the broken turtle shell fragments identified at Fewkes were part of rattles (Table 6). The turtle fragments from Castalian Springs are intriguing, but again the entire trait list is not met, thus we cannot say with certainty that turtle shell rattles were made and used here. Residue and staining does occur on specimens from Fewkes but they are not present on the specimens from Castalian Springs. The residue is not a clear indicator, as it has not been noted in any archaeological correlates. Rattle objects have not been recorded from either site, which could be a sampling bias because the objects may have been discarded during screening or, in the case of pebbles, put in a general non-modified lithic category. Additionally, freshwater drum molariform teeth and pharyngeal grinders are present at Fewkes, which would provide another possible material for rattle objects in addition to pebbles and seeds that are more readily accessible. Castalian Springs does not show conclusive evidence to support turtle shell rattle production and use. However, the drilled pond slider fragment may be evidence of rattle production but with a turtle species other than *T*. *carolina*. The best evidence for the crafting of turtle shell rattles from either site is from Feature 817 at Fewkes.

## Discussion

The correct functional identification of turtle shell fragments, like many other faunal specimens, suffers from the problem of equifinality [9, 76]. Through experimental archaeology, we reconstructed some possible avenues for turtle shell rattle construction and created some fragmented pieces of turtle shell from failed attempts during the process. Fragmentary turtle specimens may represent food waste, tools, instruments or a combination of these. Thus, the object trait list [9] and experimental data can help to distinguish between some uses of turtle remains.

The experimental process revealed some potential diagnostic characteristics of production methods. We used these indicators—sharp-angled breaks with drill indentations, micro-striations in drill hole, concave oval and circular shape of drill hole, and striations around the exterior of the drill hole—to evaluate turtle elements from two archaeofaunal assemblages in Middle Tennessee. Some of the turtle specimens had one or more characteristics from the object trait list. Some of the elements may have been failed attempts at making a rattle during the manufacturing process, such as Feature 184 from Fewkes, although the evidence suggested that Feature 184 was likely not associated with a rattle. The evidence suggests that many of the modified turtle shell fragments are likely not remains from turtle shell rattle manufacture, except for the specimen from Feature 817 at Fewkes (and potentially the one from Feature 4 at Castalian Springs).

Turtle shell rattles are known from archaeological sites in East Tennessee [8], a number of which are curated at the McClung Museum of Natural History and Culture. Although rattles are present in Middle Tennessee during the Archaic [8, 31], they may not have been used by the people living in the area during the Mississippian period. The divergence of Middle and East Tennessee in abundance of recovered rattles suggests that the peoples in these areas were culturally distinct. This distinction is also made by Lewis [77] in regards to geography.

We offer five possible reasons for the lack of conclusive evidence of turtle shell rattles in Mississippian sites in Middle Tennessee. First, the people of Fewkes and Castalian Springs were not producing turtle shell rattles on site. Second, turtle rattles only occur in mortuary or ceremonial contexts, which mostly remain un-excavated at the two sites (at least for the more recent excavations), although William Myer excavated burials at Castalian Springs in the early 1890s and in 1916–1917 [72, 75]. Third, rattles could be associated with a particular status, of which no one had achieved at these two sites, or that the rattles represented a meaning that was not significant to these communities. Fourth, people did not construct new rattles on a regular basis, and instead transferred them to others as heirloom items. However, it is expected that some rattles would have been identified from burial contexts. Finally, people may not have been using turtle shell rattles at all, or only minimally, in Middle Tennessee during the Mississippian period.

For future research, it would be beneficial to find and test new methods of rattle construction. In the present study, we identified one chaîne opératoire for construction of a single rattle type. Depending on turtle species and geographic region, rattles are constructed in many different ways [8–9]. The different construction types and processes could lead to different modification characteristics and breakage patterns. For those interested in rattle construction in areas outside of the southeastern United States, we urge them to conduct their own experimental tests of construction methods to identify a broader range of variation of modifications and breakage patterns from manufacturing. One addition could be placing a few rattles on a hide then attaching to the leg or using a handle. New ways to prepare a deceased turtle should be tested such as putting the turtle in an ant pile. Additional tests and new strategies using river cane (*Arundinaria* sp.) should be attempted to be sure that it is not a viable method; thus, it would be necessary to work with someone who has those skills. More shaking tests should be run to determine the longer effects of rattle objects. This could be done by working with Indigenous peoples who make and use rattles today. While many questions and tests remain, this study shows the promise of better distinguishing between turtle fragments as food refuse, crafting byproducts, or fragments of musical instruments.

## Conclusion

Turtle shells were used as musical instruments for thousands of years around the world by the Aztec [78], Indigenous North Americans, and the peoples of Amazonia [79], Neolithic China [80], Greece, and around the Aegean [81]. Turtles play an important role in Native North American cosmology, particularly in creation stories that the world formed on the turtle’s back. Thus, the use of turtles for musical instruments interjects powerful symbology into ceremonies and rituals. Turtle shell rattles empower dancers (or shakers) and heighten the spiritual energy of a ceremony or ritual. Turtle shell rattles have been an important part of North American musical traditions, dating back to at least the Archaic period, although turtle shell artifacts have been discovered from the Paleoindian period [82]. However, because of their organic nature turtle shell musical instruments do not always preserve intact. Additionally, archaeological turtle remains are typically relegated to subsistence remains without full consideration of other potential functions. In this study, we highlighted some key characteristics from rattle construction that could help to identify rattle remains from the archaeological record. The experimental data and object trait list can be used together to aid in not only distinguishing food refuse from rattles, but also between rattles and other modified turtle artifacts. Future experimental research, using other rattle construction methods/techniques, can be compared to our experimental data on striations, drilling characteristics, and measurements. These data can be compared to other known and analyzed archaeological turtle shell rattles. We suggest that these criteria be used with previously analyzed faunal assemblages to tease out and identify rattle remains.

This study highlights the use of multiple lines of evidence through the object trait list and experimental archaeology. For example, without knowing what drill marks specifically look like, it can be easy to misclassify perforated or punctured turtle remains as rattles, given that the fragments meet several traits. Thus, experimental archaeology, particularly related to musical instruments, provides additional insights into the instrument-making process and the anticipated traits of instrument remains in the archaeological record.

North American Indigenous groups’ beliefs about turtles provide a greater understanding of why turtles are incorporated into ceremonies and dances and why they are used to keep rhythm, which provide a basis for spiritual energy and experience. However, it appears from the available data that turtle shell rattles were not widely incorporated into music in Middle Tennessee during the Mississippian period, although they are known from archaeological contexts of the preceding Archaic period and from contemporaneous sites in East Tennessee. Ancient music culture is diverse across prehistoric North America and it behooves us as archaeologists to find ways to “see” this important auditory aspect of past cultures.

## Supporting information

S1 Text Information on materials and methods of the experimental study and a step-by-step description of the drilling process on turtle shell (also referred to as the main drill description) and the ten drilled holes (Drill Holes 1–10). (DOCX)

S1 Table Description of revolution drilling sequence for Carapace B (Drill Holes 5 and 6) in the experimental study. (DOCX)

S1 Fig Bow and chert drill in action on Drill Hole 4. (TIF)

S2 Fig Close up of river cane tips used for drilling the turtle shell in the experiment. (TIF)

S3 Fig The beginning of the drilling process on the carapace of Drill Hole 4. (TIF)

S4 Fig Circular-shaped hole from changing the drill angle on Drill Hole 4. (TIF)

S5 Fig Before using river cane to polish out excess bone on Drill Hole 4. (TIF)

S6 Fig After using river cane to polish out excess bone on Drill Hole 4. (TIF)

S7 Fig Oval-shape in the drill opening of Drill Hole 4. (TIF)

S8 Fig Drill Hole 4 Carapace. The lighter (or whiter) parts of the turtle shell are the bony plates. The darker (or browner) parts of the turtle shell are the epidermal scutes.
(TIF)

S9 Fig Experimental marginal about half way through the drilling process (Drill Hole 1). (TIF)

S10 Fig Experimental marginal (Drill Hole 1) after the shell began to break. (TIF)

S11 Fig Experimental costal (Drill Hole 3) (before any drilling had occurred). (TIF)

S12 Fig Experimental costal (Drill Hole 3) after the shell split along the suture. (TIF)

S13 Fig Experimental costal (Drill Hole 3) with break indentation. (TIF)

S14 Fig Breakage patterns: sharp angle. (TIF)

S15 Fig Drill indentation on Drill Hole 3. (TIF)

S16 Fig River pebbles collected from Stones River in Murfreesboro, Tennessee. (TIF)

S17 Fig Close up of river pebble surface. (TIF)

S18 Fig Drill Hole 1. Exterior of turtle shell (from drilling side).
(TIF)

S19 Fig Drill Hole 1. Interior of turtle shell.
(TIF)

S20 Fig Drill Hole 3. Exterior of turtle shell (drilling side).
(TIF)

S21 Fig Drill Hole 3. Interior of turtle shell (opposite of drilling side).
(TIF)

S22 Fig Drill Hole 2. Exterior of turtle shell (drilling side).
(TIF)

S23 Fig Drill Hole 2. Interior of turtle shell (opposite of drilling side).
(TIF)

S24 Fig Close up of sharp-angled breakage pattern. (TIF)

S25 Fig Drill Hole 4. Exterior of turtle shell (drilling side).
(TIF)

S26 Fig Drill Hole 4. Interior of turtle shell (opposite of drilling side).
(TIF)

S27 Fig Drill Hole 10. Exterior of turtle shell (drilling side).
(TIF)

S28 Fig Drill Hole 10. Interior of turtle shell (opposite of drilling side).
(TIF)

S29 Fig Drill Hole 6. Exterior of turtle shell (drilling side).
(TIF)

S30 Fig Drill Hole 6. Interior of turtle shell (opposite of drilling side).
(TIF)

S31 Fig Drill Hole 5. Exterior of turtle shell (drilling side).
(TIF)

S32 Fig Drill Hole 5. Interior of turtle shell (opposite of drilling side).
(TIF)

S33 Fig Interior portion of Drill Holes 5 and 6 carapace after shaking with river pebbles inside the rattle. (TIF)

S34 Fig SEM micrograph showing micro-striations in the interior of Drill Hole 2. The exterior of the turtle shell can be seen on the very right side of the image. Image was captured on a Tescan Vega 3 SEM at 600x magnification. Bar = 100 μm.
(TIF)

S35 Fig SEM micrograph showing micro-striations in the interior of Drill Hole 2. Image was taken close to the upper edge of the drill hole. Image was captured on a Tescan Vega 3 SEM at 2,190x magnification. Bar = 20 μm.
(TIF)

S36 Fig SEM micrograph showing micro-striations in the interior of Drill Hole 2. Image was taken approximately 0.47 mm from the upper edge of the drill hole. Image was captured on a Tescan Vega 3 SEM at 1,000x magnification. Bar = 50 μm.
(TIF)

S37 Fig SEM micrograph showing micro-striations in the interior of Drill Hole 2. Image was taken approximately 1.89 mm from the upper edge of the drill hole. Image was captured on a Tescan Vega 3 SEM at 1,000x magnification. Bar = 50 μm.
(TIF)

S38 Fig SEM micrograph showing micro-striations in the interior of Drill Hole 2 and unintentional modification close to drill hole edge. The image shows how the drill slipped during drilling or while starting the drilling process. Image was captured on a Tescan Vega 3 SEM at 189x magnification. Bar = 200 μm.
(TIF)

S39 Fig V-shaped break and cancellous portion of the shell recovered from Feature 184 at Fewkes. (TIF)

S40 Fig Turtle shell exhibiting sharp angles on the left and stained with red ochre on the right from Feature 549 at Fewkes. Image courtesy of the Tennessee Department of Transportation, Nashville.
(TIF)

S41 Fig Perforated soft-shelled turtle fragment recovered from Feature 818 at Fewkes. (TIF)

S42 Fig Polished turtle shell recovered from Feature 4 at Castalian Springs. (TIF)

S1 Video Drilling the carapace on Drill Holes 5 and 6. This video shows short segments of the drilling process during the experimental archaeology part of the project.
(MP4)

S1 3D Model Three-dimensional model of the adult turtle shell carapace used in the experimental study. The model contains Drill Holes 5 and 6.
(OBJ)
