# Supplementary material for: An experimental study of turtle shell rattle production and the implications for archaeofaunal assemblages
Source: PLoS One. 2018 Aug 2;13(8):e0201472. doi: 10.1371/journal.pone.0201472 (PMC6072095; doi:10.1371/journal.pone.0201472)
Supplement: S1 Text — (DOCX) [file pone.0201472.s001.docx]

**Materials and methods for the experimental study**

**Collection of materials**

**Turtle shells**

A complete previously-dead modern specimen of Eastern box turtle (*Terrapene carolina*) was collected along US Highway 231 South approximately ten miles north of Fayetteville, Tennessee on October 8, 2011. The specimen only had ants on it, but the carapace had been partially-cracked from being hit by a car. The specimen, referred to as Drill Hole 4 (the carapace) and Drill Holes 7–10 (plastron) had some residual soft tissues. The specimen was cleaned before any experimental work was conducted by soaking it in a solution of papain (papaya enzyme) and water, which was changed every two days for a total of eight days. Papain is well-known in zooarchaeological circles as it breaks down meat tissues quickly [1]. Biz brand degreaser detergent, which was diluted with water, was used to remove any remaining tissue. The turtle remains sat in a mesh container for approximately four days in the solution. Finally, the carapace and plastron were soaked in a diluted hydrogen peroxide solution for approximately three hours to kill any remaining bacteria. A second box turtle carapace (Drill Holes 5 and 6) was donated to us by a colleague, who found the shell in the woods in Murfreesboro, Tennessee around 2009. This specimen had been exposed to the elements and was naturally clean, thus no treatments were necessary. It is possible that the treatments on some of the samples may have changed the density of the bone, which may have increased the brittleness of the bone [2-3].

**River Cane**

For the current project, river cane was obtained from the banks of the Cumberland River in Nashville, Tennessee. Different sizes of both dry and green river cane were collected and used in the first attempt to drill a hole into the turtle shell.

**Bow and chert drill**

The tool was constructed by Mark Norton, formerly of the Tennessee Division of Archaeology in Nashville, Tennessee. Details of the bow and chert drill are in the main article.

**Documentation of the experimental study**

The experimental study was documented using a Canon EOS Digital Rebel XTi and a Panasonic SDR-S7 video camera. The entire process of drilling and rattle creation was recorded and reviewed. Digital photos were taken throughout the process, including after every few revolutions of the chert drill, breakage patterns, and other noticeable signs of wear. The drilled holes were measured at the widest sections at the end of drilling. Measurements were conducted on an Olympus SZX7 microscope, with an Olympus ACH 1.25x lens and Olympus SC100 mounted camera. The camera and imaging/measurement program was cellSens Standard 1.9, which also allows for calibration. The program allows for a live view and the ability for arbitrary line measurements. Finally, high-powered microscopy, specifically scanning electron microscopy (SEM), is particularly useful for identifying use wear on bone and stone tools. Thus, Drill Holes 1–3 were imaged after drilling was complete using the Tescan Vega 3 SEM in the Franceschi Microscopy and Imaging Center at Washington State University.

**Turtle shell rattle production**

**Drilling the turtle shell**

We begin by describing what happens throughout the drilling process, which will be referred to as the main drill description in the paper (see S1 Video for a sample of the drilling process). At the beginning of the drilling process, the walls of the perforation formed a concave shape. The interior wall of the drill cavity had a rough texture (S3 Fig). As the drilling process continued, an oval shape formed when the drill was maintained in the same position (S7 Fig). When the original angle of the drill was moved approximately 90 degrees, the oval shape slowly transitioned into a circular shape (S4 Fig). As the drill broke through into the interior of the shell, the hole maintained a concave shape and the texture was smooth within the cavity. River cane was used to polish and remove the excess bone from the hole (S5 Fig). This is shown in the before and after images (S6 Fig). As the shell was drilled from the interior of the shell, the opening expanded to about the same size as the starting drill side. The opening retained a slight notch in the interior of the drill opening (S7 Fig).

A total of four holes were drilled into various parts of a carapace (Drill Holes 1–4) (S8 Fig; also see Fig 2 for parts of a turtle shell). First, a marginal piece (Drill Hole 1) was drilled on the exterior (S9 and S18 Figs). The drill area was approximately in the top left corner. The piece exhibited the characteristics of the main drill description. The shell broke as soon as the drill pierced the interior of the shell. The pressure towards the suture line and outer edges were unable to endure the friction of the drill (S10 and S19 Figs). Second, a costal fragment (Drill Hole 2) also exhibited similar characteristics to the main drill description. The drill angle was not changed throughout the process so the final product yielded an oval area (S22 and S23 Figs). Third, another costal fragment (Drill Hole 3) consisted of three costals that were still linked together (S11 Fig). The costals exhibited a few different qualities due to the location of drilling. The location was in between two of the costals and close to the suture line. The first drill resulted in a break from a third costal, which left a drill indentation on one side (S12 Fig). The final drill on the costals resulted in the sutures splitting apart at the point of perforation. There was a slight break in the side of the drill opening. The largest measurement includes the slight break in the drill opening (S13 Fig; see S20 and S21 Figs for the final state). Fourth, a carapace (Drill Hole 4) resulted in a similar description (S8 Fig). This one was also drilled from the interior, which resulted in the suture being pulled apart. Drilling from the original side creates uneven areas on the interior, causing the drill to snag on these areas, and ultimately causing the shell to break apart (S25 and S26 Figs). Next, a fully-intact carapace (Drill Holes 5 and 6) and plastron (Drill Holes 7–10) make up the complete rattle. The second carapace (Drill Holes 5 and 6) was modeled after the Hiwassee Island archaeological examples [4-5] (Fig 8). The carapace was drilled in between the third neural and third costal on the left (Drill Hole 5) and right (Drill Hole 6) from the exterior side (Fig 8). Both drill openings maintained the structure of the main drill description (Fig 8 and S29–S32 Figs). The number of revolutions and description of the process are recounted in S1 Table. The drill moves approximately 90 degrees by hand, whereas the bow turns nearly a whole revolution. The drilling process took approximately ten minutes, excluding time spent on documentation. For the hyoplastron (Drill Hole 7) and hypoplastron (Drill Hole 8) hinge-tie, and the hyoplastron central (toward the midline) (Drill Hole 9) (see Fig 6A-C for Drill Holes 7–9), the openings were drilled from the interior but maintain the common characteristics of the main drill description. Last, the hypoplastron central (toward the midline) (Drill Hole 10) was drilled in the central area from the exterior (Fig 6D and S27 and S28 Figs), which was modeled after Hiwassee Island [5], Zebree [6-7], Apple Creek [8], and Ensworth [9] specimens (Fig 4) (see article for descriptions of the four sites). For the Ensworth rattle, the hypoplastron seemed to have a congruent opening instead of a concave opening. It is possible that another instrument was used to polish out the interior of the opening.

**References cited**

1. Ashie I, Sorensen T, Nielsen P. Effects of papain and a microbial enzyme on meat proteins and beef tenderness. *J Food Sci.* 2002;67:2138-42. 8.
2. Cumbaa SL. Osteological preparation techniques used by the Zooarchaeological Identification Centre. In: Faber DJ, editor. *Proceedings of the 1981 on Care and Maintenance of Natural History Collections*; Syllogeus. Ottawa (Canada): National Museum of Natural Sciences. 1983;44:29-35. 29.
3. Shelton SY, Buckley JS. Observations on enzyme preparation effects on skeletal material. *Collect Forum* 1990;6(2):76-81.
4. Brown AD. An exploration of turtle shell rattle manufacture in the Mississippian period [Senior Thesis]. Murfreesboro (TN): Middle Tennessee State University; 2011. Available from: https://doi.org10.13140/RG.2.1.2675.5921
5. Lewis TM, Kneberg M. *Hiwassee Island: An archaeological account of four Tennessee Indian peoples*. Knoxville (TN): University of Tennessee Press; 1970.
6. Morse DF. *Report of excavations at the Zebree site 1969*. Fayetteville (AR): Arkansas Archeological Research Report 4; 1975.
7. University of Michigan. Turtle carapace rattle. Ann Arbor (MI): University of Michigan Museum of Anthropology. 1970;1. Available from: http://quod.lib.umich.edu/a/anthro1ic/x-27521/27521.
8. Parmalee PW, Paloumpis AA, Wilson N. *Animals utilized by Woodland peoples occupying the Apple Creek site, Illinois*. Springfileld (IL): State of Illinois, Department of Registration and Education, Illinois State Museum; 1972.
9. Deter-Wolf A, Norris S, Wampler M, Tuschl J. *The Ensworth School project: Archaeological investigations at Site 40DV184, Davidson County, Tennessee*. Nashville (TN): TRC, Inc.; 2004.
