# Supplementary material for: An experimental study of turtle shell rattle production and the implications for archaeofaunal assemblages
Source: PLoS One. 2018 Aug 2;13(8):e0201472. doi: 10.1371/journal.pone.0201472 (PMC6072095; doi:10.1371/journal.pone.0201472)
Supplement: S1 Table — (DOCX) [file pone.0201472.s002.docx]

**S1 Table. Description of revolution sequence for Carapace B (Drill Holes 5 and 6).**

| **Drill Revolutions** | **Description** | **With Bow?** |
| --- | --- | --- |
| **0–125** | Drilling started by hand | N |
| **126–231** | Drill perforated into interior of shell | Y |
| **232–326** | Drill opening was concave and oval | Y |
| **327–621** | Drill opening was more circular | N |
