# Supplementary figures and images for: An experimental study of turtle shell rattle production and the implications for archaeofaunal assemblages
Source: PLoS One. 2018 Aug 2;13(8):e0201472. doi: 10.1371/journal.pone.0201472 (PMC6072095; doi:10.1371/journal.pone.0201472)

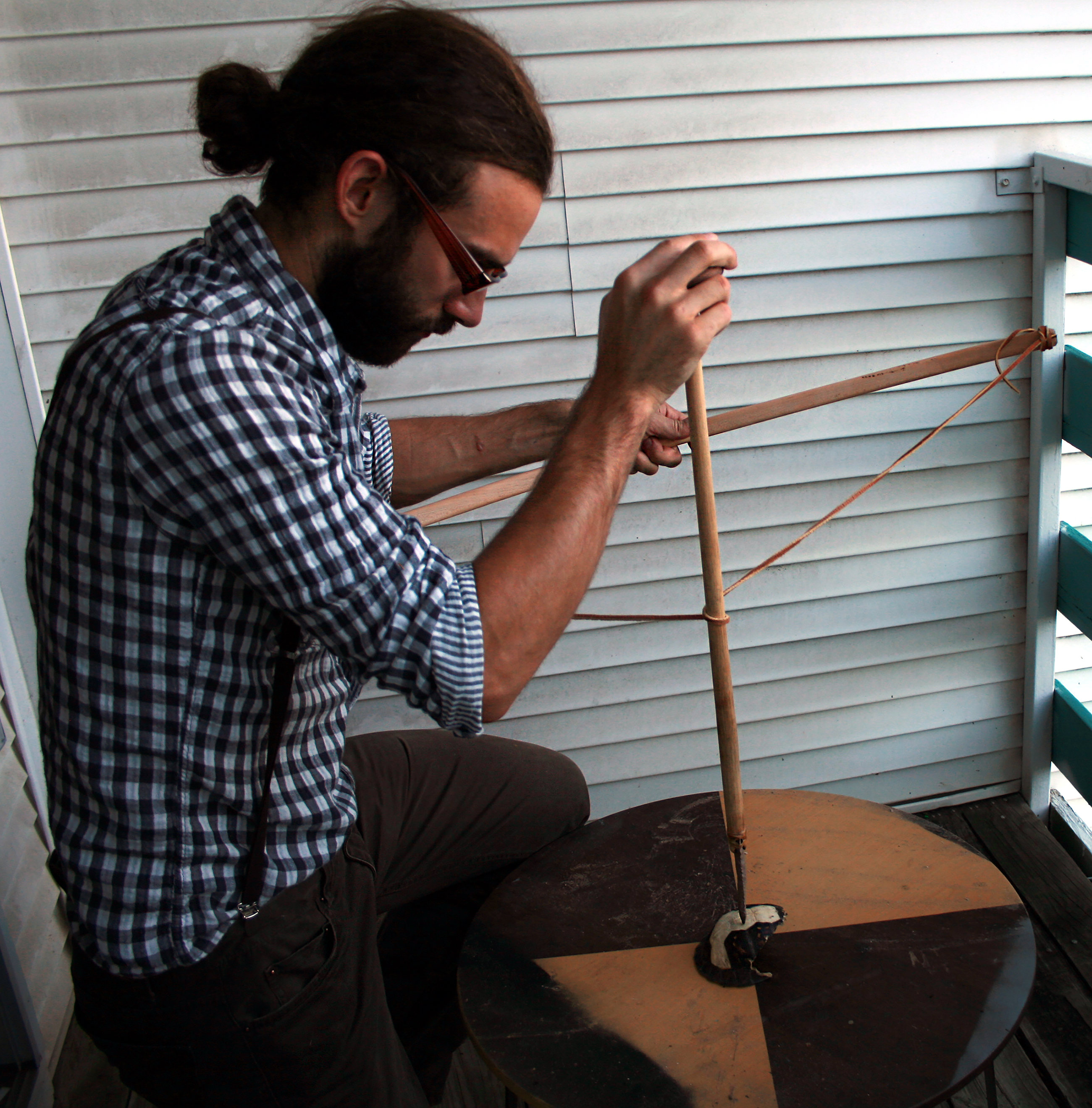

Supplement: S1 Fig — (TIF) [file pone.0201472.s003.tif]

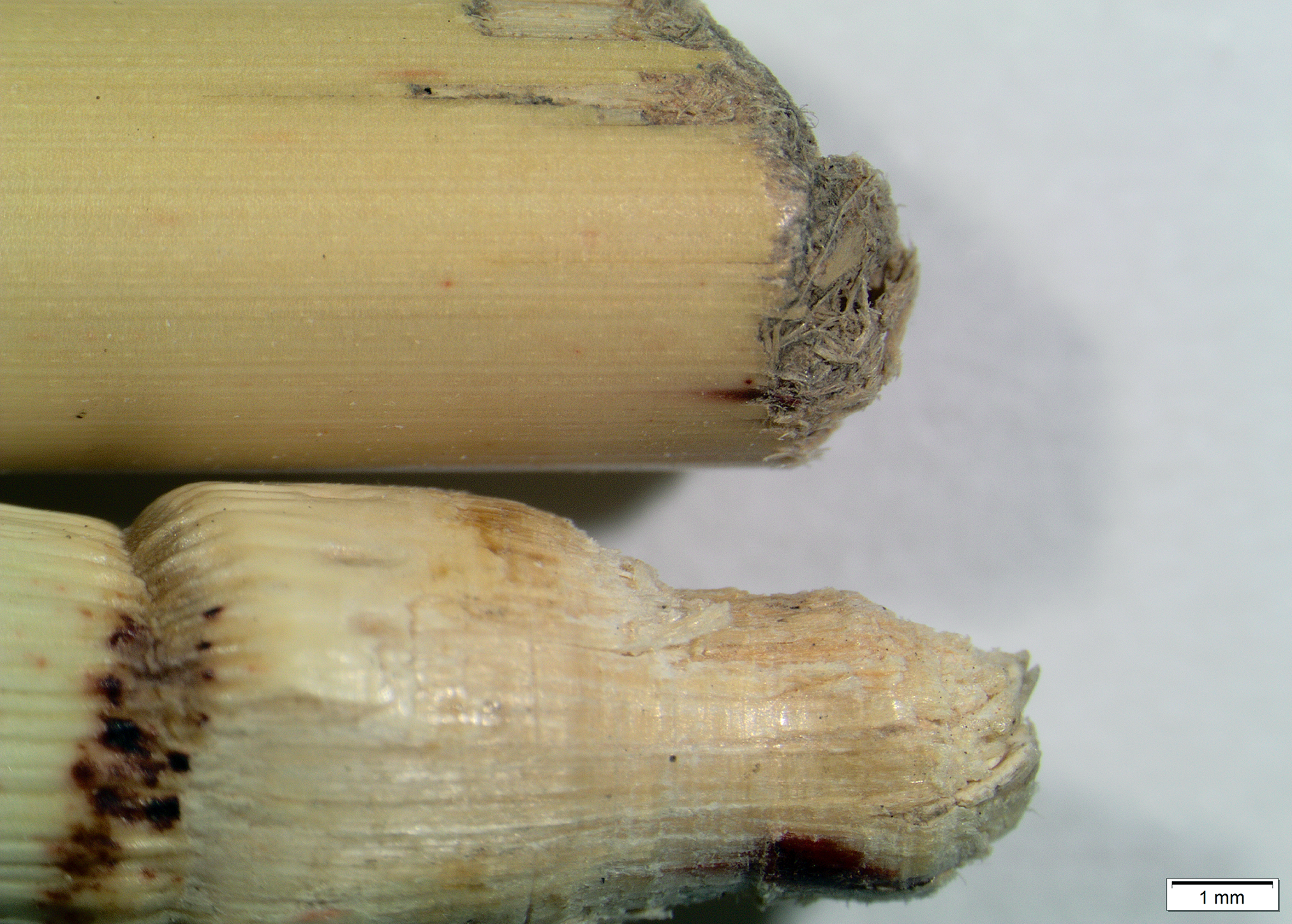

Supplement: S2 Fig — (TIF) [file pone.0201472.s004.tif]

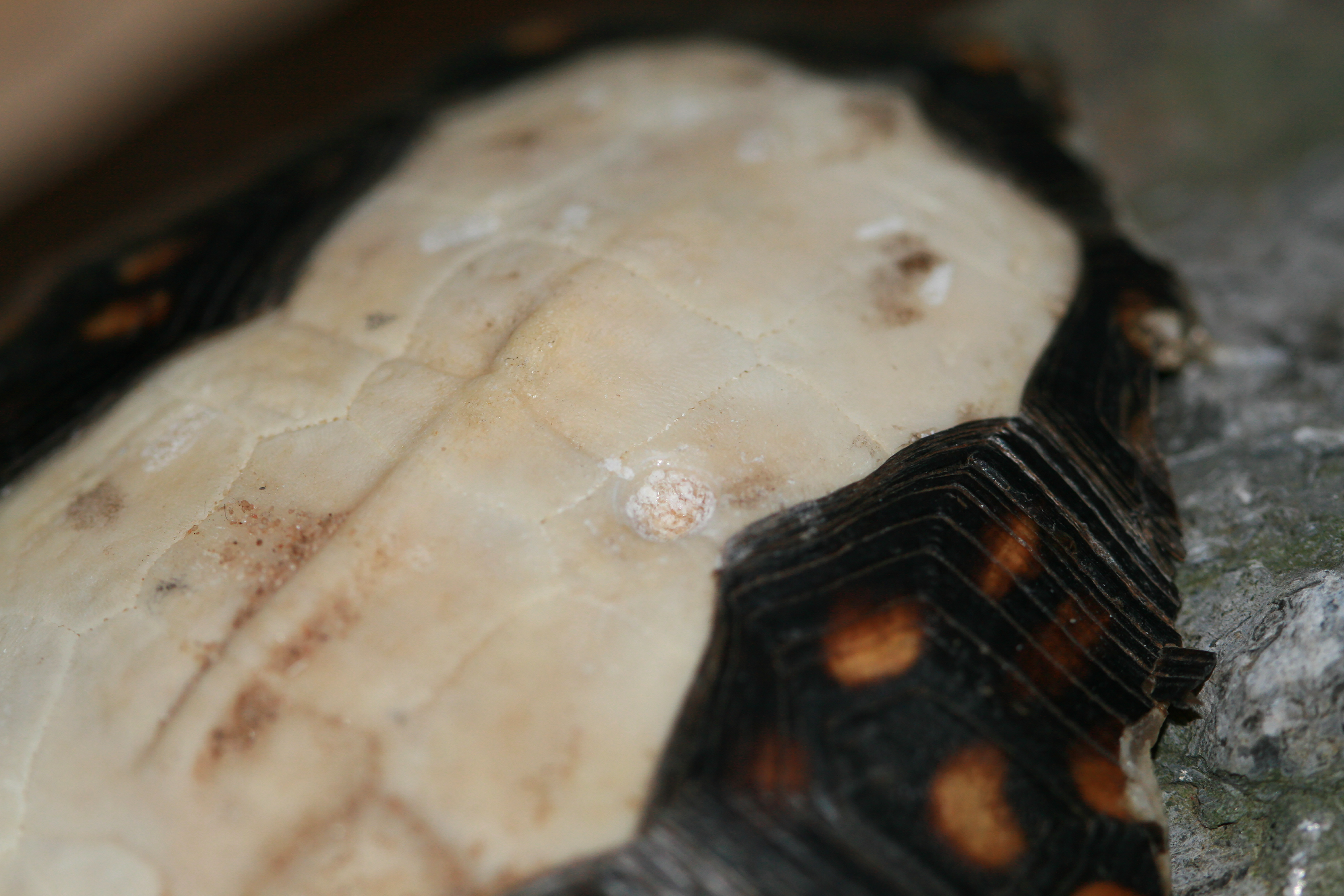

Supplement: S3 Fig — (TIF) [file pone.0201472.s005.tif]

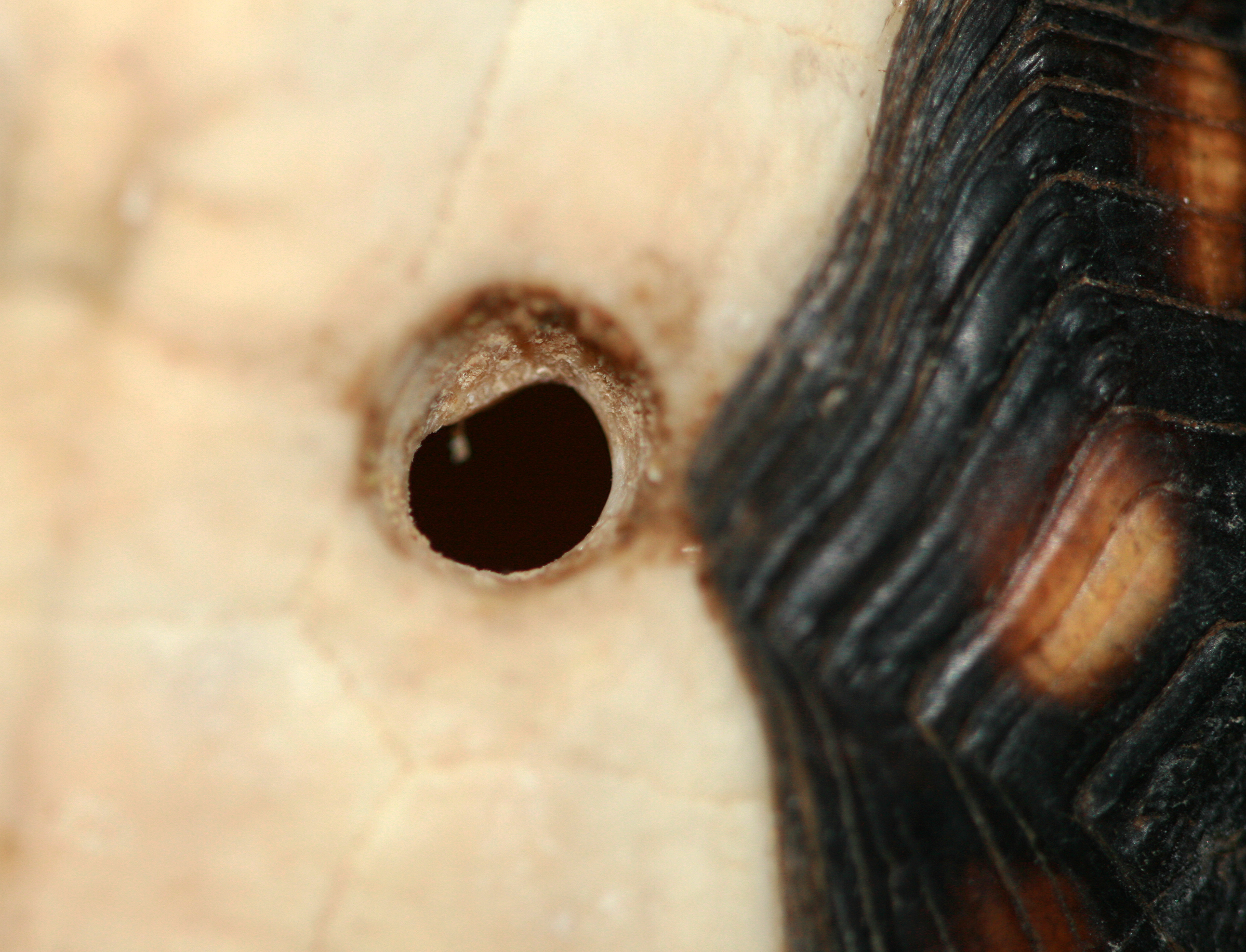

Supplement: S4 Fig — (TIF) [file pone.0201472.s006.tif]

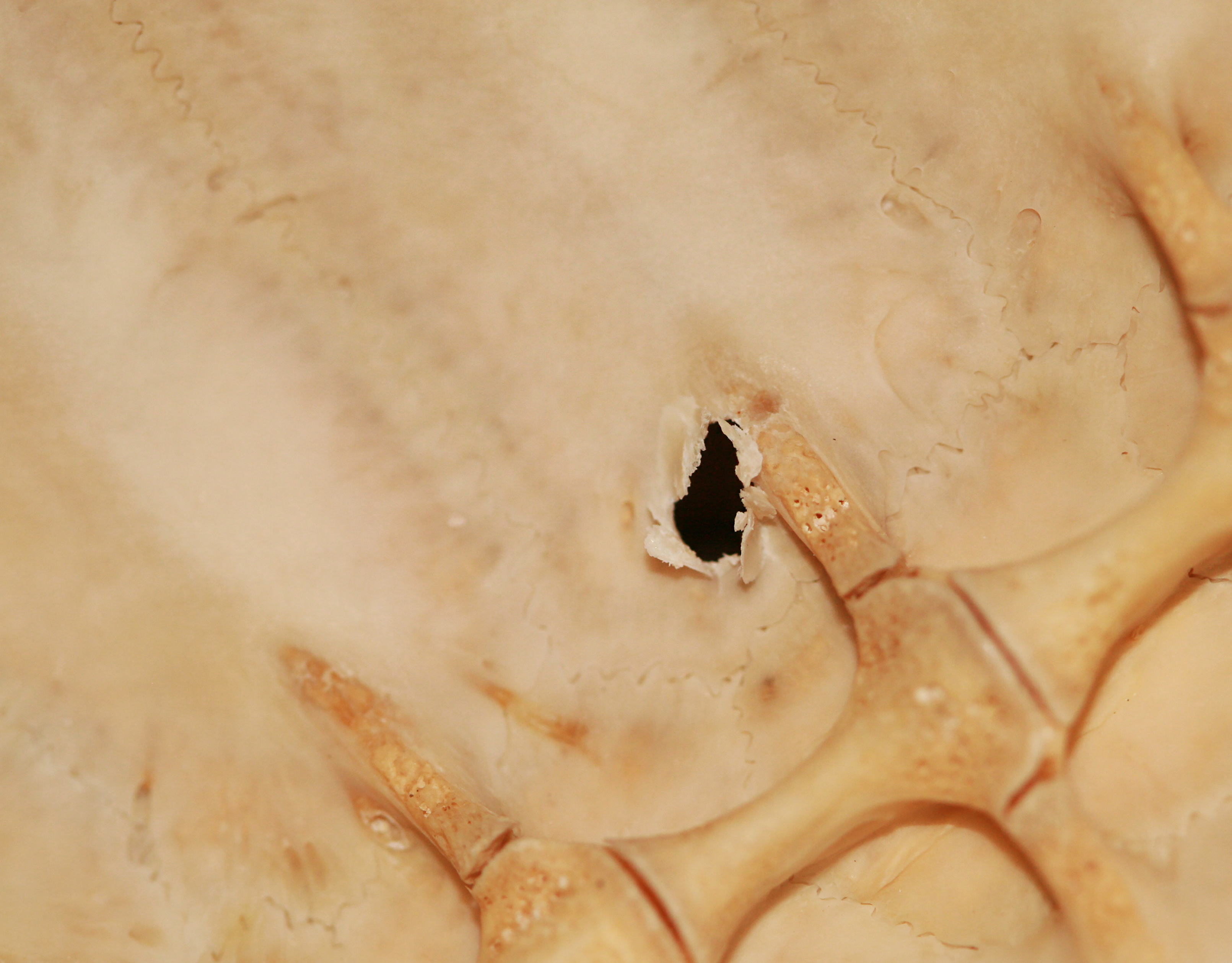

Supplement: S5 Fig — (TIF) [file pone.0201472.s007.tif]

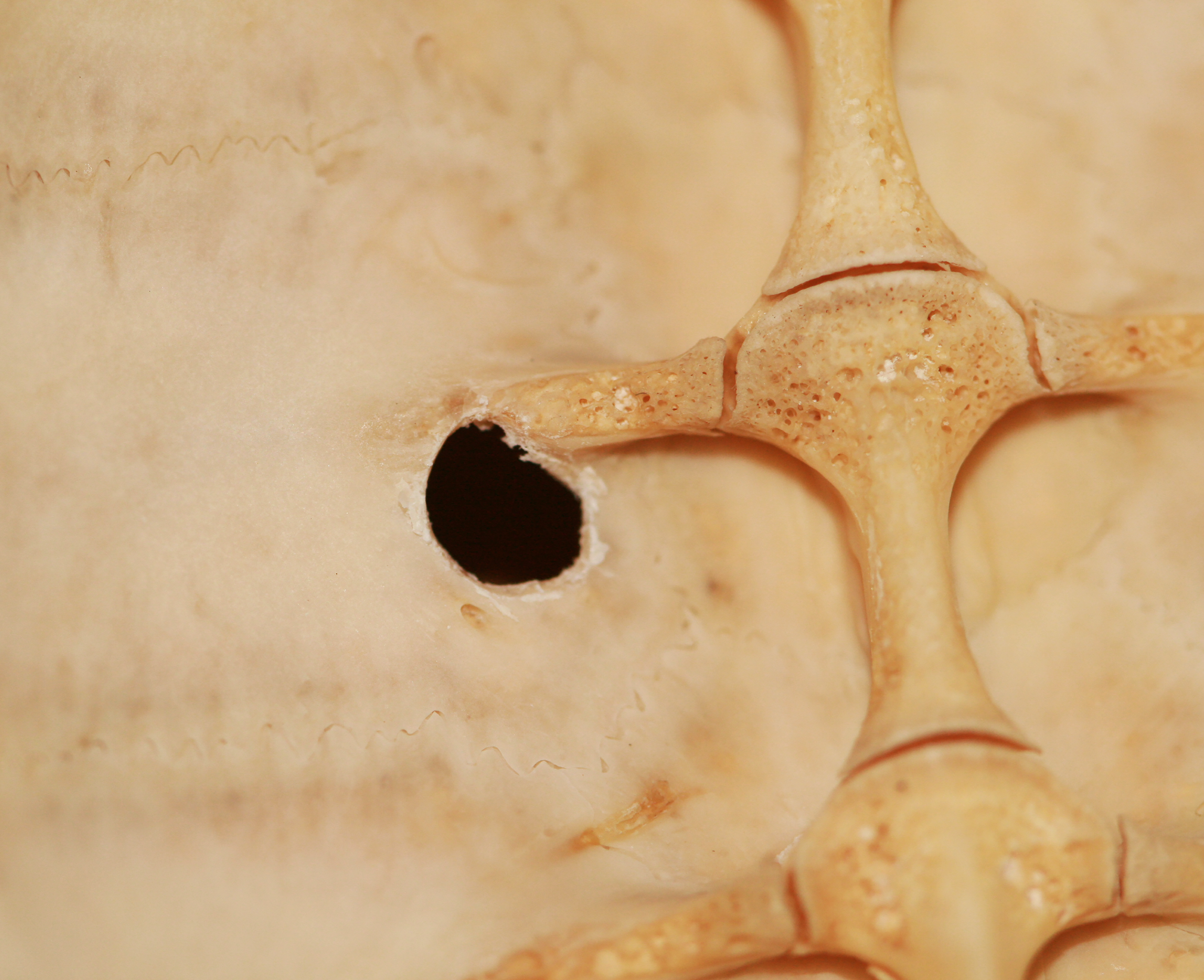

Supplement: S6 Fig — (TIF) [file pone.0201472.s008.tif]

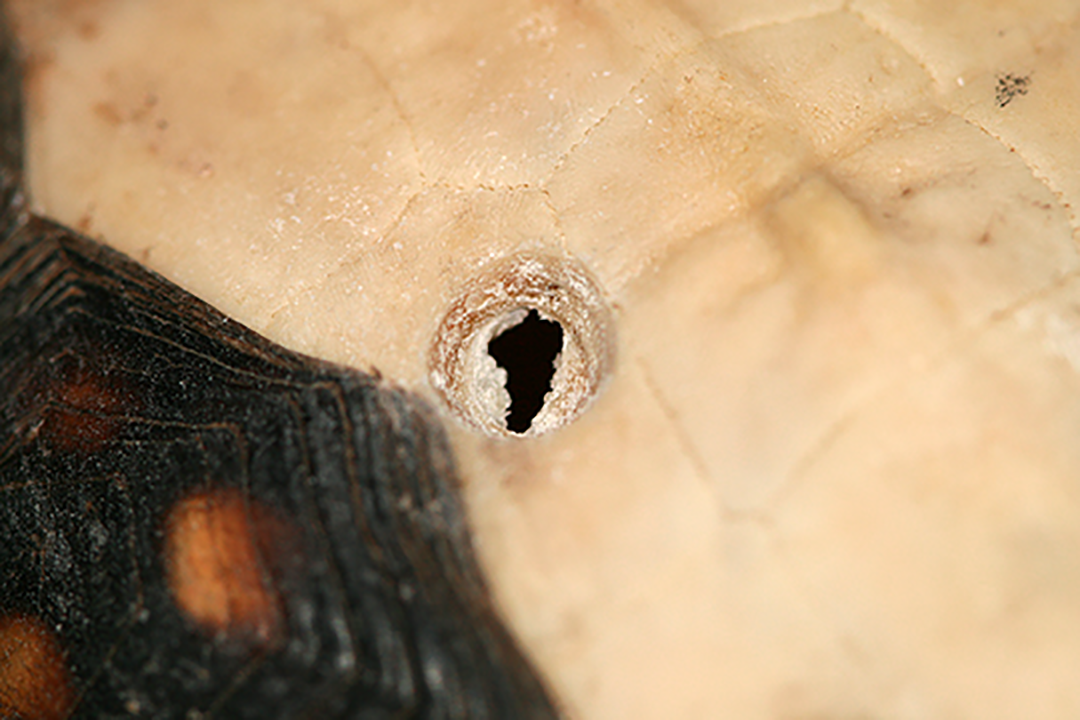

Supplement: S7 Fig — (TIF) [file pone.0201472.s009.tif]

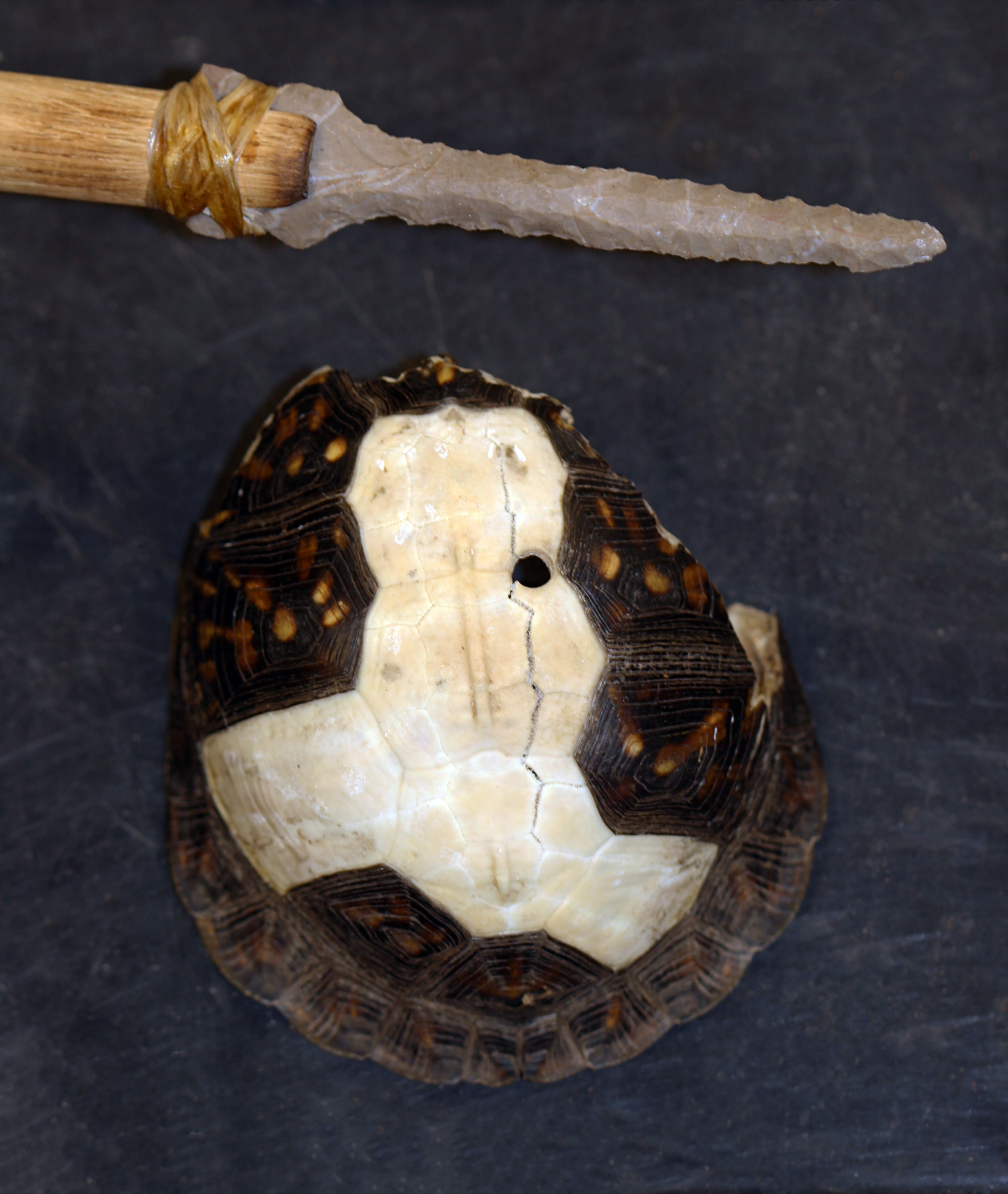

Supplement: S8 Fig — The lighter (or whiter) parts of the turtle shell are the bony plates. The darker (or browner) parts of the turtle shell are the epidermal scutes. (TIF) [file pone.0201472.s010.tif]

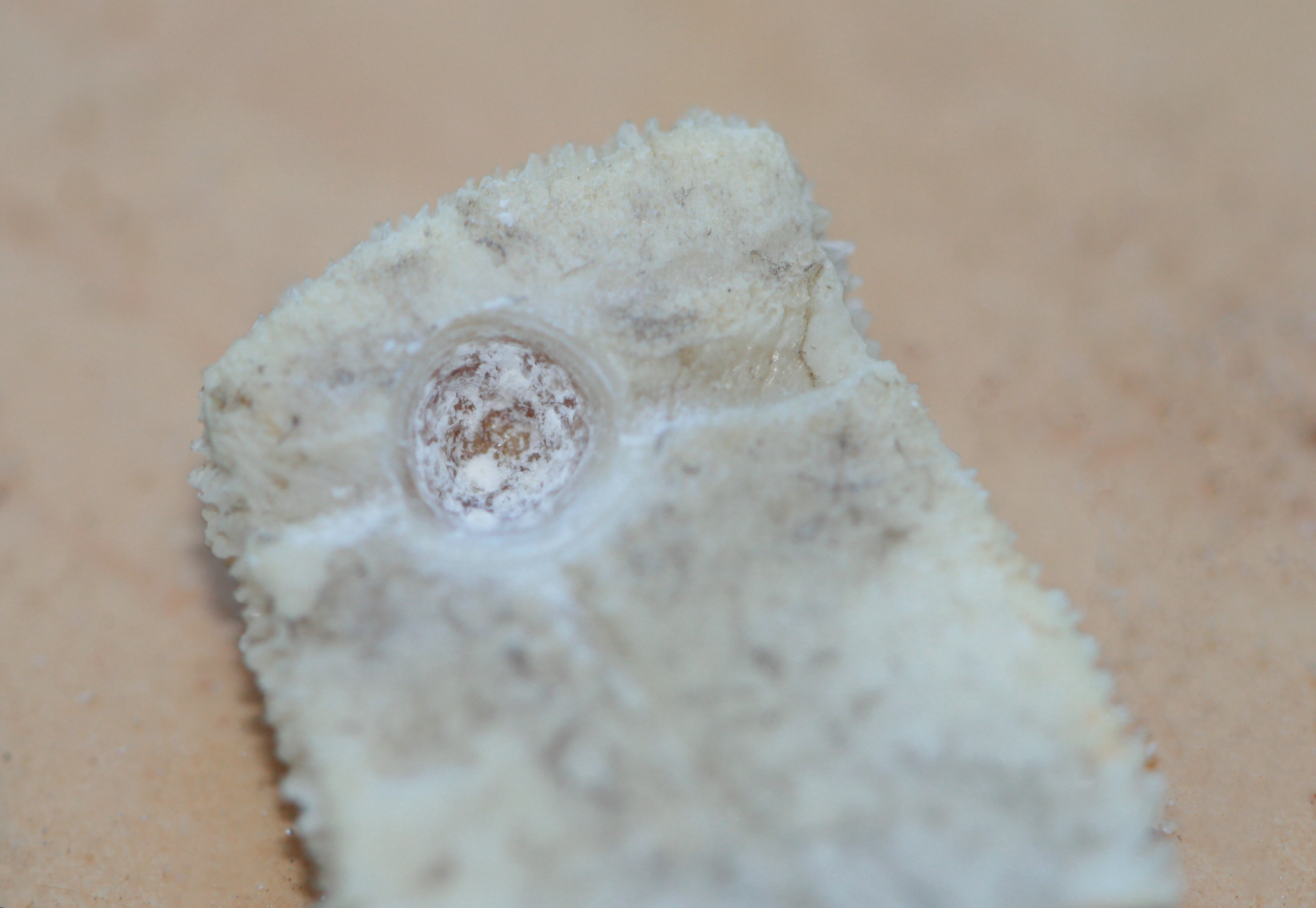

Supplement: S9 Fig — (TIF) [file pone.0201472.s011.tif]

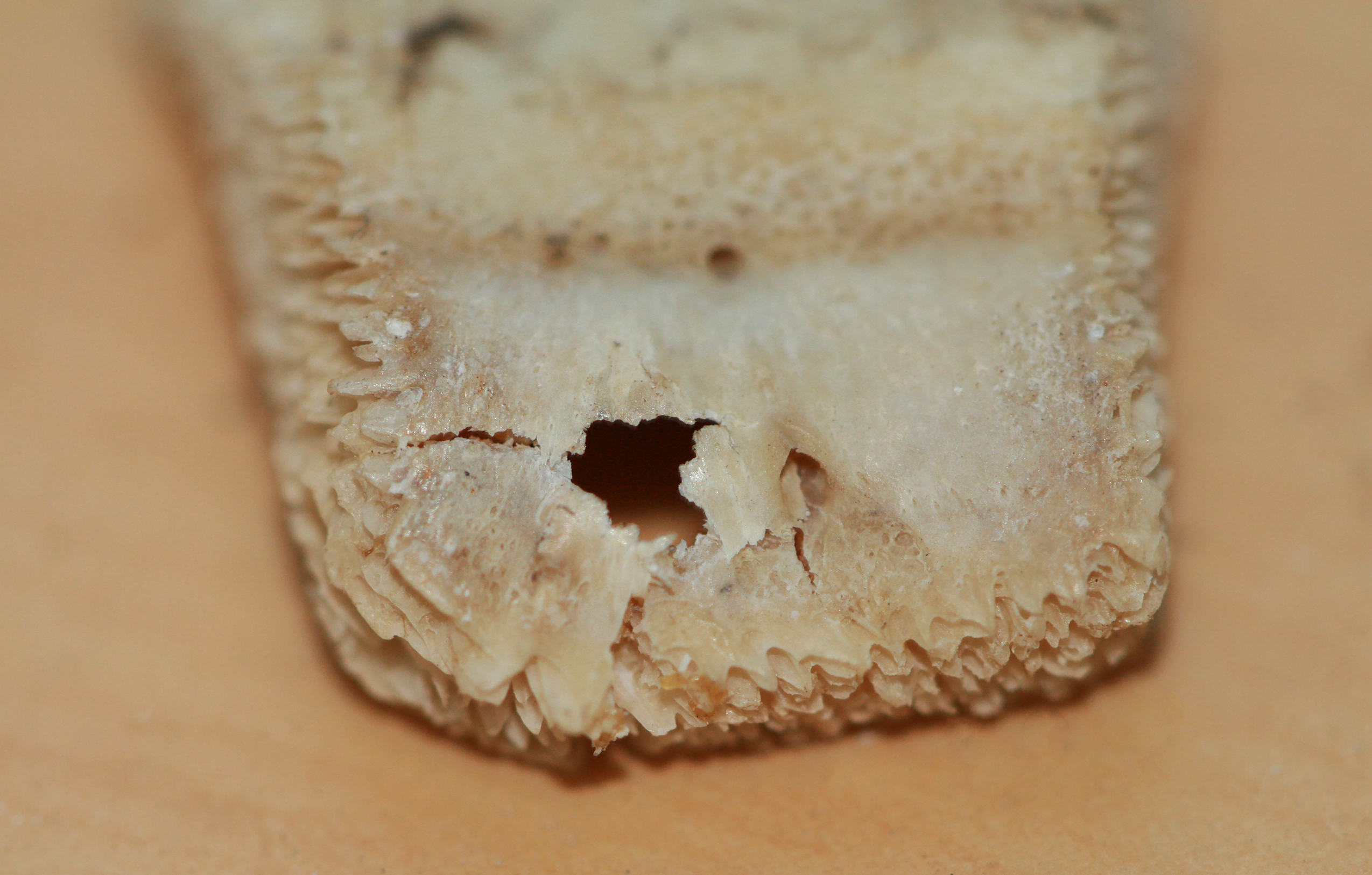

Supplement: S10 Fig — (TIF) [file pone.0201472.s012.tif]

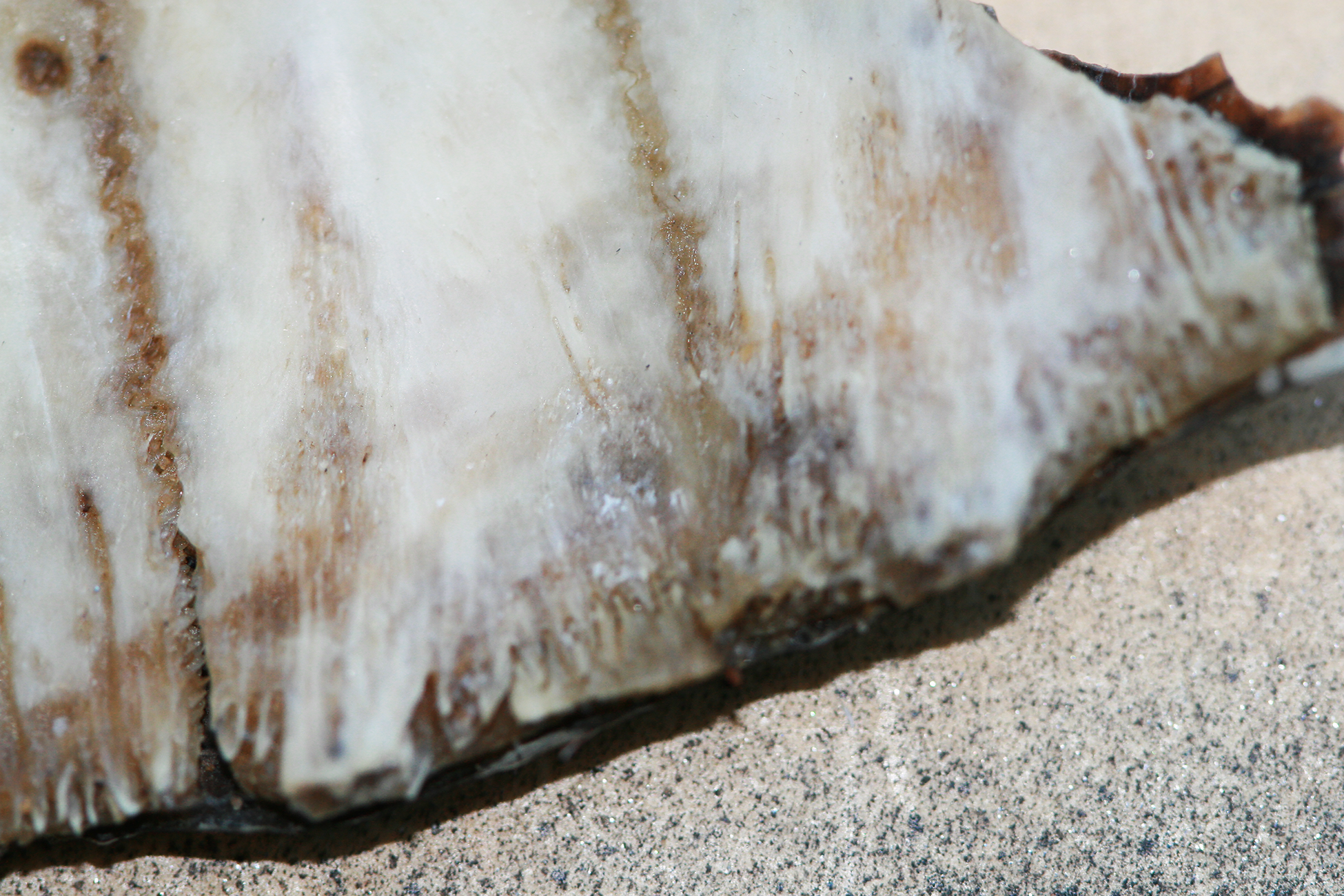

Supplement: S11 Fig — (TIF) [file pone.0201472.s013.tif]

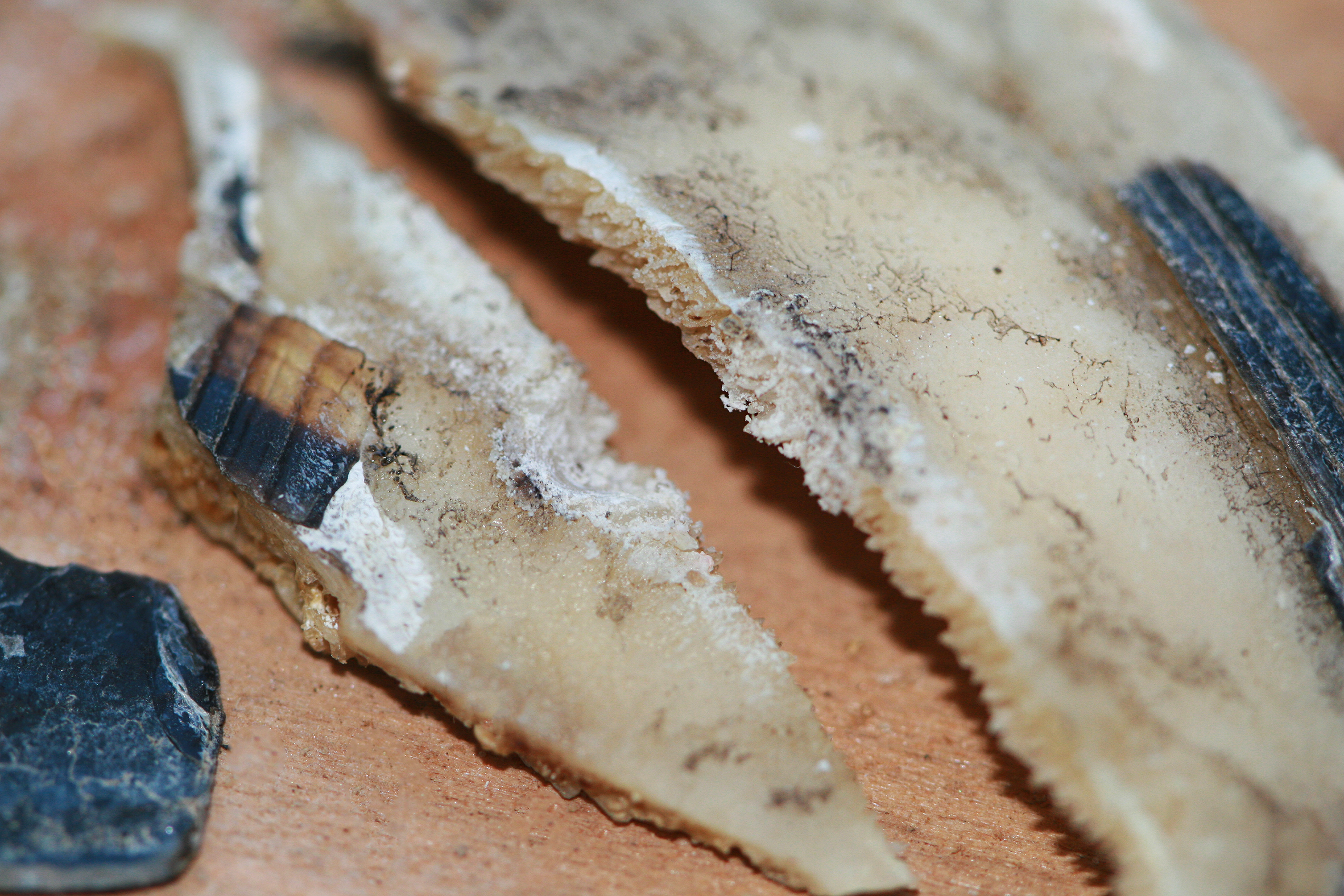

Supplement: S12 Fig — (TIF) [file pone.0201472.s014.tif]

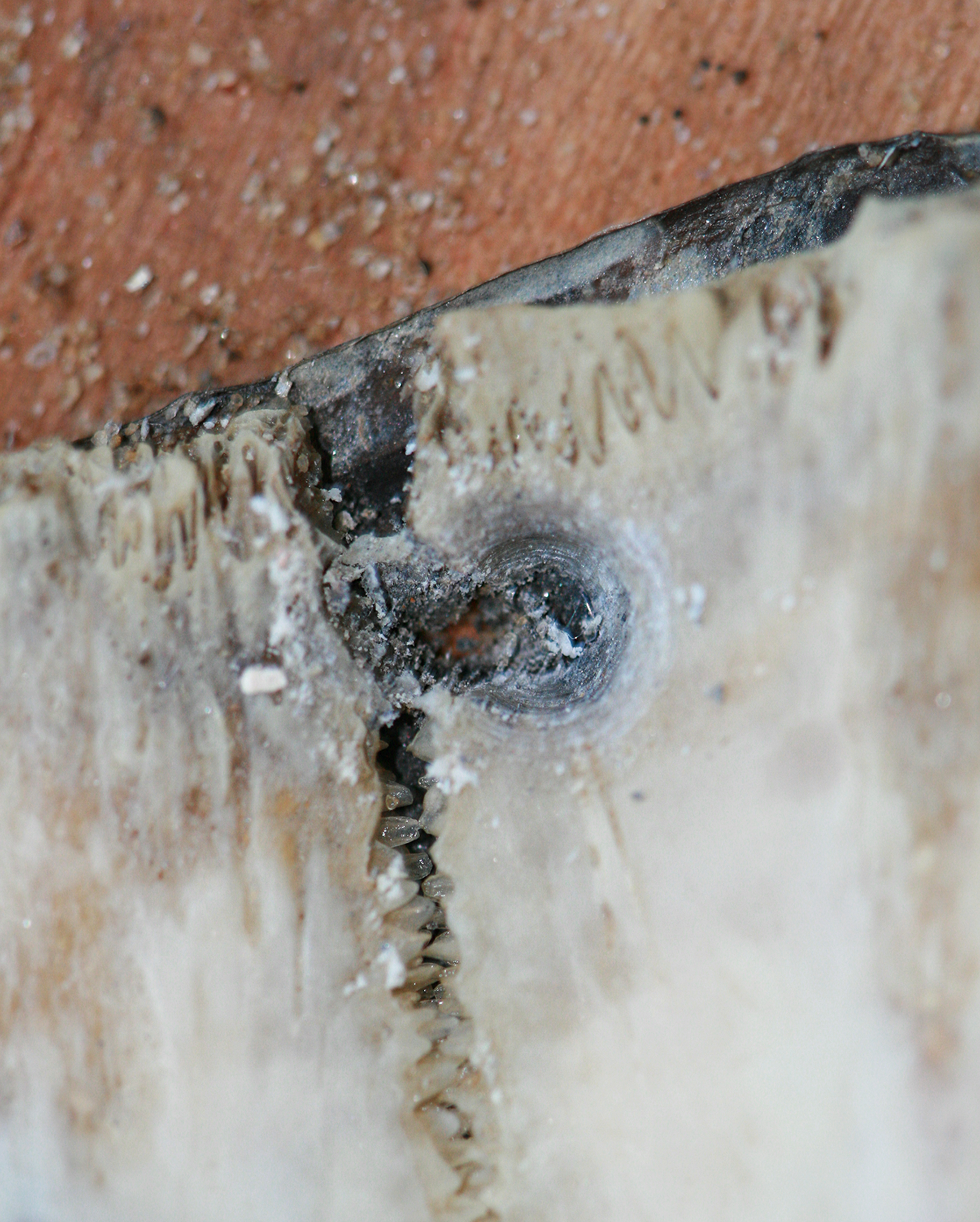

Supplement: S13 Fig — (TIF) [file pone.0201472.s015.tif]

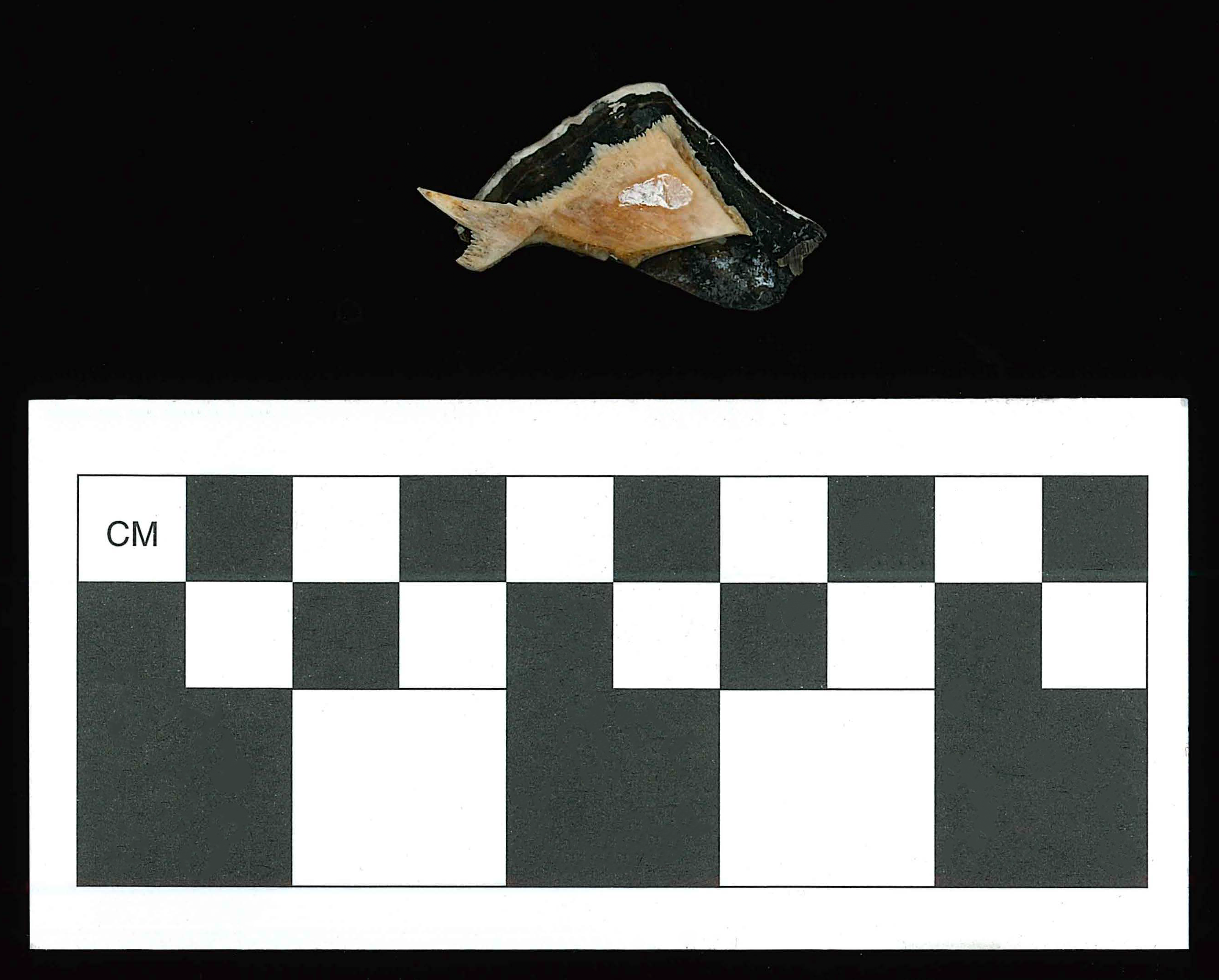

Supplement: S14 Fig — (TIF) [file pone.0201472.s016.tif]

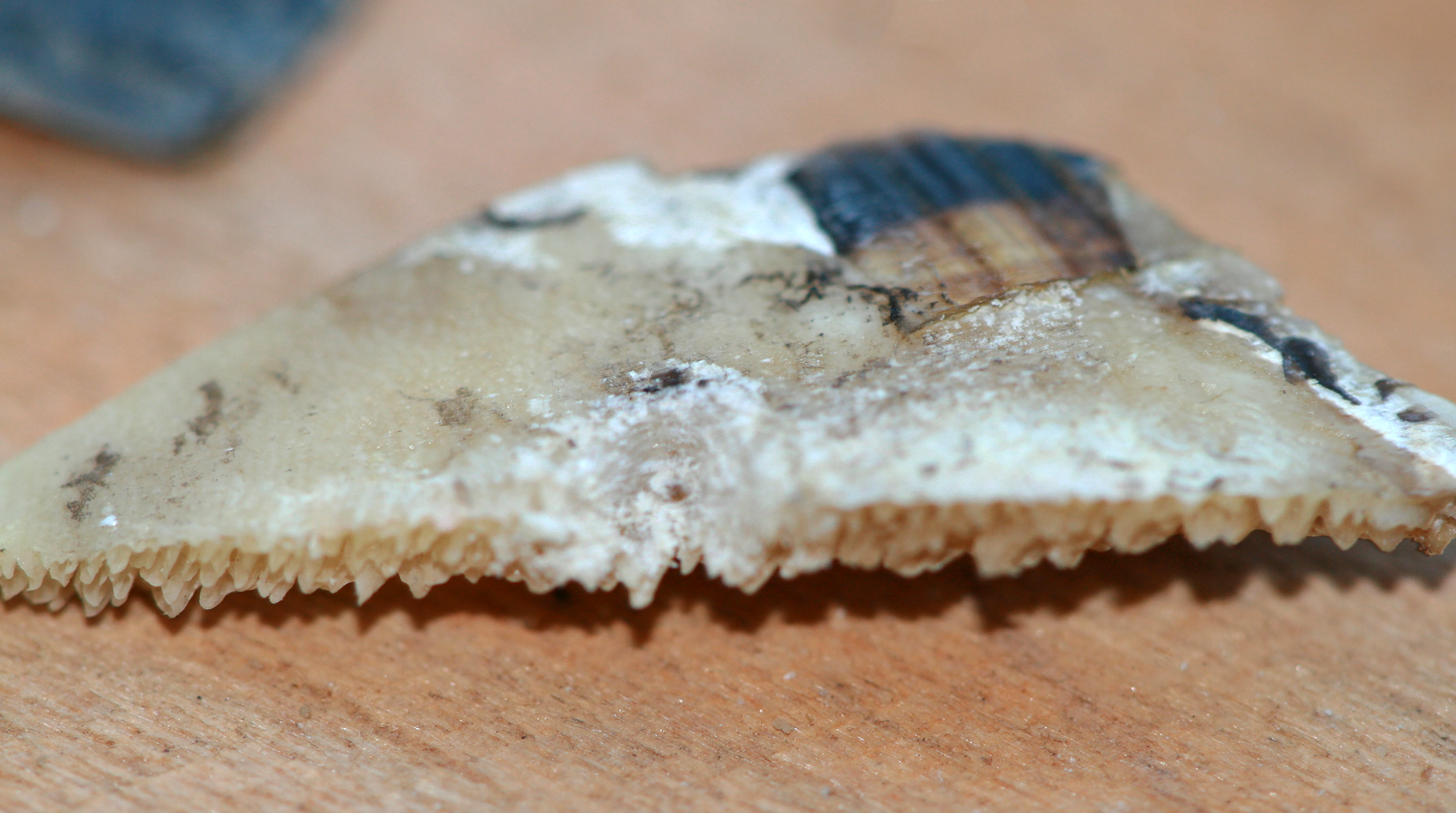

Supplement: S15 Fig — (TIF) [file pone.0201472.s017.tif]

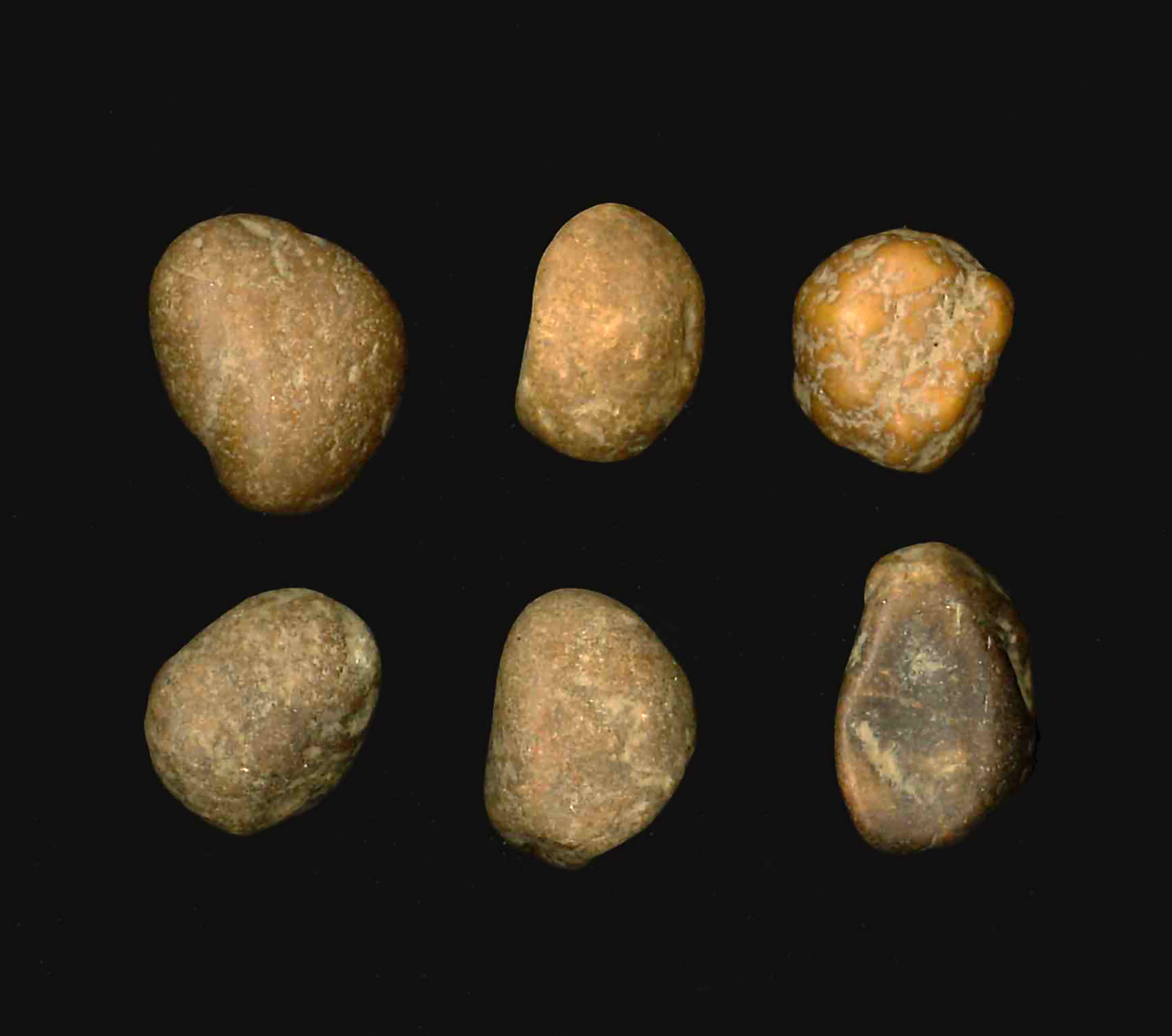

Supplement: S16 Fig — (TIF) [file pone.0201472.s018.tif]

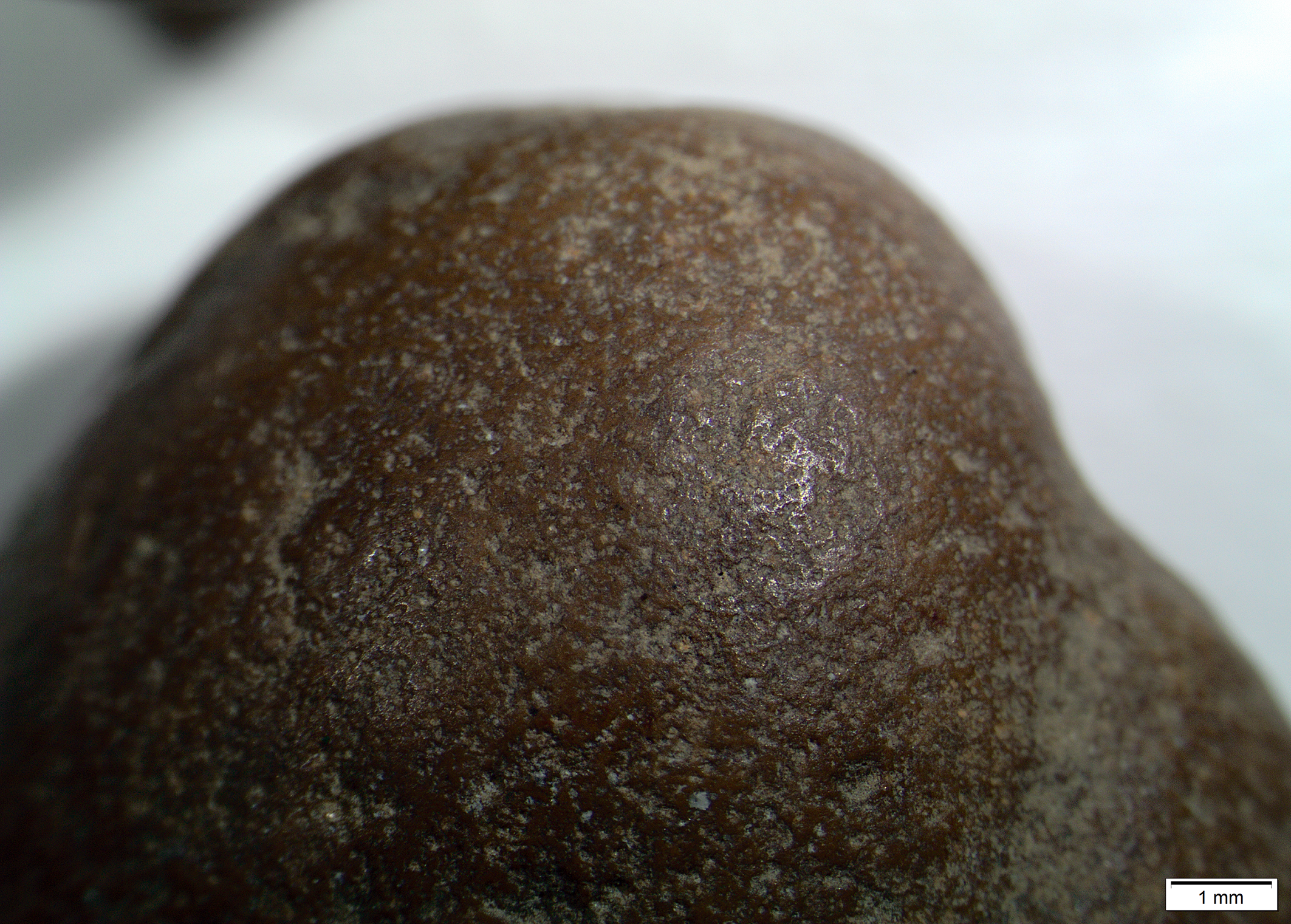

Supplement: S17 Fig — (TIF) [file pone.0201472.s019.tif]

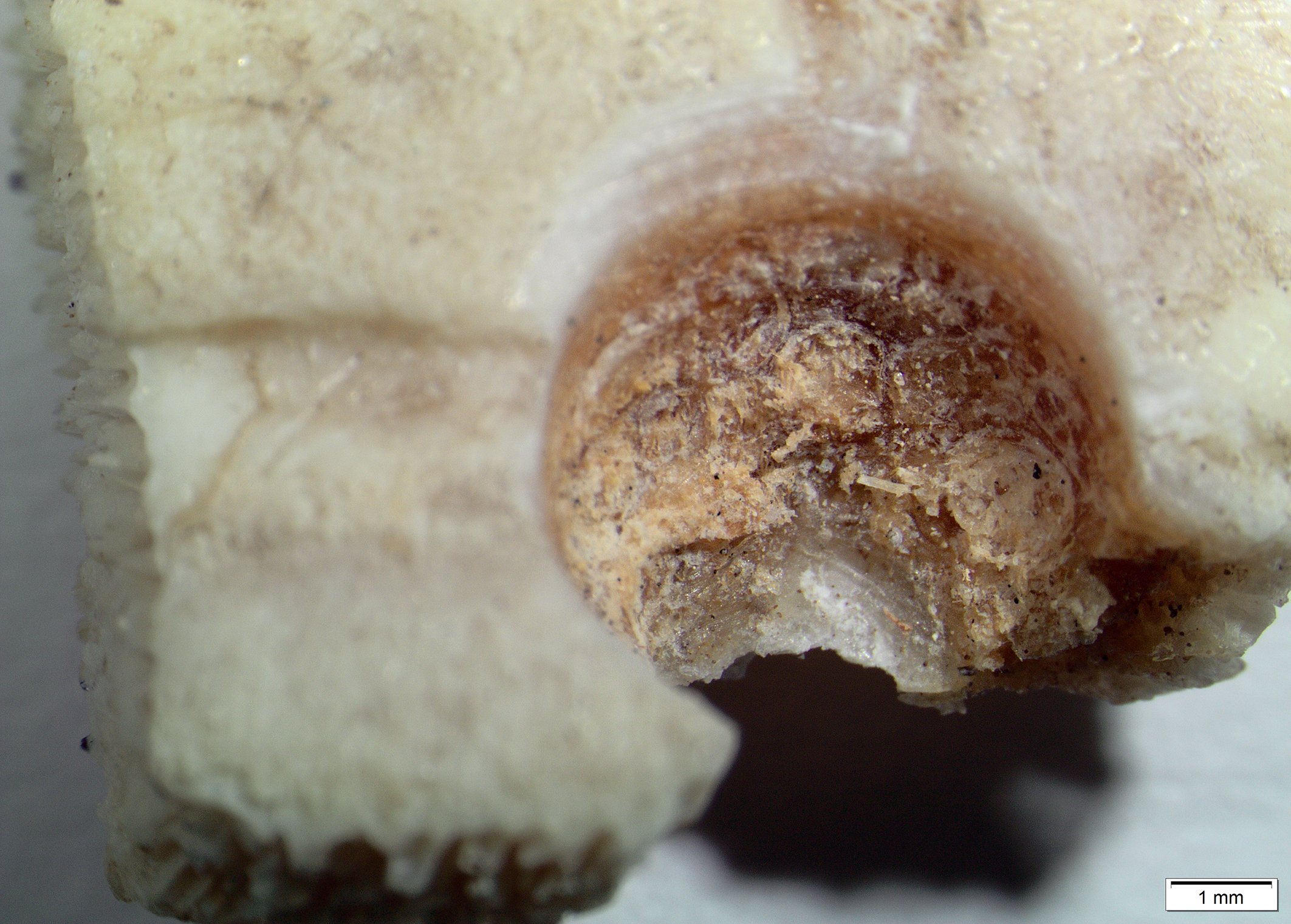

Supplement: S18 Fig — Exterior of turtle shell (from drilling side). (TIF) [file pone.0201472.s020.tif]

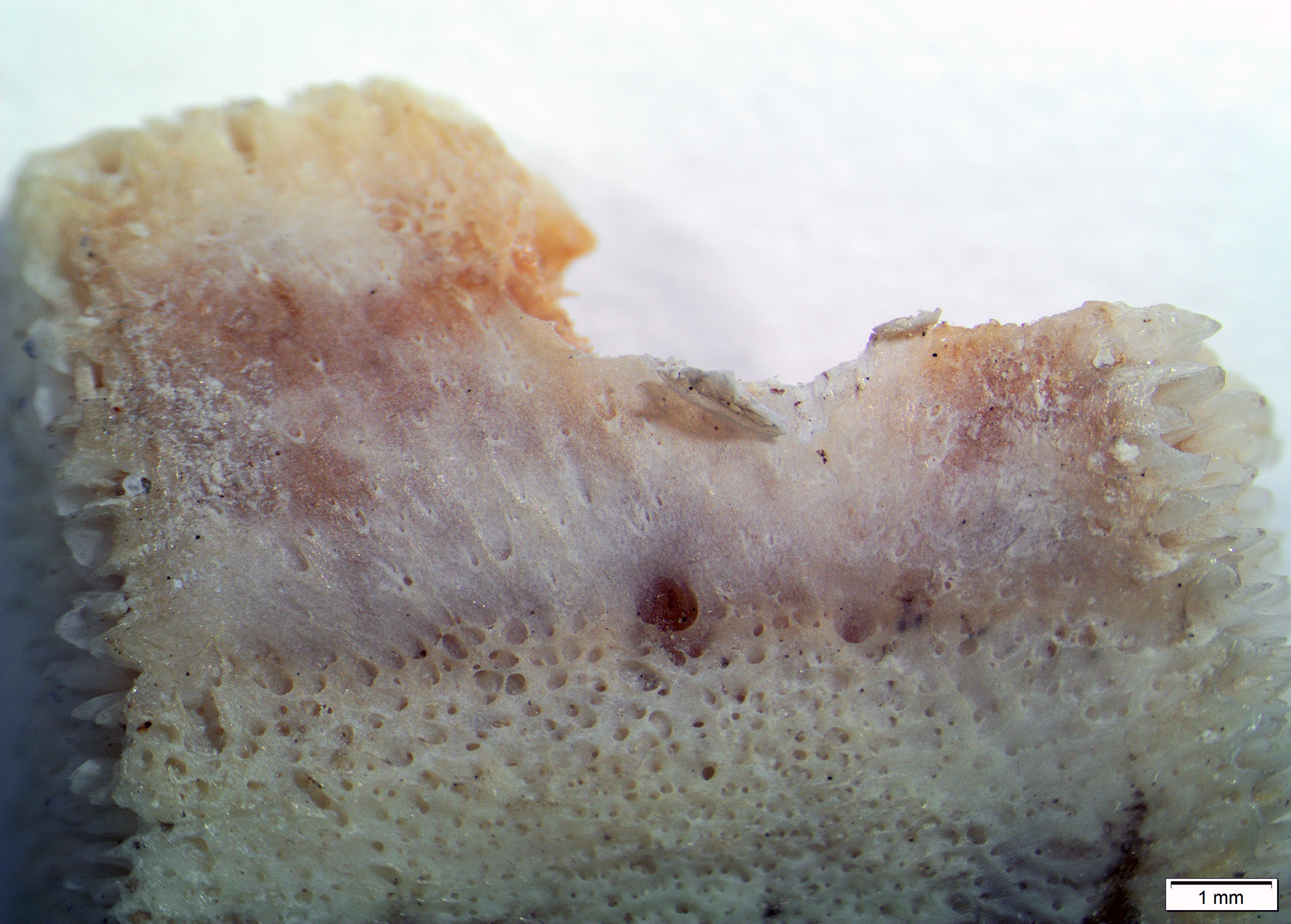

Supplement: S19 Fig — Interior of turtle shell. (TIF) [file pone.0201472.s021.tif]

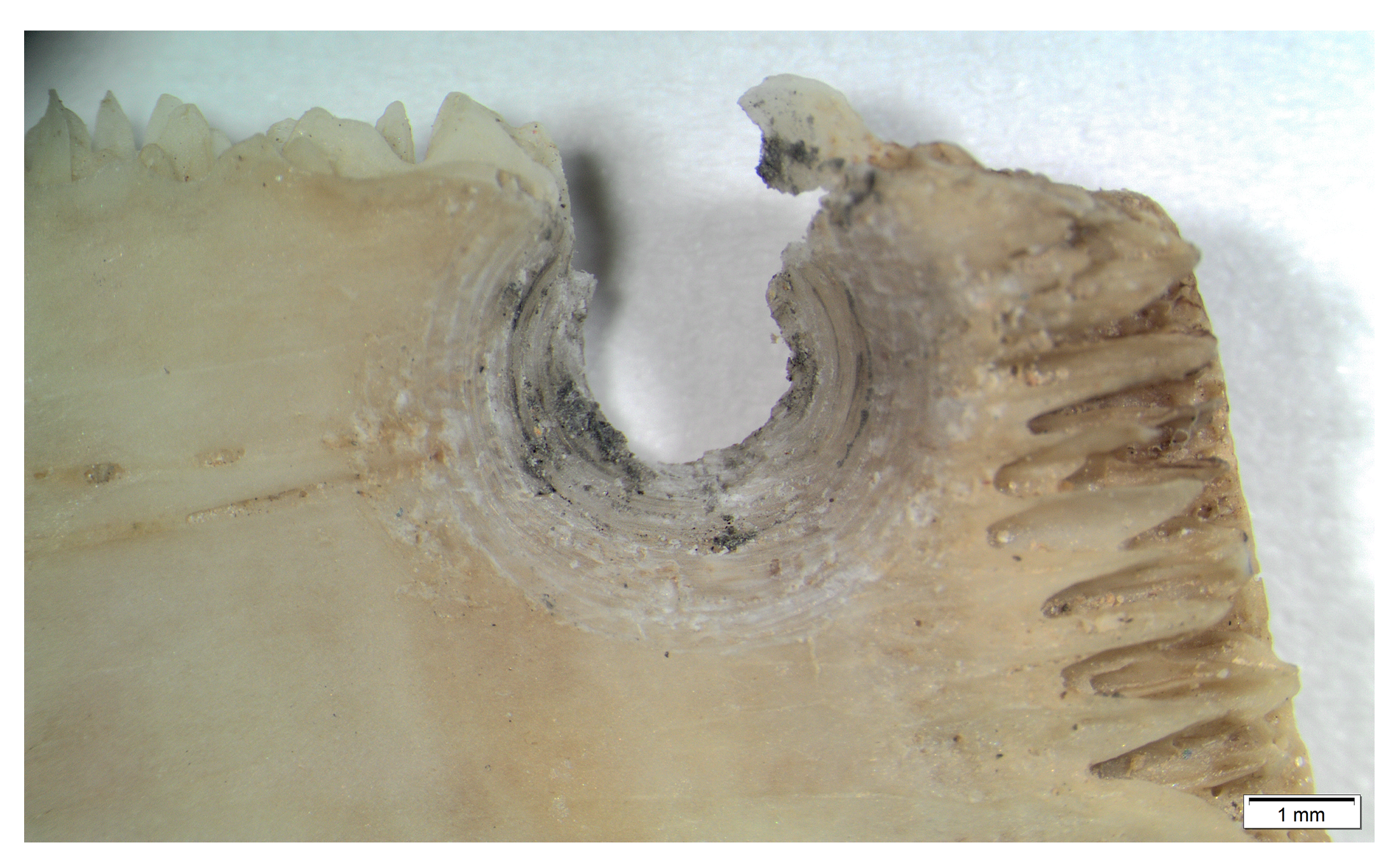

Supplement: S20 Fig — Exterior of turtle shell (drilling side). (TIF) [file pone.0201472.s022.tif]

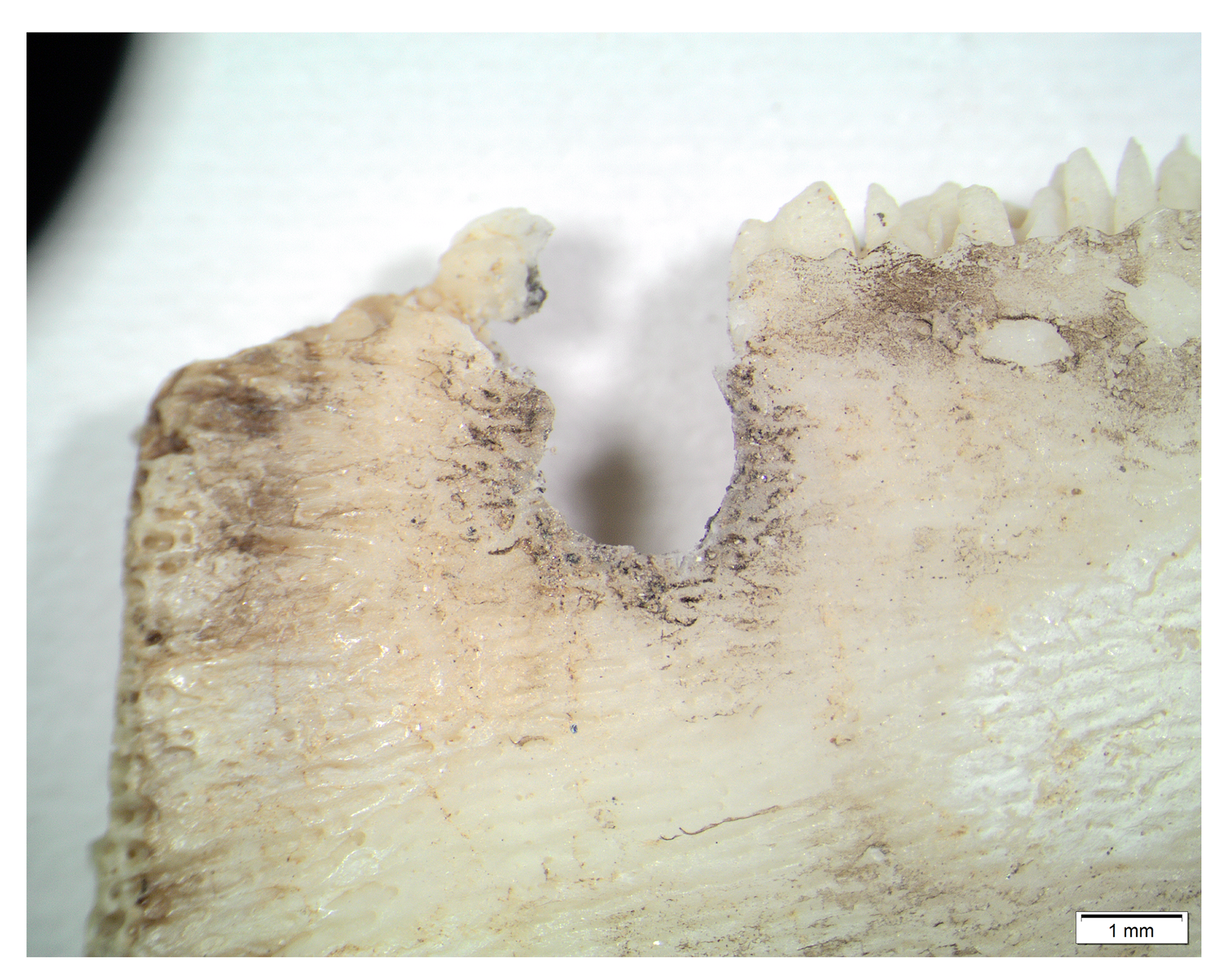

Supplement: S21 Fig — Interior of turtle shell (opposite of drilling side). (TIF) [file pone.0201472.s023.tif]

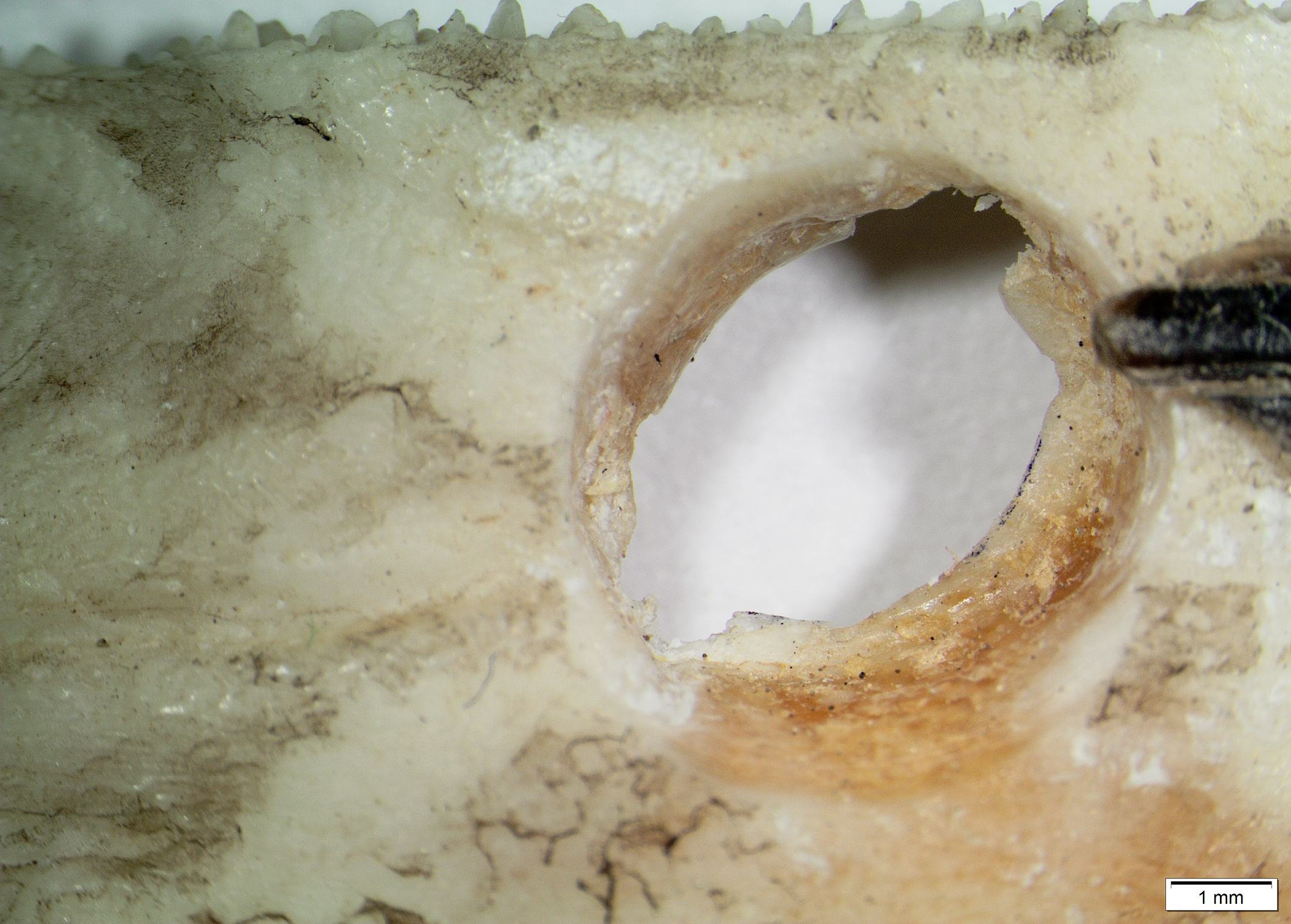

Supplement: S22 Fig — Exterior of turtle shell (drilling side). (TIF) [file pone.0201472.s024.tif]

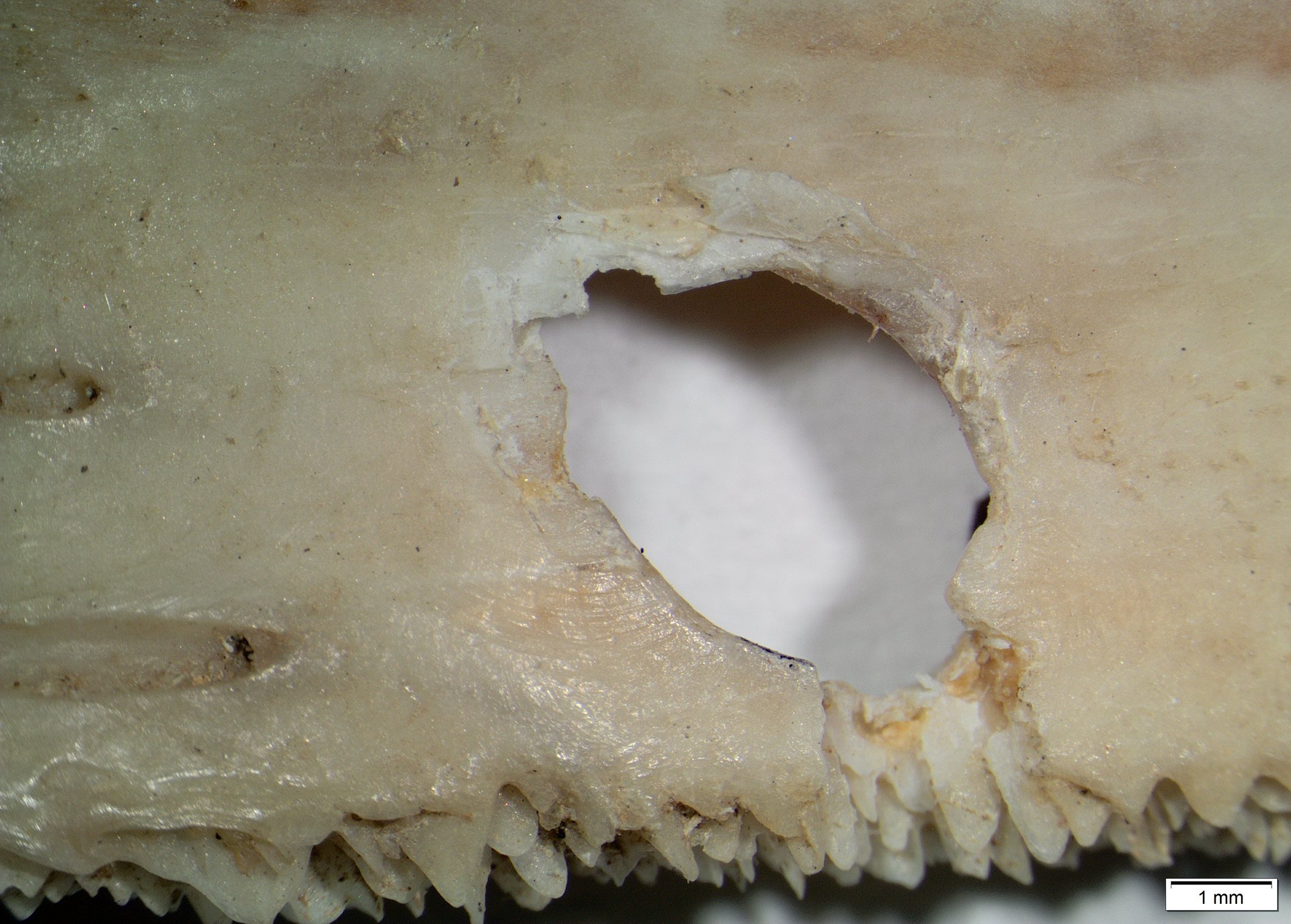

Supplement: S23 Fig — Interior of turtle shell (opposite of drilling side). (TIF) [file pone.0201472.s025.tif]

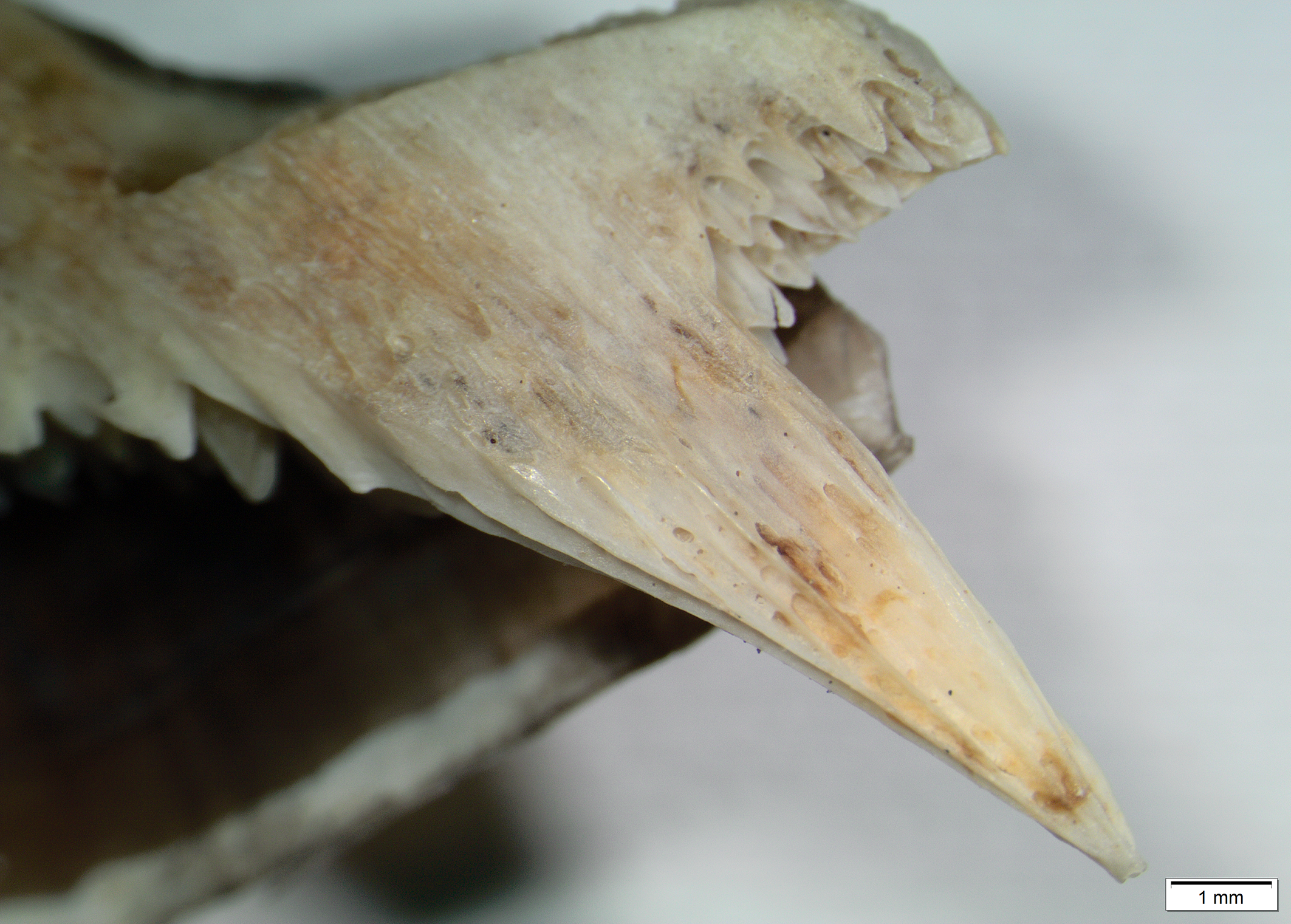

Supplement: S24 Fig — (TIF) [file pone.0201472.s026.tif]

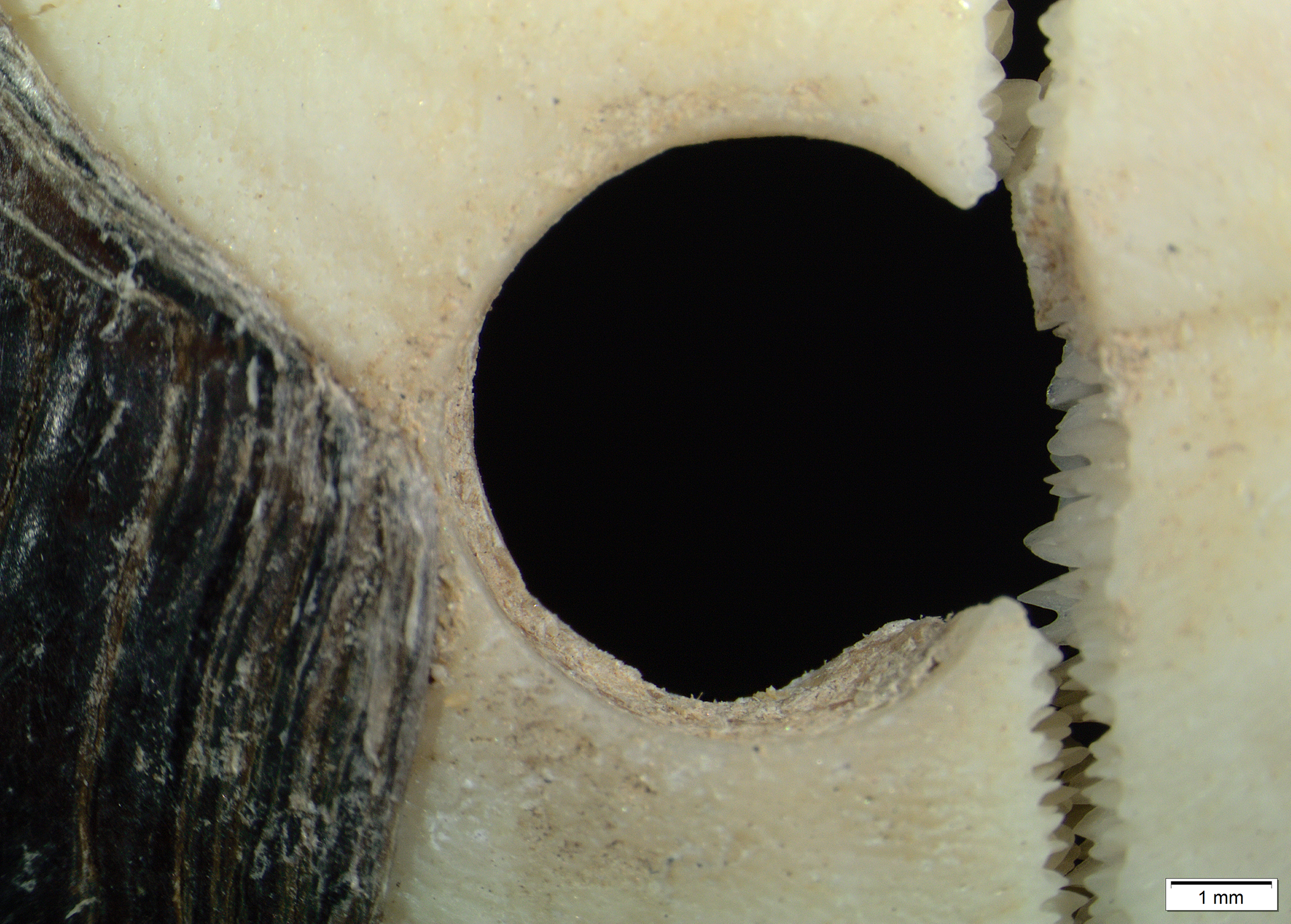

Supplement: S25 Fig — Exterior of turtle shell (drilling side). (TIF) [file pone.0201472.s027.tif]

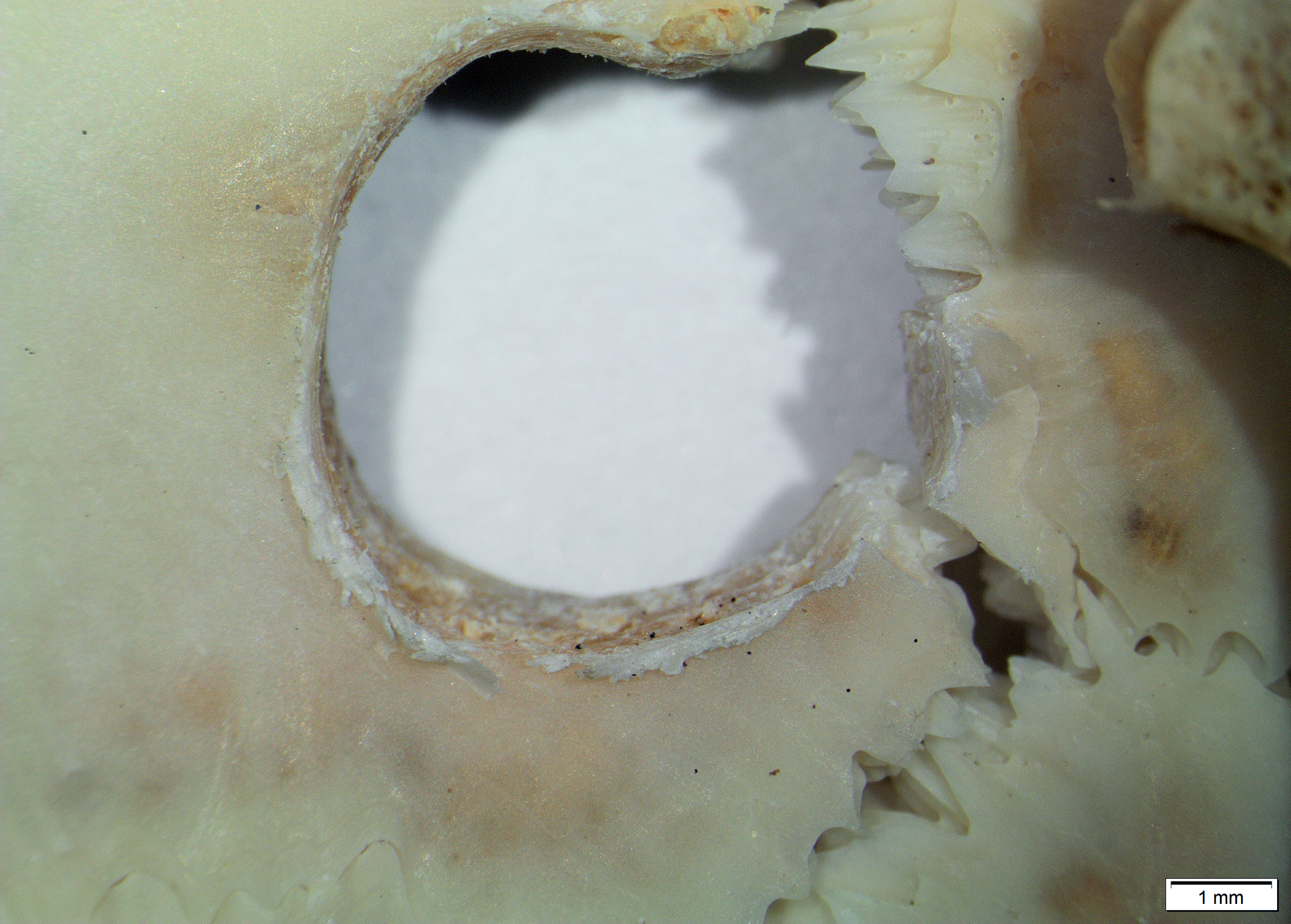

Supplement: S26 Fig — Interior of turtle shell (opposite of drilling side). (TIF) [file pone.0201472.s028.tif]

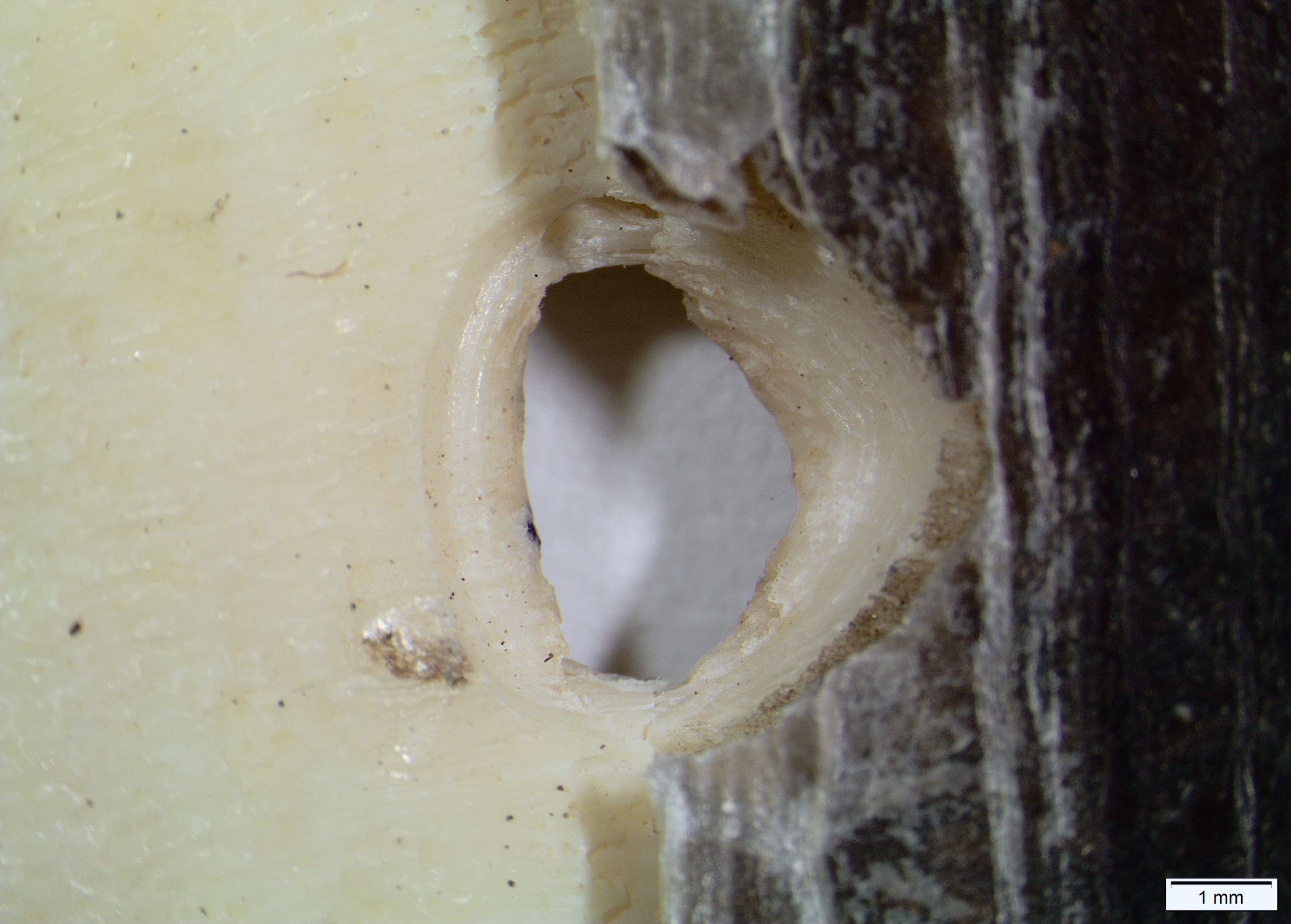

Supplement: S27 Fig — Exterior of turtle shell (drilling side). (TIF) [file pone.0201472.s029.tif]

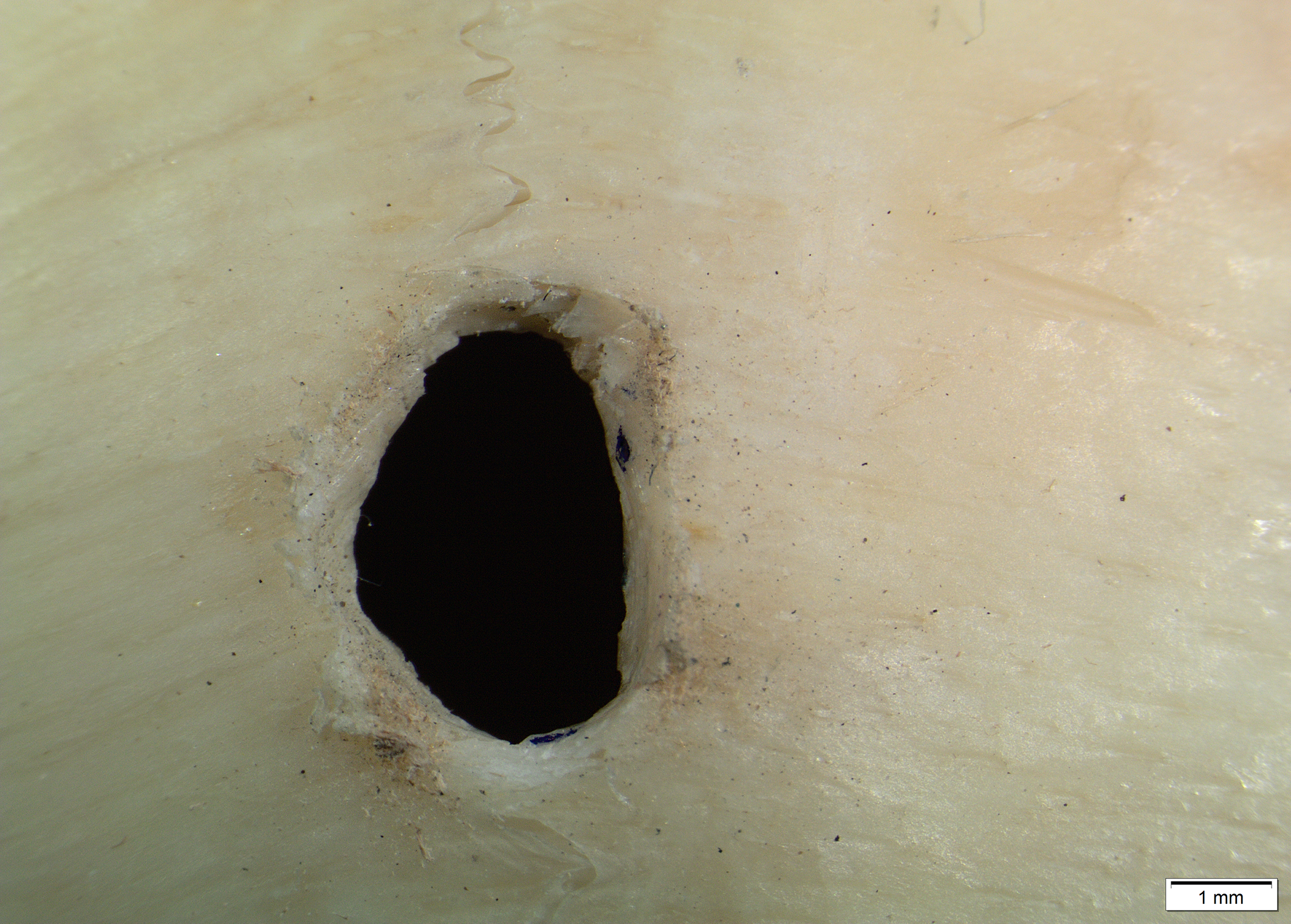

Supplement: S28 Fig — Interior of turtle shell (opposite of drilling side). (TIF) [file pone.0201472.s030.tif]

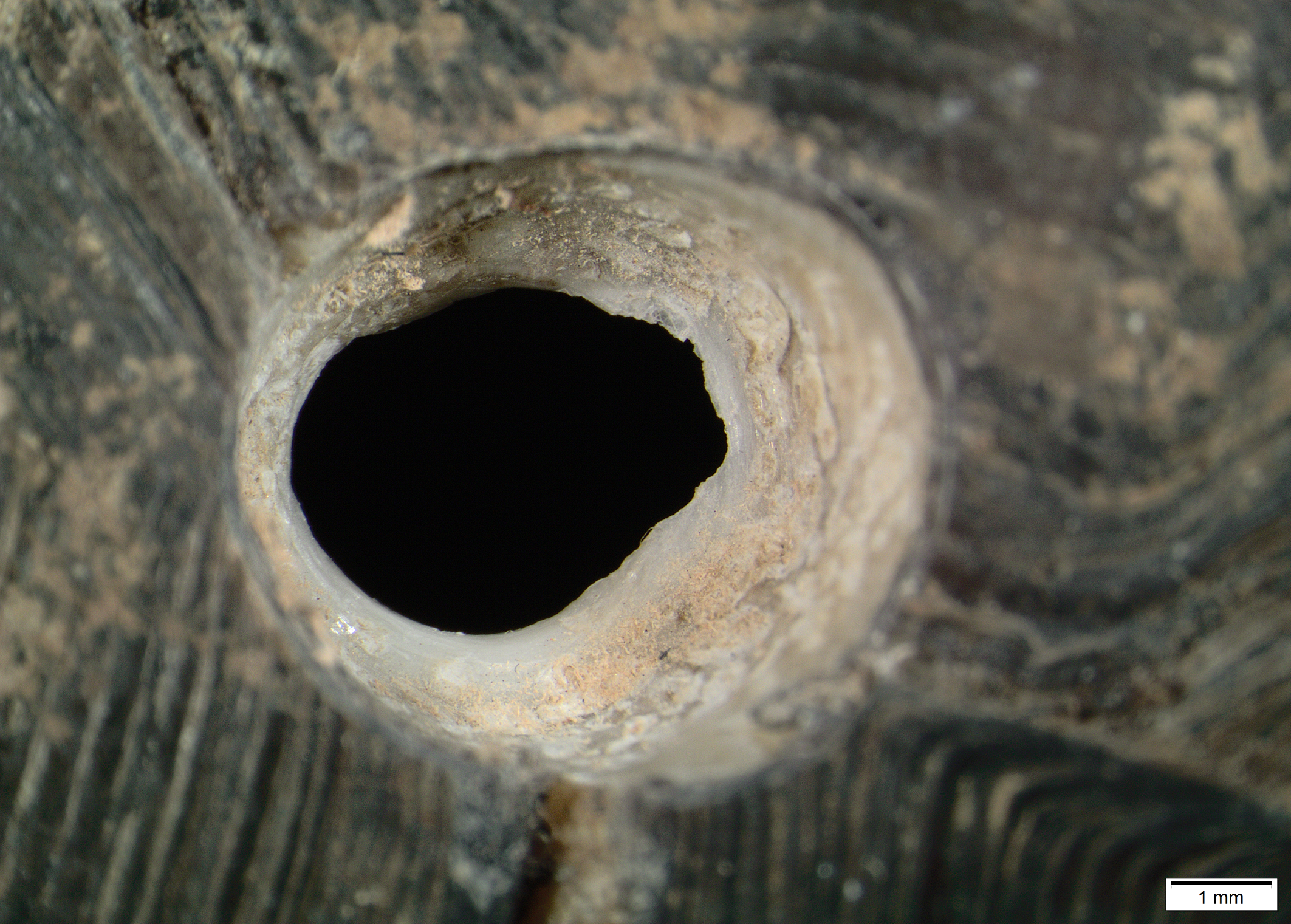

Supplement: S29 Fig — Exterior of turtle shell (drilling side). (TIF) [file pone.0201472.s031.tif]

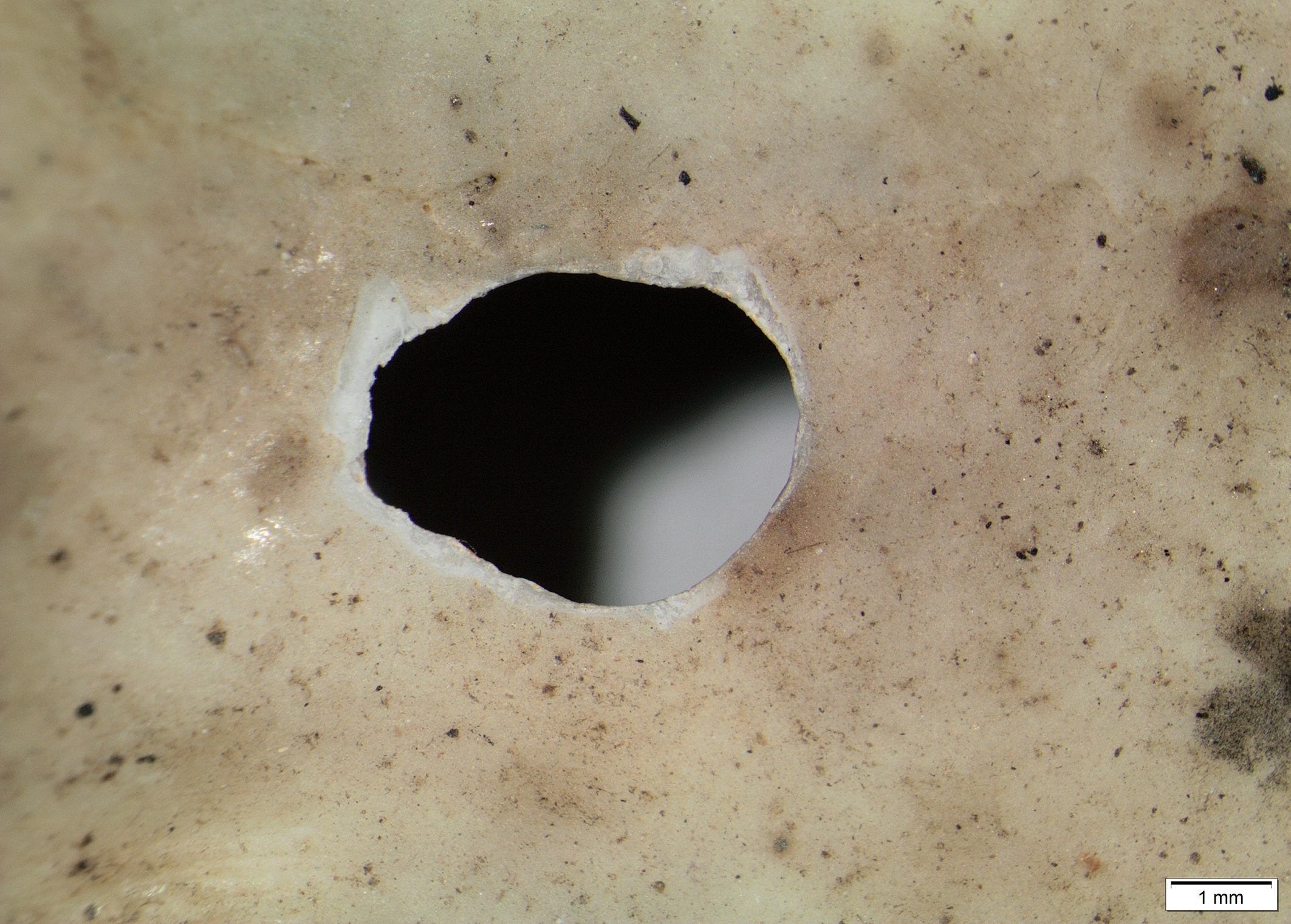

Supplement: S30 Fig — Interior of turtle shell (opposite of drilling side). (TIF) [file pone.0201472.s032.tif]

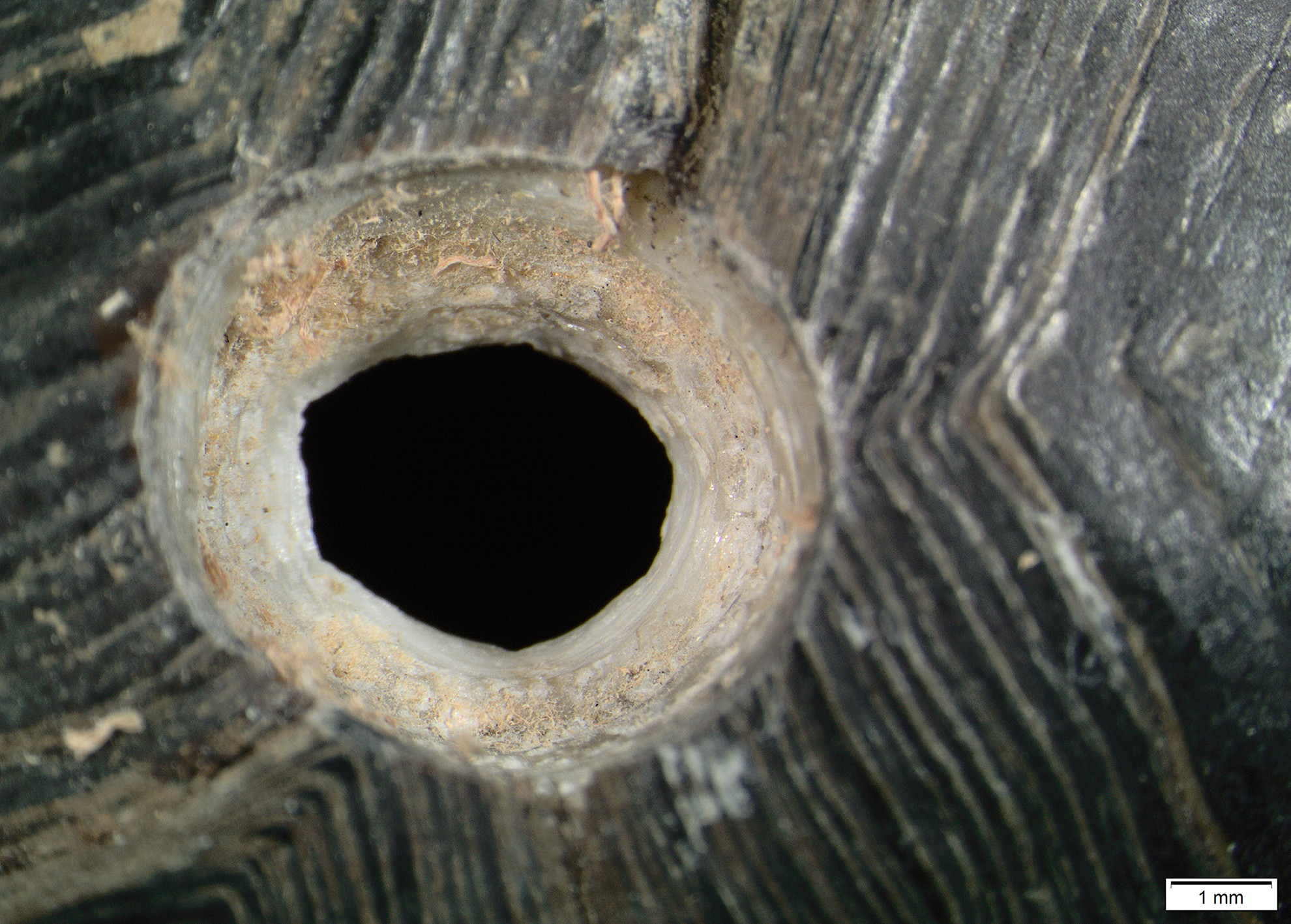

Supplement: S31 Fig — Exterior of turtle shell (drilling side). (TIF) [file pone.0201472.s033.tif]

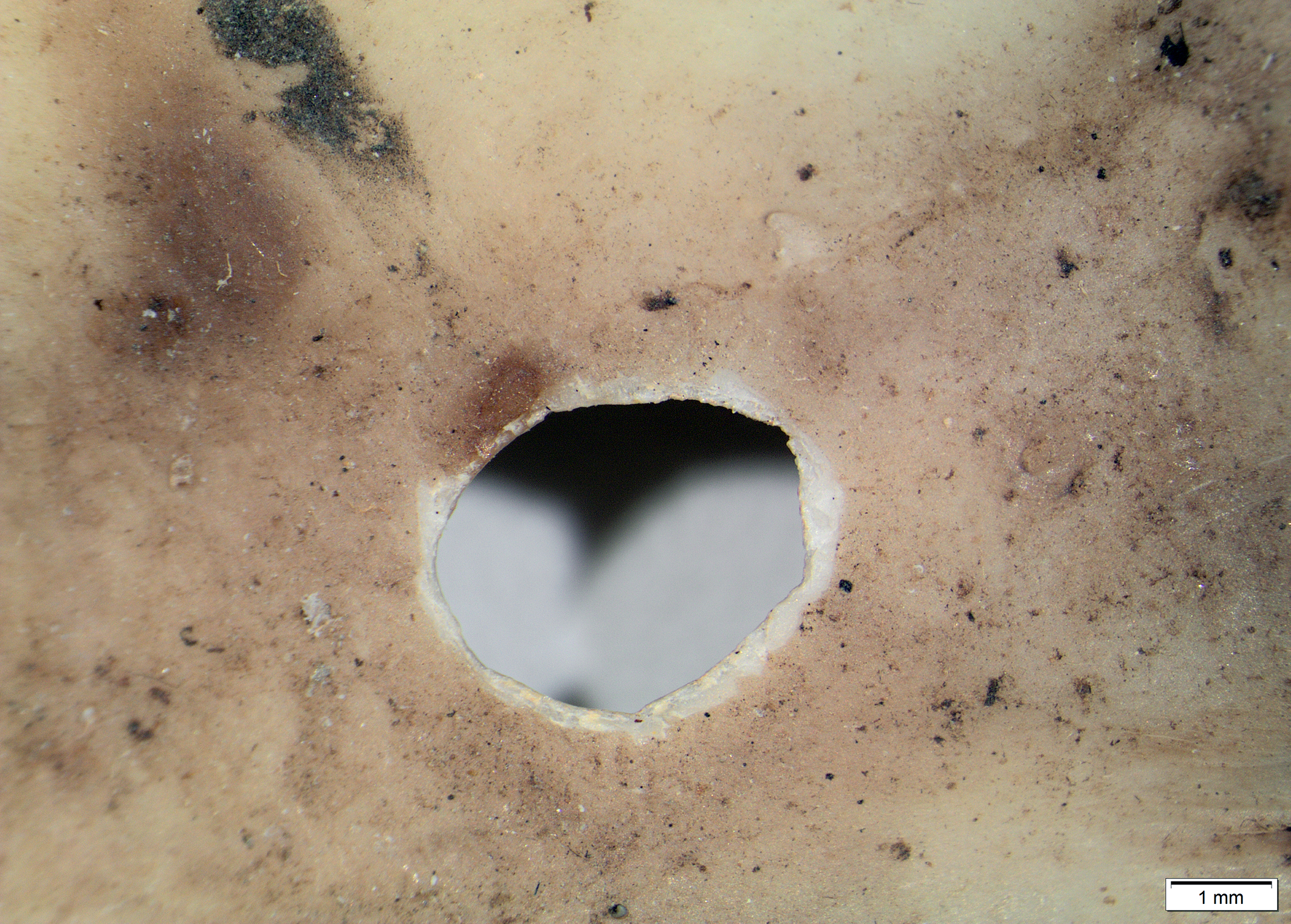

Supplement: S32 Fig — Interior of turtle shell (opposite of drilling side). (TIF) [file pone.0201472.s034.tif]

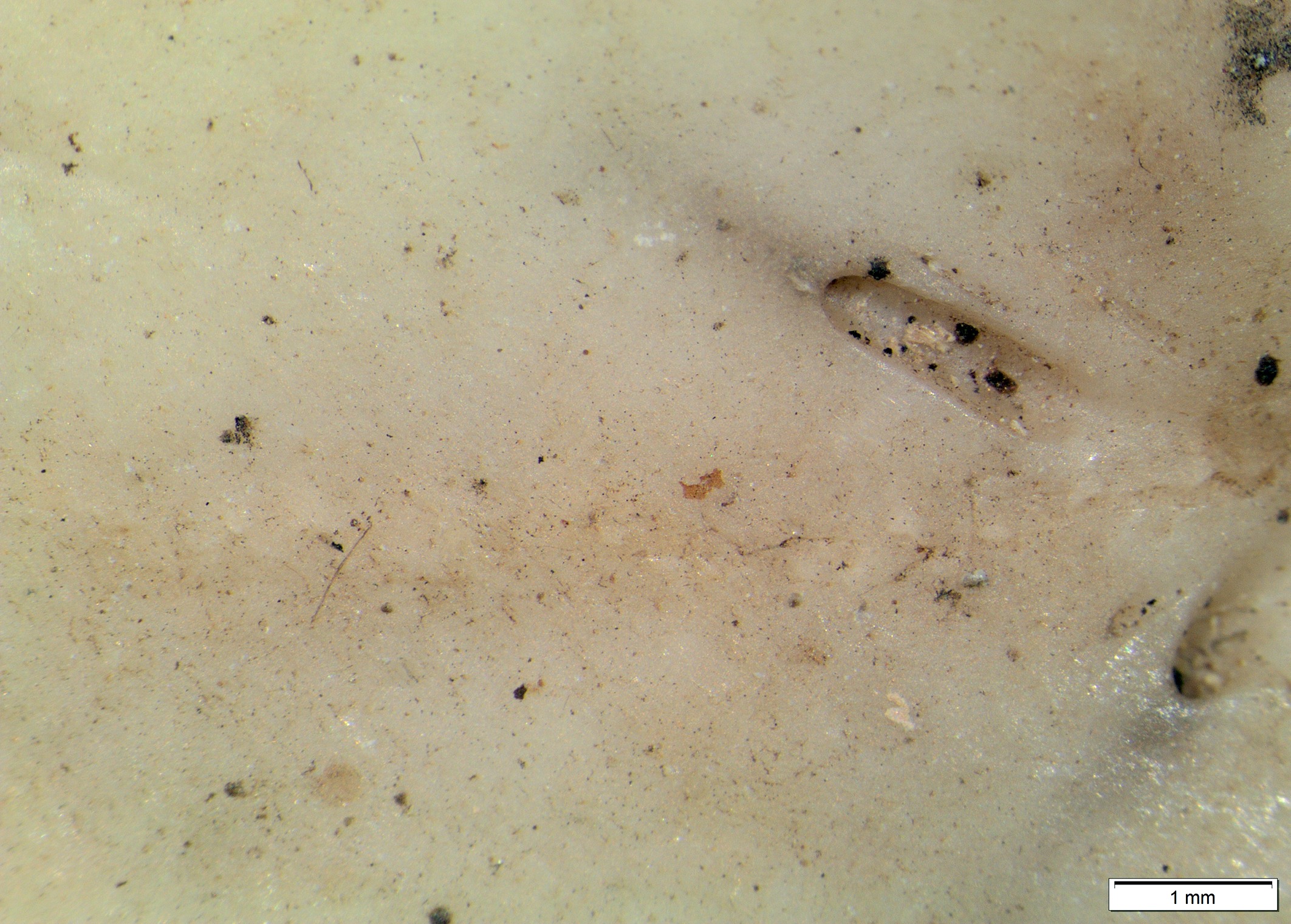

Supplement: S33 Fig — (TIF) [file pone.0201472.s035.tif]

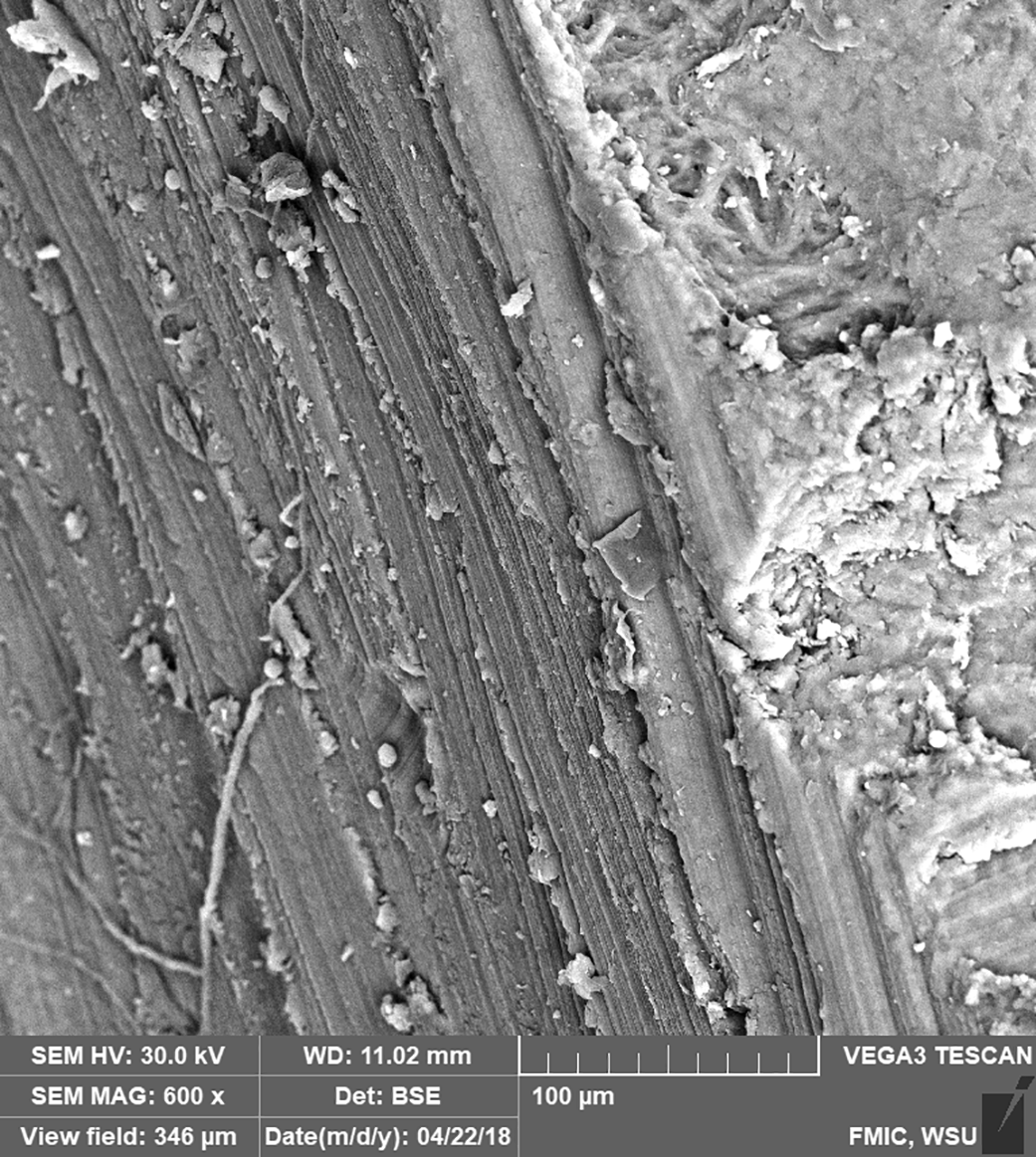

Supplement: S34 Fig — The exterior of the turtle shell can be seen on the very right side of the image. Image was captured on a Tescan Vega 3 SEM at 600x magnification. Bar = 100 μm. (TIF) [file pone.0201472.s036.tif]

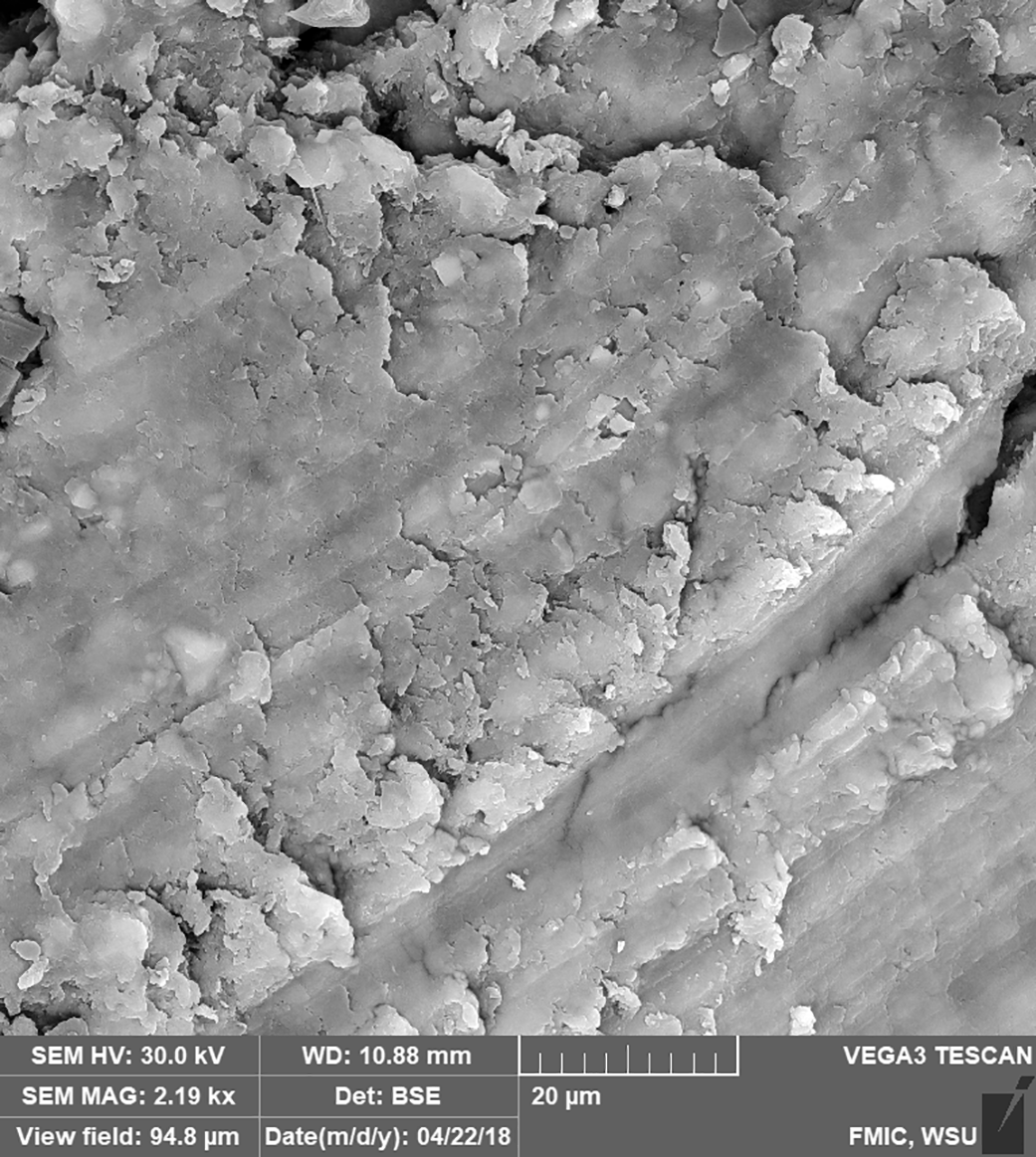

Supplement: S35 Fig — Image was taken close to the upper edge of the drill hole. Image was captured on a Tescan Vega 3 SEM at 2,190x magnification. Bar = 20 μm. (TIF) [file pone.0201472.s037.tif]

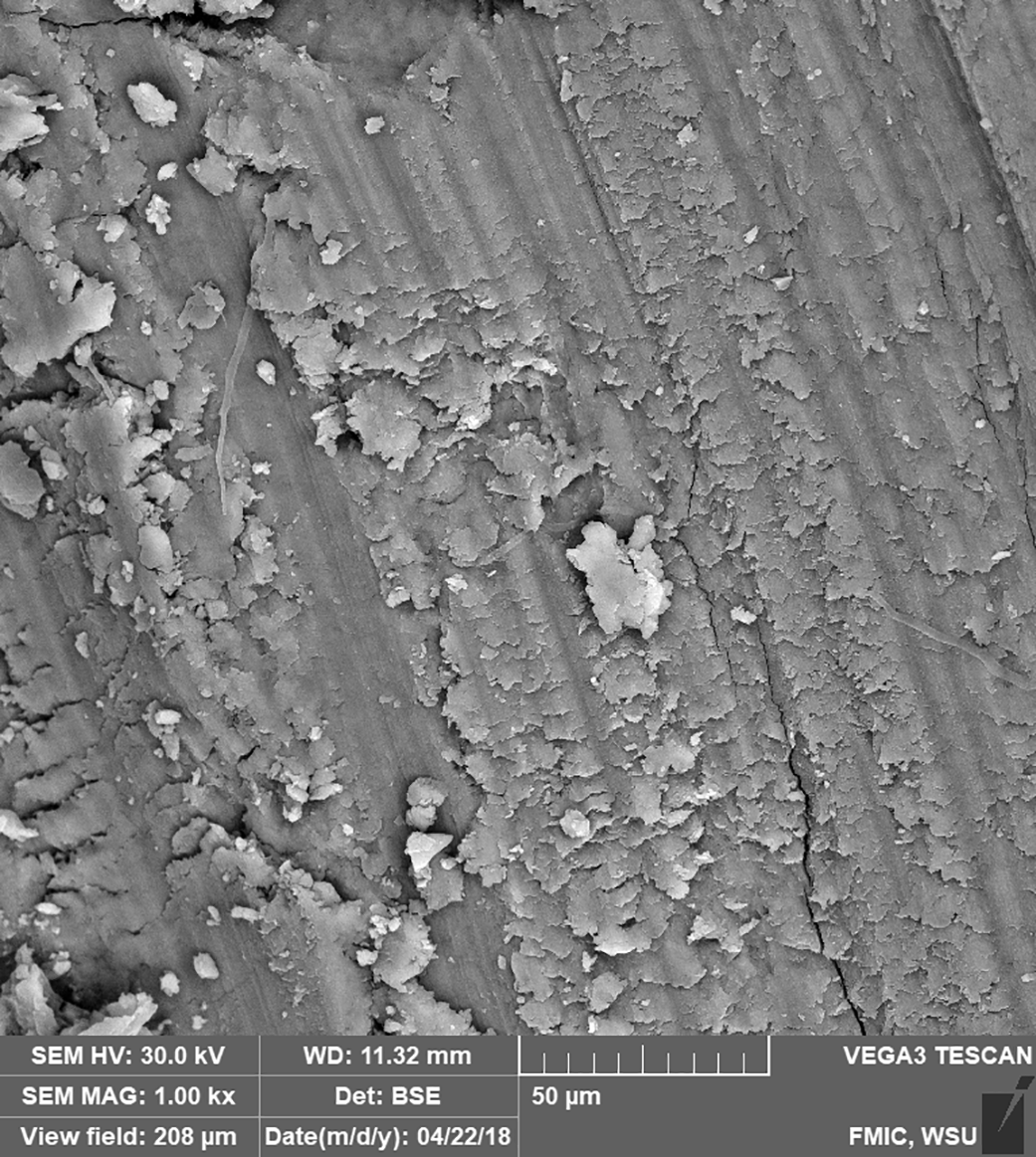

Supplement: S36 Fig — Image was taken approximately 0.47 mm from the upper edge of the drill hole. Image was captured on a Tescan Vega 3 SEM at 1,000x magnification. Bar = 50 μm. (TIF) [file pone.0201472.s038.tif]

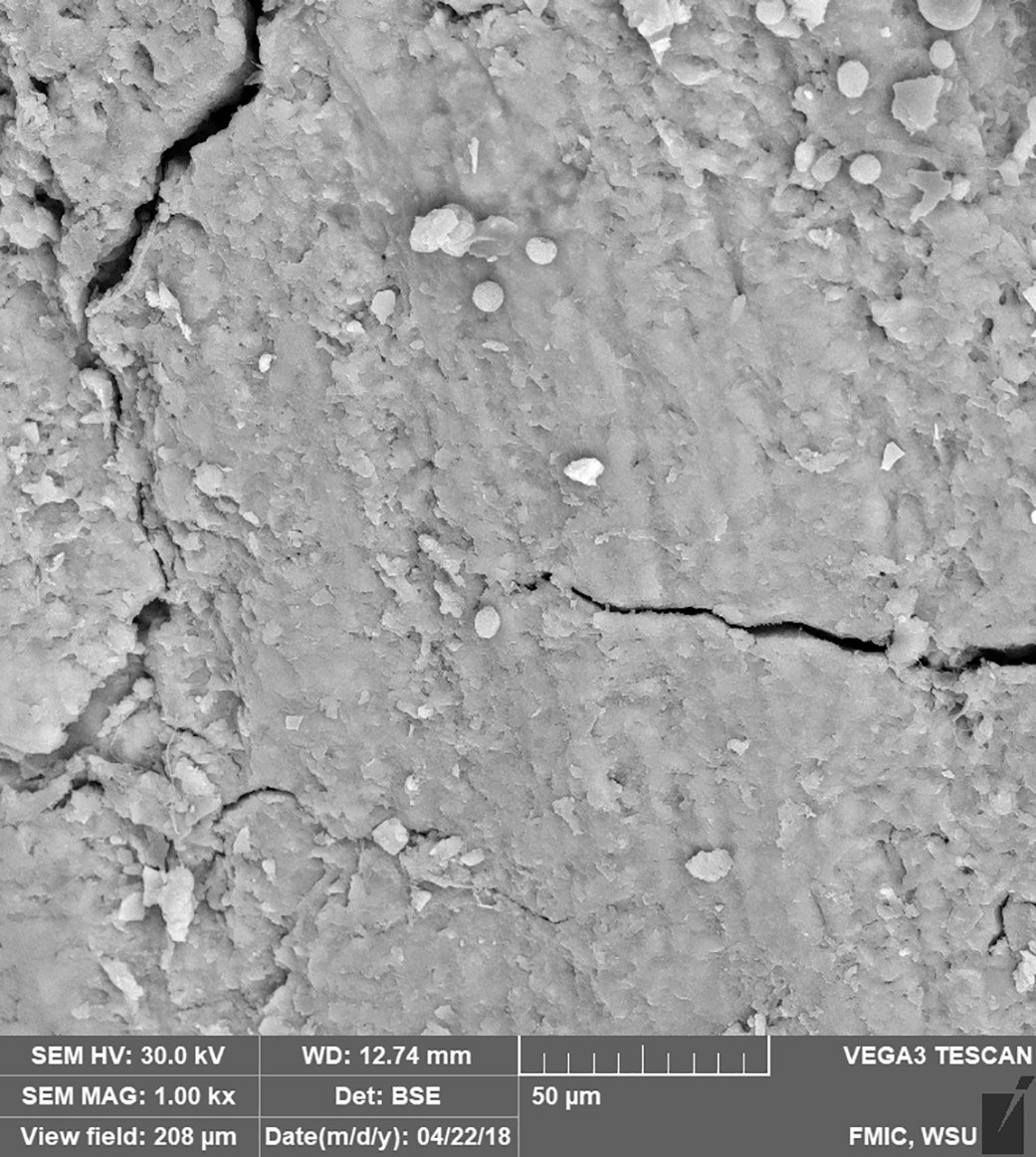

Supplement: S37 Fig — Image was taken approximately 1.89 mm from the upper edge of the drill hole. Image was captured on a Tescan Vega 3 SEM at 1,000x magnification. Bar = 50 μm. (TIF) [file pone.0201472.s039.tif]

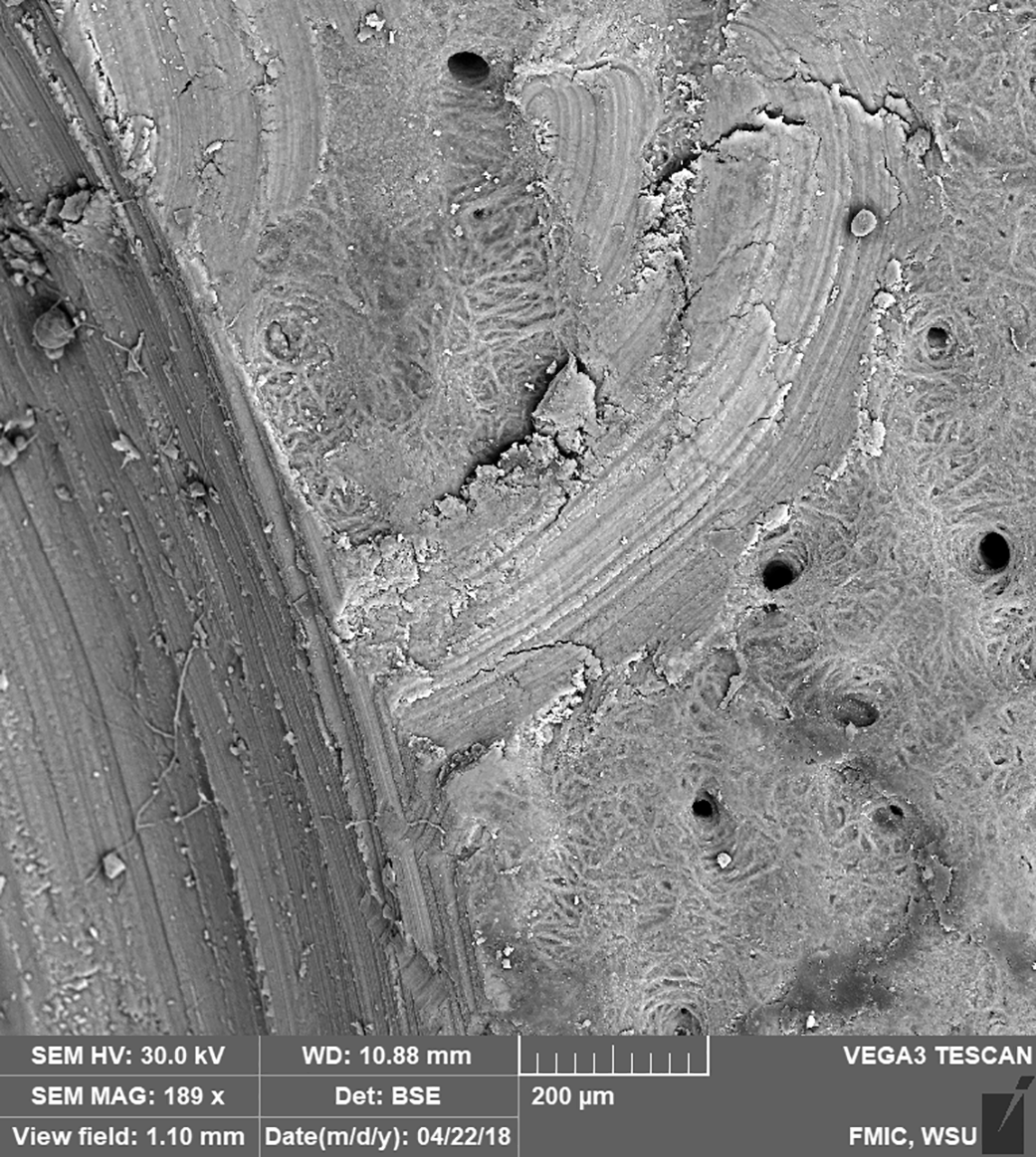

Supplement: S38 Fig — The image shows how the drill slipped during drilling or while starting the drilling process. Image was captured on a Tescan Vega 3 SEM at 189x magnification. Bar = 200 μm. (TIF) [file pone.0201472.s040.tif]

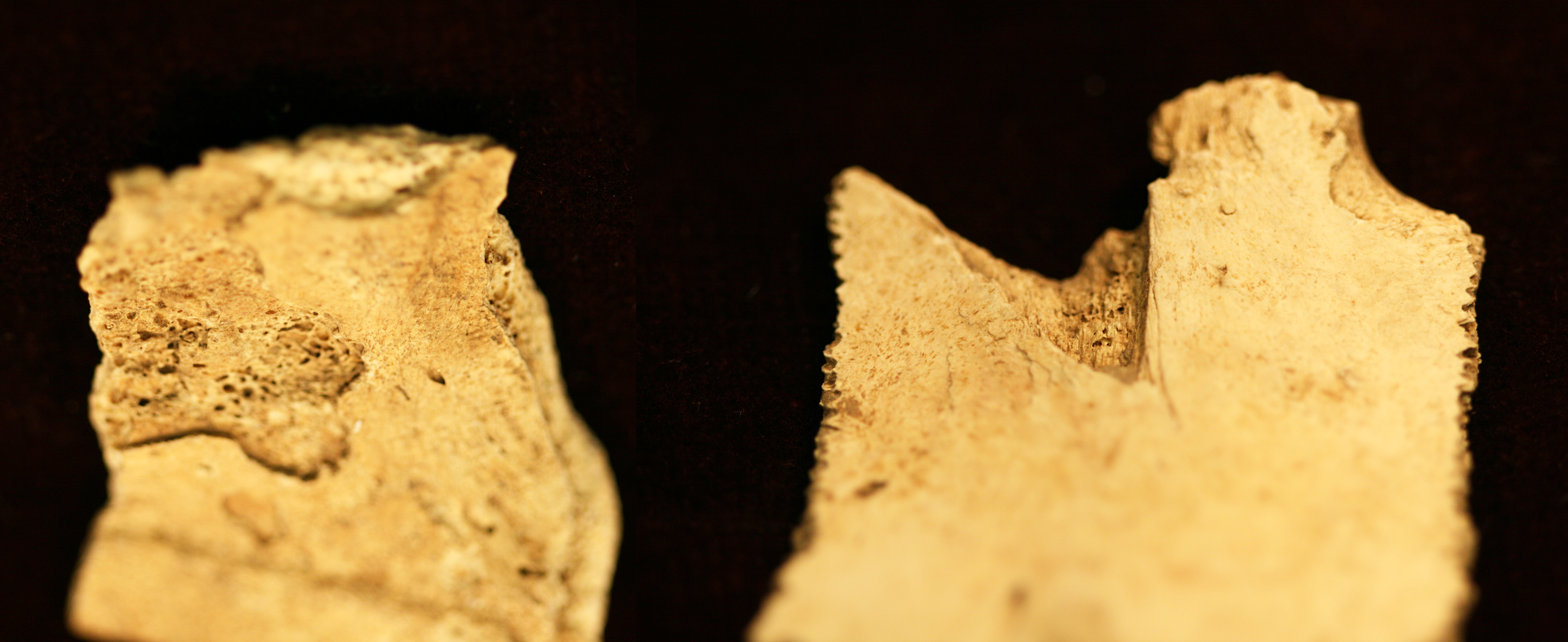

Supplement: S39 Fig — (TIF) [file pone.0201472.s041.tif]

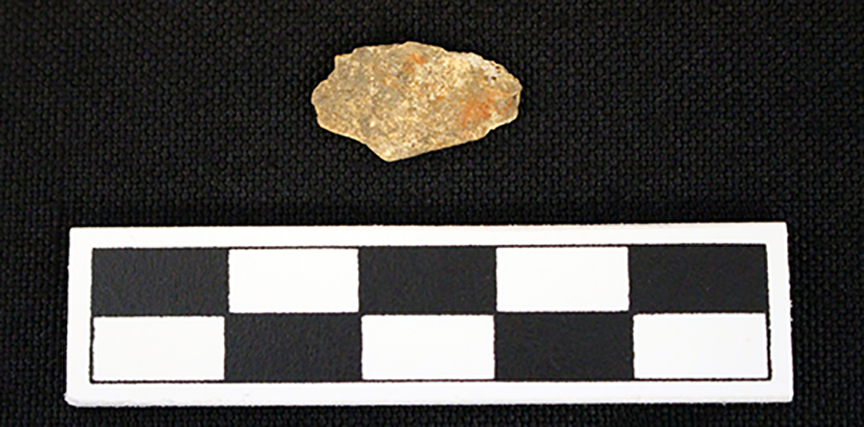

Supplement: S40 Fig — Image courtesy of the Tennessee Department of Transportation, Nashville. (TIF) [file pone.0201472.s042.tif]

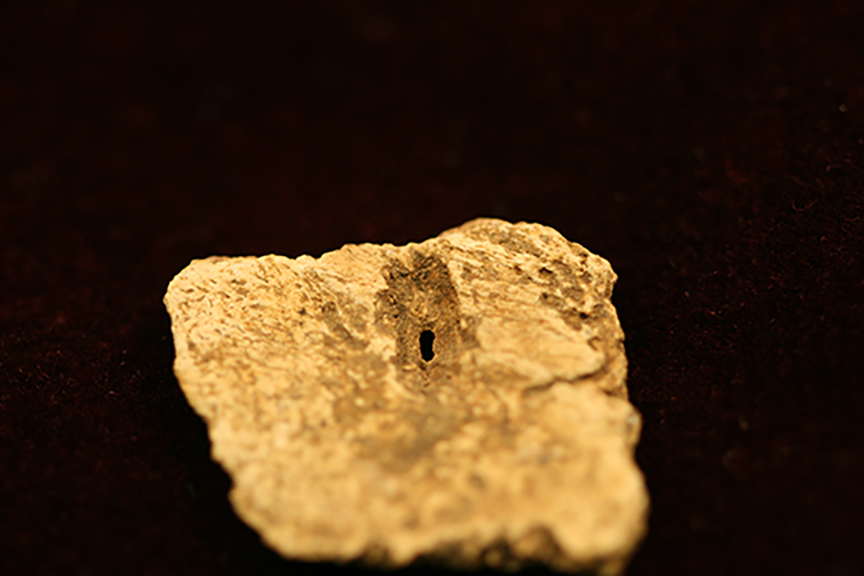

Supplement: S41 Fig — (TIF) [file pone.0201472.s043.tif]

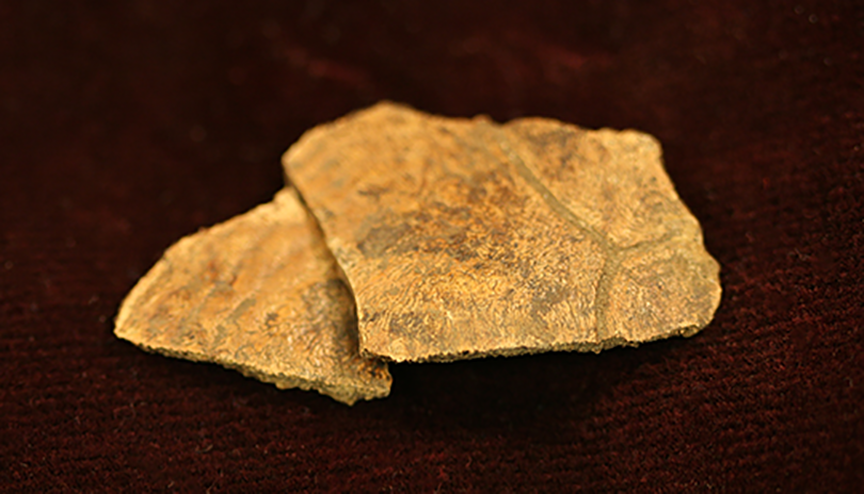

Supplement: S42 Fig — (TIF) [file pone.0201472.s044.tif]
